# Morphological and functional properties distinguish the substance P and gastrin-releasing peptide subsets of excitatory interneuron in the spinal cord dorsal horn

**DOI:** 10.1097/j.pain.0000000000001406

**Published:** 2018-09-20

**Authors:** Allen C. Dickie, Andrew M. Bell, Noboru Iwagaki, Erika Polgár, Maria Gutierrez-Mecinas, Rosalind Kelly, Heather Lyon, Kirsten Turnbull, Steven J. West, Alexander Etlin, Joao Braz, Masahiko Watanabe, David L.H. Bennett, Allan I. Basbaum, John S. Riddell, Andrew J. Todd

**Affiliations:** a Spinal Cord Group, Institute of Neuroscience and Psychology, College of Medical, Veterinary and Life Sciences, University of Glasgow, Glasgow, United Kingdom; b The Nuffield Department of Clinical Neurosciences, University of Oxford, John Radcliffe Hospital, Oxford, United Kingdom; c Department of Anatomy, University of California, San Francisco, San Francisco, CA, United States; d Department of Anatomy, Hokkaido University School of Medicine, Sapporo, Japan

**Keywords:** Glutamatergic interneuron, Radial cell, Central cell, GRP, Pain

## Abstract

Excitatory interneurons account for the majority of neurons in the superficial dorsal horn, but despite their presumed contribution to pain and itch, there is still limited information about their organisation and function. We recently identified 2 populations of excitatory interneuron defined by expression of gastrin-releasing peptide (GRP) or substance P (SP). Here, we demonstrate that these cells show major differences in their morphological, electrophysiological, and pharmacological properties. Based on their somatodendritic morphology and firing patterns, we propose that the SP cells correspond to radial cells, which generally show delayed firing. By contrast, most GRP cells show transient or single-spike firing, and many are likely to correspond to the so-called transient central cells. Unlike the SP cells, few of the GRP cells had long propriospinal projections, suggesting that they are involved primarily in local processing. The 2 populations also differed in responses to neuromodulators, with most SP cells, but few GRP cells, responding to noradrenaline and 5-HT; the converse was true for responses to the μ-opioid agonist DAMGO. Although a recent study suggested that GRP cells are innervated by nociceptors and are strongly activated by noxious stimuli, we found that very few GRP cells receive direct synaptic input from TRPV1-expressing afferents, and that they seldom phosphorylate extracellular signal–regulated kinases in response to noxious stimuli. These findings indicate that the SP and GRP cells differentially process somatosensory information.

## Abstract 2

Supplemental Digital Content is Available in the Text.

Superficial dorsal horn excitatory interneuron populations, as identified by neuropeptide expression, differ in morphological, electrophysiological, and pharmacological properties. This has implications for understanding pain processing.

## 1. Introduction

The superficial laminae (I-II) of the spinal dorsal horn are the main target for nociceptive and pruriceptive primary afferents. Consequently, neurons within this region are involved in transmitting and modulating signals perceived as pain and itch. Although some of these cells project to the brain, most (∼99%) are interneurons.^1,8,65^ Although early models emphasised the importance of inhibitory interneurons in gating nociceptive inputs,^45^ the majority of interneurons in these laminae are glutamatergic excitatory cells,^66^ and these are both functionally and morphologically heterogeneous.^22,49,56,71,73^ Recent studies demonstrated that the excitatory interneurons are essential for normal perception of pain and itch, and suggested that particular subgroups process distinct sensory modalities.^16,69^ It is therefore important to define functional populations among these cells.

Among the morphologically distinct classes of excitatory interneurons are vertical cells, which have ventrally directed dendrites; radial cells, which have compact, highly branched dendritic trees; and central cells, which have rostrocaudally orientated dendrites. These differ in physiological properties^22,71^ and may therefore correspond to functional populations. However, many excitatory interneurons cannot be assigned to a particular morphological class,^73^ and we have developed an alternative classification scheme based on the largely nonoverlapping expression of 4 neuropeptides: neurotensin, neurokinin B (NKB), gastrin-releasing peptide (GRP), and substance P (SP).^23,24^ Together, these account for over half of the excitatory interneurons in laminae I and II. Neurotensin and NKB neurons are concentrated in inner lamina II (IIi), and overlap extensively with cells expressing protein kinase Cγ (PKCγ). Gastrin-releasing peptide–expressing neurons are found throughout lamina II and can been identified in a GRP::eGFP BAC transgenic mouse.^57^ Gastrin-releasing peptide cells have attracted particular interest because of their contribution to itch.^46,60^ Gastrin-releasing peptide released from these cells targets the GRP receptor (GRPR), which is expressed by a different population of excitatory interneurons in lamina I and outer lamina II (IIo).^61,62^ Gastrin-releasing peptide cells are innervated by pruriceptive afferents that express MrgA3, a receptor for the pruritogen chloroquine,^60^ and are believed to act as “secondary pruriceptors” in a pathway linking pruriceptive afferents to GRPR-expressing interneurons,^46^ which innervate lamina I projection neurons. Substance P is cleaved from a precursor, preprotachykinin A (PPTA) coded by *Tac1*, and SP-expressing neurons can be identified by intraspinal injection of adeno-associated viruses (AAVs) containing Cre-dependent expression cassettes into mice in which Cre-recombinase is knocked into the *Tac1* locus (Tac1^Cre^). Using this approach, we found that SP-expressing cells are located mainly in lamina IIo, somewhat dorsal to GRP cells, although the populations show some spatial overlap.^23^ Although SP-expressing neurons include some projection neurons and inhibitory cells, the great majority are excitatory interneurons.^23,25^

Despite the nonoverlapping expression pattern of these neuropeptides, we cannot be certain that GRP and SP cells represent distinct functional populations. Here, we used a variety of techniques to characterise and compare GRP- and SP-expressing neurons in the mouse superficial dorsal horn. We find that these 2 populations differ widely in anatomical, electrophysiological, and pharmacological properties, suggesting that they represent distinct populations that are likely to contribute differentially to somatosensory processing.

## 2. Methods

Experiments were approved by the Ethical Review Process Applications Panel of the University of Glasgow and were performed in accordance with the UK Animals (Scientific Procedures) Act 1986 and the University of California, San Francisco's Institutional Animal Care and Use Committee guidelines.

### 2.1. Animals

We used 2 genetically modified mouse strains: a BAC transgenic Tg(GRP-EGFP) from GENSAT in which GFP is expressed under control of the GRP promoter,^19,26,46,57^ and a line in which Cre-recombinase is inserted into the *Tac1* locus (Tac1-IRES2-Cre-D; Jackson Laboratory, Bar Harbor, ME; Stock number 021877).^28^ These lines are referred to as GRP::eGFP and Tac1^Cre^, respectively. GRP::eGFP mice were maintained as heterozygotes, whereas most of the Tac1^Cre^ mice were homozygous. Both strains were maintained on a C57BL/6 background. For some experiments, the 2 lines were crossed to produce double transgenic mice (Tac1^Cre^;GRP::eGFP). Unless otherwise stated, mice of either sex weighing between 14 and 28 g were used in all parts of the study. Most of the mice that were used for anatomical studies underwent perfusion fixation. They were deeply anaesthetised with pentobarbitone (20 mg, intraperitoneally [i.p.]) and perfused through the heart with a fixative that contained 4% freshly depolymerised formaldehyde in phosphate buffer. Spinal cord tissue was rapidly dissected out and postfixed at 4°C for 2 hours (unless stated otherwise).

### 2.2. Intraspinal injection

Intraspinal injections were performed to deliver viral vectors coding for Cre-dependent constructs into Tac1^Cre^ or Tac1^Cre^;GRP::eGFP mice, and to deliver the retrograde tracer cholera toxin B (CTb) subunit into GRP::eGFP mice. Table 1 lists the viral vectors used. To identify SP cells, we used AAVs coding for Cre-dependent eGFP or tdTomato, and to investigate the somatodendritic morphology of these cells, we used AAV-Brainbow vectors.^9^ The injections used a modification of the method of Foster et al.^17^ as described previously.^23^ The mice were anaesthetised with 1% to 2% isoflurane and placed in a stereotaxic frame. For the experiments involving AAVs, 2 injections were made in each animal, either into the L3 and L5 segments on one side, or else bilaterally into either the L3 or L5 segments. The vertebral column was exposed, and vertebral clamps were attached to the T12 and L1 vertebrae. The space between the laminae of T12 and T13 was used for L3 injections and that between laminae of T13 and L1 for L5 injections. In each case, a small incision was made in the dura to the side of the midline, and injections were made through glass micropipettes (inner diameter of tip 40 μm) into the spinal dorsal horn. Injections were made 300 to 500 μm lateral to the midline at a depth of 300 μm below the pial surface and were administered at a rate of 30 nL/minute. The wound was then closed, and animals were allowed to recover with appropriate analgesia (buprenorphine 0.3 mg/kg and carprofen 5 mg/kg). Injections of CTb into the GRP::eGFP mice were targeted on the T13 spinal segment. These injections were performed as described above, except that they were made through the space between the laminae of T11 and T12 vertebrae.^25^ In each case, a single injection (300 nL of 1% CTb) was made into the dorsal horn on the right side, and the animals survived for 4 days before perfusion fixation.^25^

**Table 1:**
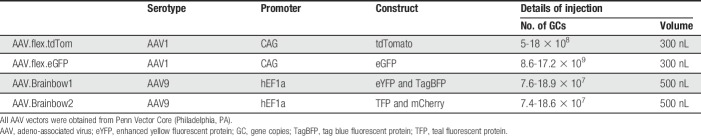
Adeno-associated virus vectors.

### 2.3. General features of immunocytochemistry and characterisation of antibodies

Multiple labelling immunofluorescence microscopy was performed as described previously.^23^ Table 2 lists the sources and concentrations of the antibodies used. Briefly, spinal cord segments were cut into 60-μm-thick transverse or parasagittal sections with a vibrating blade microtome (Leica VT1200, Leica Microsystems (UK) Ltd, Milton Keynes, United Kingdom), and these were treated with 50% ethanol for 30 minutes to enhance antibody penetration. Sections were incubated for 3 days at 4°C in mixtures of primary antibodies and then overnight in mixtures of species-specific secondary antibodies that were raised in donkey. The secondary antibodies were conjugated to Alexa 488, Alexa 647, Rhodamine Red, Pacific Blue, or biotin (Jackson ImmunoResearch, West Grove, PA) and were used at 1:500 (Alexa 488, Alexa 647, and biotin), 1:200 (Pacific Blue), or 1:100 (Rhodamine Red). Biotinylated secondary antibodies were detected with a tyramide signal-amplification method (TSA kit tetramethylrhodamine NL702; PerkinElmer Life Sciences, Boston, MA) as described previously.^24^ After immunoreaction, the sections were mounted in antifade medium and stored at −20°C. Unless otherwise stated, sections were scanned with a Zeiss LSM710 confocal microscope equipped with Argon multi-line, 405 nm diode, 561 nm solid state, and 633 nm HeNe lasers. In all cases, the confocal aperture was set to 1 Airy unit or less.

**Table 2:**
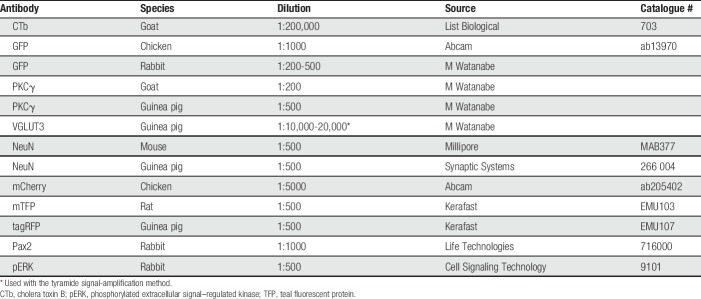
Antibodies.

The CTb antibody was raised against the purified protein, and specificity is demonstrated by the lack of staining in regions that did not contain injected or transported tracer. The chicken and rabbit antibodies against GFP were raised against recombinant full-length eGFP, and the staining matches that of native GFP fluorescence. The PKCγ antibodies, which were raised against amino acids 648 to 697 of the mouse protein, detect a single band at 75 kDa in tissue from wild-type (but not PKCγ^−/−^) mice, and the guinea pig antibody stains identical structures to those detected by a well-characterised rabbit antibody.^52,74^ The VGLUT3 antibody was raised against amino acids 522 to 588 of the mouse protein and detects a single band at 60 to 62 kDa. The mouse monoclonal NeuN antibody was raised against cell nuclei extracted from the mouse brain and found to react with a protein specific for neurons,^47^ which has subsequently been identified as the splicing factor Fox-3.^36^ The guinea pig antibody was raised against a recombinant protein consisting of amino acids 1 to 97 of Fox-3 and immunostains the same cells as the mouse antibody. The mCherry antibody was raised against a full-length recombinant protein, whereas the teal fluorescent protein (TFP) and tagRFP antibodies were raised against the corresponding purified proteins. Their specificity is demonstrated by the lack of staining in tissue that lacks these fluorescent proteins. The Pax2 antibody is directed against amino acids 188 to 385 of the mouse protein and recognizes bands of the appropriate size on Western blots of the mouse embryonic kidney.^15^ The phosphorylated extracellular signal–regulated kinase (pERK) antibody detects p44 and p42 MAP kinase (Erk1 and Erk2) when these are phosphorylated either individually or dually at Thr202 and Tyr204 of Erk1 or Thr185 and Tyr187 of Erk2. This antibody does not cross-react with the corresponding phosphorylated residues of JNK/SAPK or of p38 MAP kinase, or with nonphosphorylated Erk1/2 (manufacturer's specification). Specificity is demonstrated by the lack of staining in nonstimulated areas (eg, in the contralateral dorsal horn).

### 2.4. Distribution of gastrin-releasing peptide::eGFP cells and their relation to gastrin-releasing peptide mRNA

To assess differences in the distribution of GRP-eGFP cells in different regions of the lumbar spinal cord, we examined the lateral and medial regions of the superficial dorsal horn in the L1 and L4 segments using immunostained sections. These segments were chosen because they differ in the extent to which they receive input from glabrous skin,^30^ and preliminary observations indicated that GFP^+^ cells were less numerous in glabrous skin territory. Scans were analysed with Neurolucida for Confocal (MBF Bioscience, Williston, VT). We used a modification^48^ of the disector method^59^ to quantify the proportion of all neurons that were GFP-positive in sections from 3 perfusion-fixed GRP::eGFP mice. These sections had been immunoreacted to reveal GFP (chicken antibody), VGLUT3, and NeuN (mouse antibody), and counterstained with 4′,6-diamidino-2-phenylindole (DAPI). Three sections from the L1 and L4 segments of each mouse were scanned with the confocal microscope through a 40× oil-immersion lens (numerical aperture 1.3) to include the entire mediolateral extent of the dorsal horn through the whole depth of the section (2-μm z-spacing). The reference and look-up sections were placed 20 μm apart, and all optical sections between these were carefully examined. All neuronal nuclei (defined by the presence of NeuN and DAPI) with their bottom surface between the reference and look-up sections were identified and plotted onto an outline of the dorsal horn. The channel corresponding to GFP was then viewed, and the presence or absence of immunoreactivity was noted for each selected neuron. Scans from L4 were divided into medial and lateral compartments based on the pattern of VGLUT3 immunoreactivity, which is expressed by C–low-threshold mechanoreceptors that are restricted to the lateral “hairy skin” region.^38,54^ Because the whole of the L1 segment is innervated from hairy skin, we divided the dorsal horn into medial and lateral halves in this segment.

In situ hybridisation was used to examine the relationship between GRP mRNA and GFP in the L4 and L5 segments from 3 perfusion-fixed GRP::eGFP mice. Tissue from these mice was postfixed for 4 to 12 hours, cryoprotected in 30% sucrose, embedded in optimal cutting temperature mounting medium, and stored at −80°C. Transverse sections (12-μm thick) were cut with a cryostat, mounted onto SuperFrost Plus slides (48311-703, VWR), and air dried. The RNAscope procedure was performed according to the supplied protocol. Briefly, tissue was incubated in peroxide solution, dehydrated in 100% ethanol, and reacted with protease in aqueous buffer. The RNAscope probe against GRP (Mm-GRP, 317861, RNAscope, Bio-Techne, Abingdon, United Kingdom) and a negative control probe were incubated with tissue sections at 40°C for 2 hours, followed by washing in RNAscope wash buffer and a series of incubations in amplification buffers (1-6, 15-30 minutes at 40°C—room temperature), and finally detected using the Red Assay kit (322360; RNAscope). Sections were then incubated for 24 hours at room temperature in GFP antibody (rabbit), and then in secondary antibody conjugated to Alexa 488. They were then counterstained with DAPI and mounted in antifade medium. Sections were scanned with a Zeiss LSM 700 laser-scanning confocal microscope. The images were acquired by scanning a single optical plane through the centre of the section depth with a 40× oil-immersion lens (numerical aperture 1.3), to include both dorsal horns for each section.

Cells that were positive for GRP mRNA and GFP were quantified manually in ImageJ by using the Cell Counter Plugin. Cells in laminae I and II of the dorsal horn were counted, and a minimum of 3 sections were assessed per animal. The dorsal horn was divided into medial glabrous and lateral hairy skin innervation territories, defined by the intense plexus of GFP labelling seen in hairy skin territory in the GRP:eGFP mice (see below). Initially, the mRNA channel was examined, and cell bodies (defined by DAPI labelling) that contained 3 or more intense mRNA puncta were counted. Next, the GFP labelling was viewed to reveal the proportion of GFP cells that were positive for GRP mRNA. Finally, the number of GFP-positive cells that lacked GRP mRNA was determined. Images were assessed against the negative control probe, which did not show any specific or intense in situ hybridisation product.

### 2.5. Relationship between gastrin-releasing peptide– and substance P–expressing cells

To determine the extent of overlap between GRP::eGFP and SP populations, we injected AAV coding for a Cre-dependent form of tdTomato (AAV.flex.tdTom) into 2 Tac1^Cre^;GRP::eGFP male mice. The virus encodes an inverted sequence for tdTomato between pairs of heterotypic LoxP sites with antiparallel orientation,^4^ resulting in expression of tdTomato only in transfected cells that contain Cre. After a 2-week survival period, the mice were reanaesthetised and perfused with fixative. Sections through the injection site of both mice were immunostained with antibodies against GFP (rabbit) and PKCγ (goat), and 2 sections containing numerous tdTom^+^ cells were selected and scanned with the confocal microscope. The scans were analysed with Neurolucida. Initially, the channel corresponding to tdTom was viewed, and all labelled cells were plotted onto an outline of the superficial dorsal horn (laminae I and II). The other 2 channels were then viewed, and the presence or absence of GFP and PKCγ was noted for each tdTom cell. Any additional cells that were GFP^+^ and/or PKCγ^+^ were then added to the drawing. Although a stereological method was not used in this part of the study, the low level of double labelling for tdTom and GFP (see below) is unlikely to have been affected by sampling bias. We have previously shown that after intraspinal injection of AAV coding for a Cre-dependent form of eGFP into Tac1^Cre^ mice, virtually all the cells near the injection site that are immunoreactive for the SP precursor PPTA are GFP-positive, indicating that this procedure captures a high proportion of SP-expressing cells.^23^ We have also reported that it results in GFP labelling of ∼20% of neurons in the superficial dorsal horn within 4 days of the injection, and that this percentage does not change when animals are allowed to survive for 8 days after the spinal injection,^25^ suggesting that a stable expression pattern is reached very soon after the injection.

To compare expression of SP with the distribution of GRP mRNA, we injected AAV.flex.eGFP into 3 male Tac1^Cre^ mice. One or 2 weeks later, the mice were reanaesthetised and fixed by perfusion. Tissue from these mice was processed with in situ hybridisation histochemistry to reveal GRP mRNA and GFP. Both the tissue processing and analysis were performed as described above.

To provide further evidence about the extent of coexpression of SP and GRP, we performed double-label fluorescence in situ hybridisation on tissue from 4 adult wild-type (C57Bl/6) mice, using RNAscope probes against mRNAs for GRP and substance P. The reaction was performed on 12-μm-thick cryostat sections from C4 to C6 segments (from 3 mice) and the L3 to L5 segment (from one mouse) according to the supplied protocol. Sections were counterstained with DAPI to allow for identification of cell nuclei. Single RNA transcripts for each target gene appeared as punctate dots (usually around the DAPI-stained nucleus), and we considered a cell to be positive if it contained more than 5 dots. Quantification was performed on a series of spinal cord sections (1 in every 4) from 3 separate animals. We analysed between 5 and 8 sections per animal. Only positive cells containing a DAPI-stained nucleus in the dorsal horn (laminae I-V) were included in the analysis. The percentage of double-labeled cells was calculated by dividing the number of double-labeled neurons by the number of single-labeled neurons for each probe.

### 2.6. Slice preparation and electrophysiology

Electrophysiological recordings from both SP and GRP neurons were performed on spinal cord slices. For those involving SP neurons, we used Tac1^Cre^ mice that had received intraspinal injection of either AAV.flex.eGFP or AAV.flex.tdTom between 1 and 3 weeks previously. Spinal cord slices were obtained from 40 injected Tac1^Cre^ mice and from 105 GRP::eGFP mice aged 4 to 10 weeks old, as described previously.^14,34^ The spinal cord was removed either after laminectomy performed under isoflurane anaesthesia or in ice-cold dissection solution after decapitation of the mice under brief isoflurane or tribromoethanol anaesthesia. Mice from which the cord was removed under anaesthesia were decapitated immediately afterwards. Parasagittal (300-500 μm), transverse (400-600 μm), or horizontal (400 μm) slices from the lumbar spinal cord were cut with a vibrating blade microtome and allowed to recover in recording or modified dissection solution for at least 30 minutes at room temperature.^14,34^ In some cases, the slices were placed in an NaCl-based recovery solution or an *N*-methyl-d-glucamine (NMDG)-based recovery solution^64^ at 32°C for 15 minutes before being placed in recording solution at room temperature. The solutions used contained the following (in mM): dissection, 3.0 KCl, 1.2 NaH_2_PO_4_, 0.5 to 2.4 CaCl_2_, 1.3 to 7.0 MgCl_2_, 26.0 NaHCO_3_, 15.0 to 25.0 glucose, and 240.0 to 251.6 sucrose; modified dissection, 3.0 KCl, 1.2 NaH_2_PO_4_, 0.5 CaCl_2_, 1.3 MgCl_2_, 8.7 MgSO_4,_ 26.0 NaHCO_3_, 20.0 HEPES, 25.0 glucose, and 215.0 sucrose, with or without 1.0 kynurenic acid; NaCl recovery solution, 125.0 NaCl, 2.5 KCl, 1.25 NaH_2_PO_4_, 1.5 CaCl_2_, 6.0 MgCl_2_, 26.0 NaHCO_3_, and 25.0 glucose; NMDG recovery solution, 93.0 NMDG, 2.5 KCl, 1.2 NaH_2_PO_4_, 0.5 CaCl_2_, 10.0 MgSO_4_, 30.0 NaHCO_3_, 25.0 glucose, 5.0 Na-ascorbate, 2.0 thiourea, 3.0 Na-pyruvate, and 20.0 HEPES; and recording, 125.0 to 127.0 NaCl, 2.5 to 3.0 KCl, 1.2 to 1.25 NaH_2_PO_4_, 2.0 to 2.4 CaCl_2_, 1.0 to 1.3 MgCl_2_, 26.0 NaHCO_3_, and 15.0 to 25.0 glucose. In some cases, dissection was performed in NaCl recovery solution with 1 mM kynurenic acid. All solutions were bubbled with 95% O_2_/5% CO_2_.

Targeted whole-cell patch-clamp recordings were made from GFP-positive cells (slices from GRP::eGFP mice or Tac1^Cre^ mice with AAV.Flex.eGFP injection) or tdTomato-positive cells (Tac1^Cre^ mice with AAV.Flex.tdTom injection) in the superficial dorsal horn, using patch pipettes that had a typical resistance of 3 to 7 MΩ when filled with an intracellular solution containing (in mM): 130.0 K-gluconate, 10.0 KCl, 2.0 MgCl_2_, 10.0 HEPES, 0.5 EGTA, 2.0 ATP-Na_2_, 0.5 GTP-Na, and 0.2% Neurobiotin, pH adjusted to 7.3 with 1.0M KOH. In some cases, the pipette solution contained (in mM): 120.0 K-methanesulphonate, 10.0 NaCl, 1.0 CaCl_2_, 10.0 HEPES, 10 EGTA, 5.0 ATP-Mg, and 0.5 GTP-Na. Data were recorded and acquired with a Multiclamp 700B amplifier and pClamp 10 software (both Molecular Devices), and were filtered at 4 kHz and digitised at 10 kHz.

After achieving stable whole-cell configuration, the cells were voltage clamped at −60 mV, and a series of 100-millisecond voltage steps from −70 to −50 mV (2.5 mV increments) delivered to determine the current–voltage relationship, which was used to calculate resting membrane potential and input resistance. Cells that had a resting membrane potential that was less negative than −30 mV were excluded from all analysis.

The action potential firing pattern was assessed in the current-clamp mode, in response to 1-second depolarising current steps of increasing amplitude, from a membrane potential of around −60 mV. Firing patterns were classified on the basis of previously published criteria.^18,20,22,51,56,73^ Cells were classed as tonic firing if they exhibited continuous action potential discharge throughout the depolarising step; transient if the action potential discharge occurred only during the early part of the step; delayed if there was a clear delay between the start of the depolarising step and the first action potential; single spike if only 1 or 2 action potentials occurred at the onset of the step; gap if there was a long first interspike interval; and reluctant if current injection did not result in action potential firing. It has recently been reported that reluctant firing may result from high levels of expression of both a low-threshold noninactivating potassium conductance and an inactivating (A-type) potassium conductance.^5^

Subthreshold voltage-activated currents were investigated by voltage clamping the cell at −60 mV before stepping to −90 mV for 1 second and then to −40 mV for 200 milliseconds, and automated leak subtraction was used to remove capacitive and leak currents.^21,56^ This step protocol enables the identification of 2 types of transient outward current and 2 types of inward current.^21^ The outward currents that occur during the depolarising step (−90 to −40 mV) are consistent with A-type potassium currents (I_A_), and on the basis of their kinetics can be distinguished as rapid (I_Ar_) or slow (I_As_). A transient inward current can be observed during the depolarising step, which is considered to reflect the low-threshold “T-type” calcium current (I_ca,T_). A slow inward current can occur during the hyperpolarisation step (−60 to −90 mV) that is consistent with the hyperpolarisation-activated (I_h_) current. The amplitude of I_Ar_ was measured as the peak of the transient outward current. The amplitude of the I_h_ current was measured during the final 200 milliseconds of the hyperpolarising step, and inward currents were classified as I_h_ if the amplitude was greater than −5 pA.

Excitatory synaptic input to GRP-eGFP and SP neurons was assessed by recording spontaneous excitatory postsynaptic currents (sEPSCs) at a holding potential of −60 or −70 mV. The functional expression of TRP channels on the afferents providing synaptic input was investigated by voltage-clamping cells at −60 or −70 mV and recording sEPSCs or miniature EPSCs (mEPSCs), the latter in the presence of tetrodotoxin (TTX) (0.5 μM), bicuculline (10 μM), and strychnine (5 μM), before and during the bath application of the TRPV1 agonist capsaicin (2 μM) or the TRPM8 agonist icilin (20 μM; mEPSCs only). In the case of icilin application, the temperature of the bath was raised to 32°C.^18^ These data were analysed using Mini Analysis (Synaptosoft), and sEPSC/mEPSC events were automatically detected by the software and were then rejected or accepted after visual examination. Neurons were considered to receive input from capsaicin- or icilin-sensitive afferents if capsaicin/icilin application resulted in a significant leftwards shift in the distribution of interevent intervals, indicating an increase in frequency. They were considered nonresponsive if this threshold was not reached.

The response of GRP-eGFP and SP cells to a number of pharmacological agents was investigated by voltage-clamping cells at −60 or −70 mV, and bath applying one of the following: 5-HT (10-20 μM), noradrenaline (NA) (20 μM), the μ-opioid receptor (MOR) agonist DAMGO (3 μM), the κ-opioid (KOR) agonist U69593 (1 μM), or the δ-opioid (DOR) agonist [D-Ala^2^]-Deltorphin II (1 μM). Cells were considered responsive if drug application resulted in a clear slow outward current and nonresponsive if no current was seen.

All chemicals were obtained from Sigma except: TTX (Alomone, Jerusalem, Israel), bicuculline (Tocris, Abingdon, UK), and Neurobiotin (Vector Labs Peterborough, UK).

### 2.7. Morphological analysis of gastrin-releasing peptide and substance P cells

Reconstruction of the GRP neurons (n = 45) was performed on Neurobiotin-labelled cells that had been recorded in electrophysiological experiments. For the SP neurons (n = 31), analysis of cell bodies and dendritic trees was performed on tissue from mice that had been injected with Brainbow viruses.^9^ In addition, a few Neurobiotin-labelled SP neurons (n = 12) from electrophysiological experiments were examined to allow for reconstruction of their local axonal arbors.

Processing to reveal Neurobiotin in patched cells was performed as described previously.^18,34^ Briefly, fixed slices containing recorded cells were incubated in avidin-rhodamine (1:1000; Jackson ImmunoResearch) in phosphate-buffered saline containing 0.3% Triton X-100 and mounted on slides. In a few cases, recordings had been made from Tac1^Cre^ mice that were injected with AAV.flex.tdTom, and in these cases, avidin-Alexa 488 was used instead of avidin-rhodamine. The sections were scanned with the confocal microscope through a 63× oil-immersion lens (numerical aperture 1.4) at 0.5-μm z-spacing. These scans included all the dendritic and axonal arbors that were visible at this stage. For each cell, the presence of GFP or tdTom was confirmed by scanning for the native protein. The dendritic and axonal arbors were reconstructed in Neurolucida. Axons could easily be distinguished from dendrites based on their thin nontapering profiles and the presence of irregularly spaced boutons. By contrast, dendrites showed progressive tapering and invariably gave rise to dendritic spines.^34^ Slices were then resectioned at 60-μm thickness with the vibrating blade microtome, and sections were kept in a serial order. Sections were examined with the confocal microscope, and if additional parts of the dendritic or axonal tree located deep within the slice were found, these were added to the reconstruction. To determine laminar boundaries, we immunostained one section from each slice to reveal PKCγ, using a guinea pig antibody. PKCγ is present in a plexus of dendrites that occupies the inner half of lamina II (IIi).^31^ The boundaries between laminae II/III were then added to the reconstructions, by aligning sections containing the recorded cells with nearby sections stained for PKCγ. The lamina I/II border was taken to be 20 μm below the dorsal white matter,^18^ and this was also added to the reconstruction. To investigate the axonal morphology of SP neurons, we also reconstructed axonal arbors of 12 of these cells that had undergone whole-cell patch-clamp recording. These cells were processed, scanned, and analysed as described above, except that in this case, the PKCγ immunoreaction was performed on the intact slice.

Two Tac1^Cre^ mice (one male and one female) received injections of 2 Brainbow AAV vectors.^9^ One of these codes for enhanced yellow fluorescent protein and Tag blue fluorescent protein (TagBFP), and the other for TFP and mCherry. In both cases, the sequences are for farnesylated fluorescent proteins in reverse orientation between antiparallel LoxP sites. Cre-mediated recombination leads to random expression of one of the fluorescent proteins (or neither fluorescent protein) for each virus, and the presence of multiple copies of both viruses within cells results in a wide range of hues, resulting from varying amounts of each membrane-targeted fluorescent protein. Two weeks after the spinal injection, the mice were perfused with fixative, and tissue from these animals was used to investigate the morphology of SP-expressing neurons. Sagittal sections (60-μm thick) from the lumbar enlargement were reacted with antibodies against mCherry, mTFP, tagRFP (which recognises tagBFP), and Pax2, and these were revealed with secondary antibodies. Regions close to the injection site that contained numerous labelled cells were selected and scanned with the confocal microscope through the 63× lens at 0.5-μm z-spacing. Tile scans were obtained through the full thickness of the section to include approximately 400 μm along the rostrocaudal (RC) axis and the entire dorsoventral (DV) extent of laminae I and II. Thirty-one cells (15 and 16 from the 2 mice) were selected, based on the following criteria: (1) relatively strong staining for at least one of the fluorescent proteins; (2) location of the soma in the mid region of the z-axis, such that the entire dendritic tree was likely to be contained within the section; (3) the presence of a distinctive colour hue that contrasted with that of nearby labelled cells. Cell bodies and dendritic trees of SP neurons were drawn in Neurolucida by following colour-coded processes originating from the soma of the selected cells. Although this allowed for reconstruction of dendritic trees, it was not possible to follow axons beyond their initial segments, due to their very small diameter.

Morphometric data for dendritic trees of all the reconstructed cells were obtained from Neurolucida Explorer. The dendritic parameters extracted were the same as those used in our previous study.^18^ To make an objective comparison between the GRP and SP cells, we performed cluster analysis using Ward's method,^70^ as described previously.^18^ To reduce the dimensionality of the original data while preserving variance, we calculated principal components from the data set.^13^ The number of principal components to be retained for cluster analysis was then determined from a scree test.

### 2.8. Potential propriospinal projections of gastrin-releasing peptide–eGFP cells

Bice and Beal^7^ reported that a proportion of neurons in the superficial dorsal horn have long propriospinal axons. We recently reported that around 40% of excitatory interneurons in laminae I and II of the mouse L5 segment have axons that project at least 5 segments rostrally, and that SP cells are overrepresented among the neurons with these propriospinal projections.^25^ To determine whether GRP-expressing neurons also give rise to long propriospinal axons, we examined the L5 segments of 4 GRP::eGFP mice that had received injections of CTb into the T13 segment.

Injection sites were assessed by reacting transverse sections through T13 with an immunoperoxidase method.^10^ The sections were incubated in anti-CTb at 1:200,000, followed by biotinylated secondary antibody and avidin-peroxidase, which was revealed with diaminobenzidine in the presence of H_2_O_2_. Transverse sections from the L5 segments were reacted with antibodies against CTb, NeuN (mouse or guinea pig), and GFP (chicken). These antibodies were detected with fluorescent secondary antibodies, and the sections were stained with DAPI. Four sections from each mouse were selected for analysis before the relationship between CTb and GFP was observed. The sections were scanned through the 40× oil-immersion lens to generate z-series (at least 20 optical sections at 1 μm z-separation) such that the entire cross-sectional area of the ipsilateral dorsal horn was included. The confocal scans were analysed with Neurolucida by using the modified disector method (see above). The reference and look-up sections were set 10 μm apart. Because the quantitative analysis was performed on laminae I and II, we first plotted the outline of the dorsal horn gray matter, and then located the lamina II/III border, based on the relatively low density of neurons in lamina IIi. The channels corresponding to NeuN and DAPI were initially viewed, and all neurons for which the bottom surface of the nucleus lay between the reference and look-up sections were marked on the drawing. The GFP and CTb channels were then examined, and for each of the selected neurons, the presence or absence of both GFP and CTb was determined. In this way, we determined the proportions of all lamina I/II neurons, and of GRP-eGFP neurons, in the L5 segment that were retrogradely labelled with CTb.

Because few GRP cells in L5 were retrogradely labelled from the T13/L1 injection site (see Results), a more limited analysis was also performed on sections from the L2 segment of 2 of these mice, to look for evidence of short intersegmental projections.

### 2.9. Phosphorylation of extracellular signal–regulated kinase after noxious and pruritic stimuli

We previously demonstrated that SP cells in laminae I and II often express the transcription factor Fos^32^ or phosphorylate ERKs^35^ in response to a variety of noxious and pruritic stimuli.^23^ In addition, we reported that GRP cells seldom show pERK after injection of the pruritogen chloroquine.^6^ Here, we used phosphorylation of ERK to test whether the GRP cells respond to other pruritic or to noxious stimuli.

Fifteen GRP::eGFP mice were used in these experiments (n = 3 mice per stimulus type). In all cases, stimuli involved the left calf (which had been shaved on the day before stimulation), because of the relatively low density of GRP-eGFP cells in regions of the dorsal horn that are innervated from glabrous skin (see below). Twelve of the mice received noxious stimuli or vehicle injection. These animals had been anaesthetised with urethane (40-80 mg i.p.), and the stimulus was applied 5 minutes before perfusion fixation. The stimuli were: (1) immersion of the leg up to the level of the knee in 52°C water for 15 seconds (noxious heat); (2) pinching of 5 skin folds on the calf with forceps applied for 5 seconds at each site (pinch); (3) subcutaneous injection of capsaicin (10 μL) into the lateral surface of the calf (capsaicin); and (4) subcutaneous injection (10 μL) of the vehicle used to deliver capsaicin (vehicle). Capsaicin was initially prepared at 1% by dissolving in 7% Tween 80, 20% ethanol in saline, and then diluted to 0.25%. Three of the mice were anaesthetised with urethane and used to investigate phosphorylation of ERK after intradermal injection of histamine (100 μg/10 μL) into the lateral calf. The success of the intradermal injection was assessed by the formation of a small bleb in the calf skin.^50^ To avoid detecting pERK that had resulted from the intradermal injection itself, in these cases, perfusion fixation was performed 30 minutes after the stimulus. We have previously shown that intradermal injection of vehicle (phosphate-buffered saline) causes ERK phosphorylation when animals are perfused with fixative 5 minutes after the injection. This presumably results from the noxious stimulus caused by insertion of the needle and distension of the skin.^6^ By contrast, we found that injection of pruritogens, but not vehicle, resulted in pERK-immunoreactivity in neurons in laminae I and II when the mice were perfusion-fixed 30 minutes after the injection. This is likely to reflect the relatively prolonged activation of these cells by pruritogens.

The L3 spinal segment, which contains the great majority of cells activated by these stimuli, was cut into 60-μm-thick transverse sections, and these were reacted with antibodies against NeuN (mouse), GFP (chicken), and pERK. These markers were revealed with fluorescent secondary antibodies, and sections were stained with DAPI. Sections that contained numerous pERK-positive neurons were initially selected and scanned with the confocal microscope through the 40× oil-immersion lens, to generate z-stacks (2-μm z-separation) through the full thickness of the section so as to include the region that contained pERK cells. The z-stacks were analysed with Neurolucida. Initially, the outline of the gray matter was plotted, together with the ventral border of the GRP plexus (which corresponds approximately to the boundary between the inner and outer parts of lamina II). The mediolateral extent of the region that contained a high density of pERK cells was delineated by drawing 2 parallel lines that were orthogonal to the laminar boundaries. The channels corresponding to NeuN and DAPI were viewed, and the locations of all neurons that lay within this region were plotted onto the drawing. To avoid overcounting neurons, we included them if at least part of the nucleus (stained with DAPI) was present in the first optical section of the z-series and excluded them if part of the nucleus was present in the last optical section.^2,58^ The channel corresponding to pERK was then viewed, and the presence or absence of staining in each of the neurons in the sample was recorded. Finally, the GFP channel was viewed and all neurons that were GFP^+^ were identified on the drawing. As pERK^+^ cells were present at highest density in laminae I and IIo, we determined the proportion of all neurons that were located within this region and between the 2 parallel lines that were pERK-immunoreactive. We then determined the proportion of GFP^+^ neurons within this volume that were pERK-immunoreactive.

### 2.10. Terminology

For convenience, we refer to cells that expressed fluorescent proteins after intraspinal injections of AAVs coding for Cre-dependent forms of these proteins into the Tac1^Cre^ mouse line as “substance P (SP) cells.” Similarly, we refer to cells that were GFP-positive in the GRP::eGFP mouse as “GRP cells,” although not all cells with GRP mRNA were GFP-positive in this line (see below).

### 2.11. Statistics

Two-way repeated-measures ANOVA with the *post hoc* Sidak test was used to determine whether there were significant differences in the proportions of neurons that were GFP^+^ in medial and lateral parts of the dorsal horn at L1 and L4 in the GRP::eGFP mice and a *t* test was used to compare medial and lateral counts from in situ hybridisation data. Differences in electrophysiological properties between the GRP and SP cells were compared using Mann–Whitney *U* or Wilcoxon signed-rank tests. Recorded neurons were classified as responsive to capsaicin or icilin by comparing the cumulative probability distribution of sEPSC/mEPSC interevent intervals with a 2-sample Kolmogorov–Smirnov test. T tests were used to compare morphometric parameters from reconstructed axons and dendritic trees of GRP and SP populations. To determine whether there was a significant difference in the proportions of GRP^+^ and GRP^−^ neurons in L5 that were retrogradely labelled from T13/L1, a contingency table was analysed with the Mantel–Haenszel test.^44^ Breslow–Day testing for homogeneity of the odds ratio was conducted before computation of the Mantel–Haenszel odds ratio and 95% confidence intervals. The Mantel–Haenszel test was also used to determine whether the proportions of GRP^+^ and GRP-negative cells that showed pERK in response to noxious or pruritic stimuli differed significantly. Data are expressed as mean ± SEM, unless stated otherwise. *P* values of less than 0.05 were considered to be significant. Statistical tests were performed in Prism 7 (GraphPad) or SPSS (Version 22, IBM, for the Mantel–Haenszel test).

## 3. Results

### 3.1. Mediolateral distribution of gastrin-releasing peptide–eGFP cells

**Figure 1.**
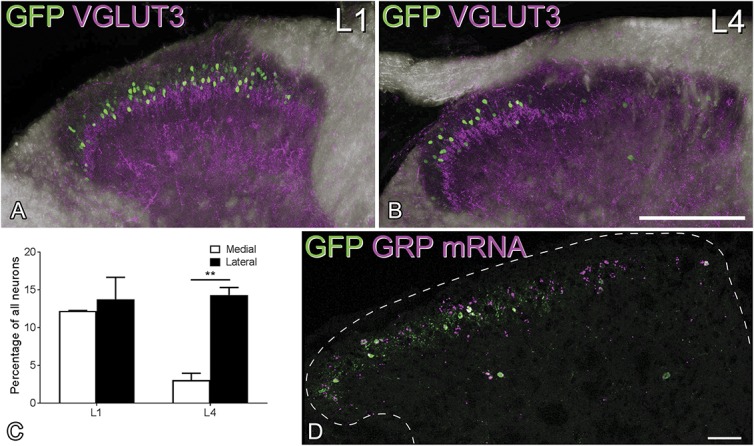
Differences in the mediolateral distribution of GFP and GRP mRNA in GRP::eGFP mice. (A and B) Immunostaining for GFP (green) and VGLUT3 (magenta) in the dorsal horn of GRP::eGFP mice in the L1 and L4 segments. A dense plexus of VGLUT3 staining is present in the inner part of lamina II, and this corresponds to the central terminals of C-LTMR afferents, which are associated with hairy skin. The plexus is evenly distributed across the L1 dorsal horn but is restricted to the lateral part in L4. The medial region, where the plexus is absent, is innervated from glabrous skin of the hind paw. Note that GFP^+^ cells are largely restricted to those regions with hairy skin input. These images are projections from z-stacks through the full thickness of 60-μm sections. (C) Quantification of GFP^+^ cells as a proportion of all neurons in the medial and lateral halves of the dorsal horn in L1 and the glabrous (medial) and hairy (lateral) territories in L4 (mean ± SD). ** denotes significant difference (*P*<0.01). (D) In situ hybridisation histochemistry on the L4 segment reveals that GRP mRNA (magenta) is present in many cells in the medial half of the dorsal horn, although there are very few GFP^+^ (green) cells. The dashed line represents the gray matter–white matter border. Scale bars: (A and B) = 200 μm, (D) = 50 μm. C-LTMR, C–low-threshold mechanoreceptor; GRP, gastrin-releasing peptide.

During the course of experiments with the GRP::eGFP mouse, we noted that there was some variability in the number of GFP^+^ cells in the superficial dorsal horn in different mice. In addition, we observed a consistently lower number of GFP-positive cells in the medial part of the dorsal horn in caudal lumbar segments (especially L4 and L5) (Figs. 1A and B). Because this region is innervated by glabrous skin of the hind paw, we compared the proportion of neurons that were GFP^+^ in regions innervated by hairy and glabrous skin. This analysis was performed in the L4 segment (which receives input from both hairy and glabrous skin) and L1, which receives its cutaneous input only from hairy skin. The mean numbers of neurons counted per segment were 934 (882-1122) for L4 and 738 (702-808) for L1 (n = 3 mice). Although the percentage of laminae I and II neurons that were GFP^+^ in L1 and in the lateral (hairy skin) part of L4 was very similar (12%-14%), only 3% of those in the medial (glabrous skin) part of L4 expressed GFP (Fig. 1C). A 2-way repeated-measures ANOVA demonstrated a significant effect of lumbar location (F(1, 2) = 37.15, *P* = 0.026) and mediolateral location (F(1, 2) = 27.3, *P* = 0.035). The interaction between these factors was significant (F(1, 2) = 86.61, *P* = 0.011), and the *post hoc* Sidak test shows a significant mediolateral difference at L4 (*t*(2) = 2.08, *P* = 0.0085) but not at L1 (*t*(2) = 15.24, *P* = 0.32).

The lack of GRP::eGFP cells in medial L4 and L5 could reflect the absence of cells that express GRP or a lack of GFP in some GRP-expressing neurons in the BAC transgenic line. To distinguish between these possibilities, we performed in situ hybridisation histochemistry on tissue from GRP::eGFP mice. We identified a mean of 43.7 (35-54) GFP^+^ cells and 96.0 (90-107) GRP mRNA cells in sections from 3 mice and found that virtually all (98%) GFP^+^ cells had detectable GRP mRNA, whereas 44.5% (39%-51%) of cells with the mRNA were GFP-positive. Gastrin-releasing peptide mRNA^+^ cells were found throughout the mediolateral extent of the dorsal horn in the midlumbar region (Fig. 1D) and the medial part of the L4 and L5 segments contained numerous GRP mRNA^+^/GFP-negative cells. We could not determine the proportion of neurons with GRP mRNA, due to the lack of a neuronal marker in these sections, but we noted that although the lateral and medial parts (defined by the presence and absence of the intense GFP plexus) were approximately equal in size, GRP mRNA cells were more numerous in the lateral part (57-84 cells, mean 65.3 for the lateral part; 23-36 cells, mean 30.6 for the medial part; n = 3 mice). However, the extent of overlap between GRP mRNA and GFP was significantly different, depending on mediolateral location. In the medial part, only 30.5% (28%-33%) of GRP mRNA-positive cells were GFP-positive, compared with 50.8% (46%-56%) in the lateral part, and this difference was significant (*t* test, *t*(4) = 5.93, *P* = 0.004). We may have underestimated the proportion of GRP mRNA cells that express GFP due to some loss of GFP signal resulting from the in situ hybridisation protocol.^57^ However, these results suggest that although there may be fewer GRP mRNA-positive cells in the glabrous skin territory, lack of expression of GFP in GRP-expressing neurons is partially responsible for the low numbers of GFP cells seen in this region in the GRP::eGFP mouse.

Because we do not have a convenient way of identifying cells with GRP mRNA that did not express GFP, our subsequent analysis was restricted to the GFP-positive population. It should be borne in mind that these cells may differ functionally from the GFP-negative cells that contain GRP mRNA.

### 3.2. Limited coexpression of gastrin-releasing peptide and substance P

**Figure 2.**
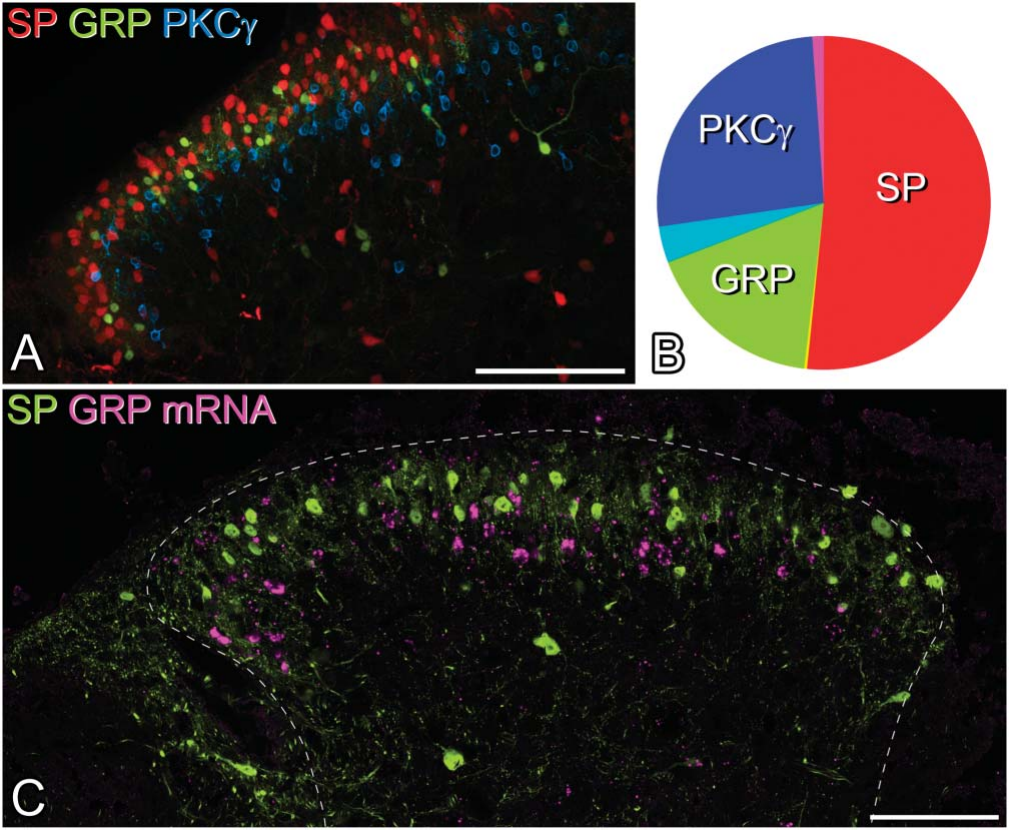
Distribution of cells expressing substance P (SP), GRP, and PKCγ. (A) A confocal image showing part of a transverse section through the dorsal horn of the L3 segment from a Tac1^Cre^;GRP::eGFP mouse that had received an intraspinal injection of AAV.flex.tdTom 2 weeks previously. The section has been immunostained for GFP (green) and PKCγ (blue), while the native fluorescence of tdTom is shown in red. Cells containing each of these markers form largely separate populations. Although these overlap, the tdTom^+^ (SP-expressing) cells are generally dorsal, and the PKCγ cells ventral, to the GRP-eGFP cells. (B) Pie chart showing the relative sizes and extent of overlap of these populations. (C) Lack of coexpression of GRP and SP is further supported by the finding that there is virtually no overlap between GRP mRNA (magenta) and GFP (green) in the L3 segment of a Tac1^Cre^ mouse that had received an intraspinal injection of AAV.flex.eGFP. Scale bars: (A and B) = 100 μm. AAV, adeno-associated virus; GRP, gastrin-releasing peptide.

We previously identified SP-expressing neurons by using immunocytochemistry to reveal PPTA and reported that there was minimal overlap between PPTA-immunoreactive neurons and those that were GFP-positive in the GRP::eGFP mouse.^23^ However, we also found that the PPTA antibody labelled fewer neurons than were seen after intraspinal injection of AAV.flex.tdTom into Tac1^Cre^ mice. It is therefore possible that we underestimated the degree of overlap between these populations. To better separate these populations, we performed experiments on Tac1^Cre^;GRP::eGFP and Tac1^Cre^ mice that had received intraspinal injections of AAVs coding for either tdTom or eGFP (Fig. 2). We first examined tissue from 2 Tac1^Cre^;GRP::eGFP mice that had been injected with AAV.flex.tdTom, and measured the extent of overlap between tdTom^+^ (SP cells) and GFP^+^ (GRP cells) in laminae I and II. We also stained for PKCγ, which is found in a different population of excitatory interneurons.^26,57^ This analysis provided further evidence that the SP, GRP, and PKCγ cells are largely separate populations (Figs. 2A and B). The mean number of cells that were tdTom^+^, GFP^+^, and/or PKCγ^+^ in sections from the 2 mice was 356 (364 and 347 in the 2 mice). Of these, 189 (199, 178) were tdTom-positive, 75 (80, 70) were GFP-positive, and 110 (114, 105) were PKCγ-immunoreactive. As reported previously,^24,26^ we found some overlap between GRP-eGFP cells and PKCγ-immunoreactive cells (corresponding to 17% of the GFP cells and 11% of those with PKCγ), but minimal overlap between tdTom and PKCγ cells (corresponding to 2.1% of the tdTom cells and 3.6% of those with PKCγ). In addition, we saw very little overlap of the tdTom (SP) population with the GRP-GFP cells (1.3% of the GRP-GFP cells and 0.6% of the tdTom cells).

Because some GRP cells may not be detected in the GRP::eGFP mouse, we also compared the distribution of GRP mRNA with that of GFP in 3 Tac1^Cre^ mice that had been injected with AAV.flex.eGFP (Fig. 2C). We identified a mean of 75.3 (68-85) GFP^+^ cells and 46.3 (44-49) GRP mRNA cells and found only 2.3 (2-3) double-labelled cells (corresponding to 3.2% of the GFP population and 5.2% of the GRP mRNA cells).

**Figure 3.**
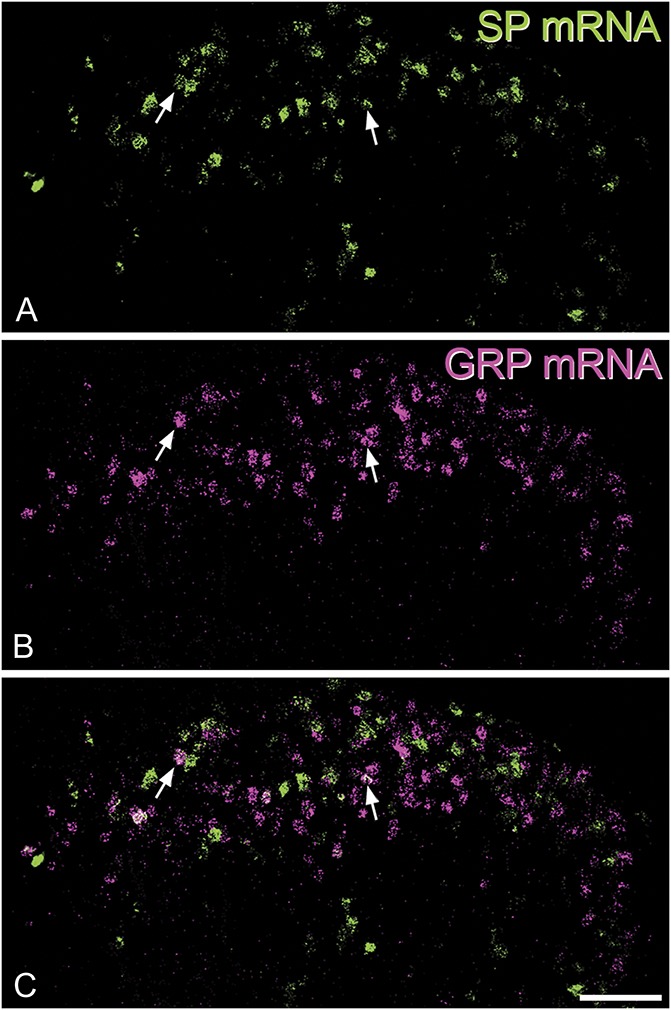
Limited coexistence of mRNAs for SP and GRP in a section of the cervical dorsal horn that had been reacted with a double-label in situ hybridisation method. (A) Substance P mRNA is shown in green. (B) The same field scanned to reveal GRP mRNA (magenta). (C) A merged image shows that most cells that are labelled contain only one of the mRNA types. However, some cells are double-labelled, and 2 of these are indicated with arrows. Scale bar = 50 μm. GRP, gastrin-releasing peptide; SP, substance P.

In sections of the cervical cord that had undergone double-labelling in situ hybridisation (Fig. 3), we identified a mean of 678 cells with SP mRNA (range 561-783, n = 3 mice) and 618 (483-715) cells with GRP mRNA. The mean number of cells in these sections that contained both SP and GRP mRNAs was 72 (range 54-85), and this corresponded to 10.7% (9.6%-11.3%) of the cells with SP mRNA and 11.7% (10.9%-12.9%) of those with GRP mRNA. A similar pattern was seen in the sections of the lumbar cord (1 mouse): we identified 572 cells with SP mRNA, 497 cells with GRP mRNA, and 60 of these cells had both mRNAs. In this case, cells with both mRNAs corresponded to 10.5% of the GRP^+^ cells and 12.2% of the SP^+^ cells. We conclude that although there is a limited overlap between SP and GRP cells, they are largely separate populations.

### 3.3. Electrophysiological properties of gastrin-releasing peptide and substance P cells

As we did not observe any differences between male and female mice, all data presented are from a combination of both sexes. For the electrophysiological parts of the study, GRP cells were identified by GFP expression in slices from the GRP::eGFP mice, whereas SP cells were identified by expression of either GFP or tdTom in slices obtained from Tac1^Cre^ mice that had received intraspinal injections of AAVs coding for Cre-dependent forms of the corresponding fluorescent protein. Because GRP-eGFP cells were relatively infrequent in the medial part of the L4 and L5 segments, parasagittal slices through these segments were cut in such a way as to allow recordings from cells located in the more lateral parts of the segment.

**Figure 4.**
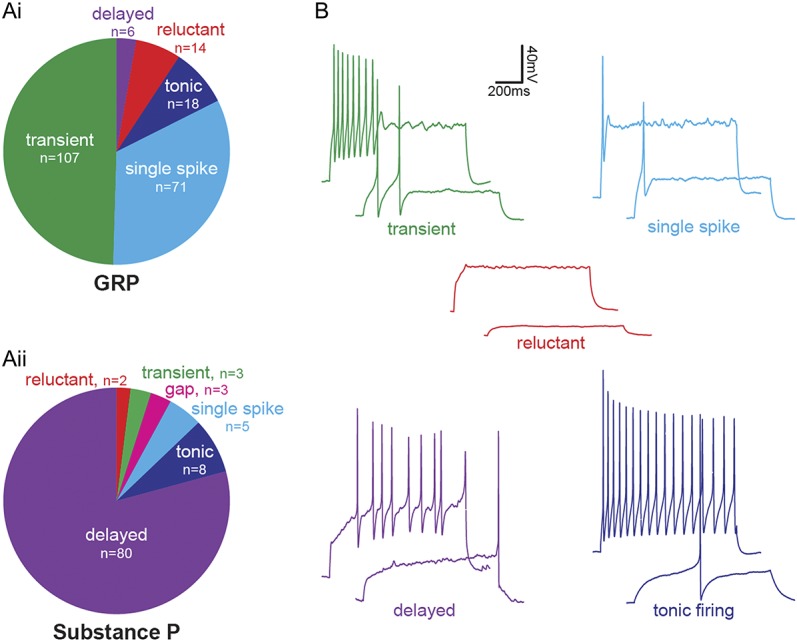
Action potential firing patterns in GRP and SP cells. GRP cells were identified by the presence of GFP in slices taken from GRP::eGFP mice. Substance P cells were identified by expression of either GFP or tdTom in Tac1^Cre^ mice that had received injections of AAV coding for Cre-dependent forms of the corresponding fluorescent protein. (Ai) In response to a suprathreshold current injection (1 second), most GRP cells displayed transient (107/216, 49.5%) or single-spike (71/216, 32.9%) firing, with a smaller proportion of cells showing tonic (18/216, 8.3%), reluctant (14/216, 6.5%), or delayed (6/216, 2.8%) firing. (Aii) The majority of SP cells exhibited delayed firing (80/101, 79.2%), with smaller proportions displaying tonic (8/101, 7.9%), single-spike (5/101, 5%), transient (3/101, 3%), gap (3/101, 3%), or reluctant (2/101, 2%) firing patterns. (B) Examples of transient, single-spike, and reluctant firing GRP cells, and delayed and tonic-firing SP cells. AAV, adeno-associated virus; GRP, gastrin-releasing peptide; SP, substance P.

The incidence of action potential firing patterns differed substantially between GRP and SP cells (Fig. 4). Gastrin-releasing peptide cells generally displayed transient (107/216, 49.5%) or single-spike (71/216, 32.9%) firing patterns, which were rarely seen in SP cells (3/101, 3% and 5/101, 5%, respectively). By contrast, the great majority of SP cells (80/101, 79.2%) exhibited the delayed firing pattern, which was very seldom seen in the GRP cells (6/216, 2.8%). Both cell populations contained a small proportion of tonic-firing cells (GRP: 18/216, 8.3% and SP: 8/101, 7.9%), together with a few cells that were classified as reluctant (GRP: 14/216, 6.5% and SP: 2/101, 2%), while 3 of the SP cells (3.0%) showed a long first interspike interval and were classified as gap-firing.^29^

We have shown that when Tac1^Cre^ mice received intraspinal injections of AAV.flex.eGFP, around 10% of the GFP^+^ cells are Pax2^+^ (inhibitory) neurons, and these are therefore likely to have been included among the neurons recorded in these experiments. Previous studies have shown that delayed, gap, and reluctant firing patterns, which are believed to result from the presence of A-type potassium currents, are particularly associated with excitatory interneurons in lamina II.^29,49,73^ For all subsequent electrophysiological and pharmacological parts of the study, we therefore restricted the analysis to the 84% of SP cells (85/101) that showed delayed, gap, or reluctant firing. This approach reduced the risk that recordings were from inhibitory SP neurons.

Consistent with the much higher proportion of SP cells that showed gap or delayed firing, the latency between the onset of the current injection and the first action potential at rheobase was significantly greater for the SP cells (595.0 ± 28.26 ms) compared with the GRP cells (137.1 ± 6.2 ms) (Mann–Whitney, *U* = 504, *P* < 0.001, n = 155 GRP cells and 80 SP cells). The rheobase of SP cells was 46.45 ± 2.15 pA, and this was significantly larger than that of GRP cells, 18.30 ± 1.07 pA (Mann–Whitney, *U* = 1275, *P* < 0.001, n = 155 GRP cells and 80 SP cells).

The subthreshold I–V relationship was determined by voltage clamping the cells at −60 mV and applying 100-millisecond voltage steps from −70 to −50 mV in 2.5 mV increments. The resting membrane potential, as calculated from the I–V relationship for individual cells, was −52.89 ± 0.78 mV for GRP cells and −55.77 ± 0.90 mV for SP cells, with the GRP cells having a significantly more depolarised resting membrane potential (Mann–Whitney, *U* = 7758, *P* = 0.0074, n = 230 GRP cells and 84 SP cells). The capacitance of GRP cells was significantly smaller than that of SP cells (5.12 ± 0.11 vs 7.07 ± 0.33 pF; Mann–Whitney, *U* = 5904, *P* < 0.001, n = 232 GRP cells and 85 SP cells), with GRP cells also displaying a greater input resistance (1588 ± 85 vs 836 ± 52 MΩ; Mann–Whitney, *U* = 5315, *P* < 0.001, n = 232 GRP cells and 84 SP cells).

**Figure 5.**
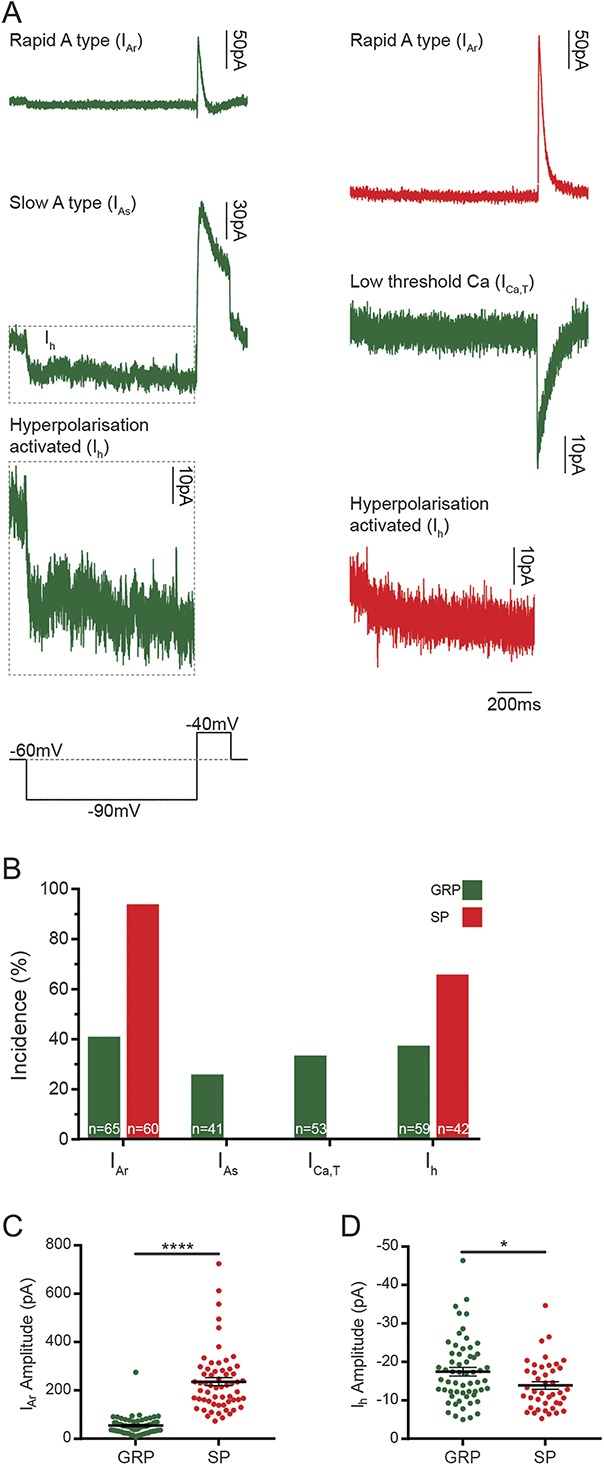
Subthreshold voltage-activated currents in GRP and SP cells, which were identified as described in the legend for Figure 4. (A) Subthreshold currents were examined in GRP and SP cells using a voltage step protocol that hyperpolarised cells from −60 to −90 mV for 1 second and then to −40 mV for 200 milliseconds (lower left trace). The responses to this protocol were classified as rapid (I_Ar_) or slow (I_As_) A-type potassium currents, hyperpolarisation-activated currents (I_h_), or low-threshold calcium currents (I_Ca,T_). Examples from both GRP (green) and SP (red) cells show an average of 5 traces. The example of I_h_ for the GRP cell (dashed outline) is shown at 2 different y-axis scales. (B) Almost all SP cells displayed I_Ar_ (60/64, 93.8%) and most had I_h_ (42/64, 65.6%), with many cells exhibiting both (38/64, 59.4%). I_Ar_ was the most commonly seen current in GRP cells (65/159, 40.9%), with fewer cells showing I_h_ (59/159, 37.3%), I_As_ (41/159, 25.8%), or I_Ca,T_ (53/159, 33.3%). (C) The amplitude of I_Ar_ was significantly greater in SP cells (C, 263.3 ± 16.5 vs 58.6 ± 4.6 pA), ****P < 0.0001, Mann–Whitney *U* test. (D) I_h_ amplitude was significantly larger in GRP cells (D, −17.5 ± 1.1 vs −13.9 ± 1.0 pA). *P = 0.024, Mann–Whitney *U* test. GRP, gastrin-releasing peptide; SP, substance P.

Almost all SP neurons tested (60/64, 93.8%, Figs. 5A and B) displayed a rapid I_A_ current (I_Ar_), and most showed a hyperpolarisation-activated current (I_h_) (42/64, 65.6%), with many exhibiting both I_Ar_ and I_h_ (38/64, 59.4%). Although I_Ar_ was the most commonly seen subthreshold current in GRP cells (65/159, 40.9%, Figs. 5A and B), the incidence was lower than that in SP cells, and the amplitude was significantly smaller (54.9 ± 4.6 vs 263.3 ± 16.5 pA, Mann–Whitney, *U* = 64, *P* < 0.0001, Fig. 5C). Many GRP cells displayed I_h_ (59/159, 37.3%), with some cells exhibiting both I_h_ and I_Ar_ (13/159, 8.2%). The amplitude of I_h_ in GRP cells was significantly larger than that recorded in SP cells (−17.5 ± 1.1 vs −13.9 ± 1.0 pA, Mann–Whitney, *U* = 911.5, *P* = 0.024, Fig. 5D). Although not detected in the SP cells, slow I_A_ current (I_As_) (41/159, 25.8%) and low-threshold Ca currents (I_Ca,T_) (53/159, 33.3%) were recorded in some GRP cells. Some GRP cells that showed I_As_ also exhibited I_h_ (36/159, 22.6%) or I_h_ and I_Ca,T_ (1/159, 0.6%), and I_Ca,T_ was found to overlap with I_Ar_ (5/159, 3.1%) and with I_h_ (3/159, 1.9%).

### 3.4. Excitatory inputs to gastrin-releasing peptide and substance P cells

**Figure 6.**
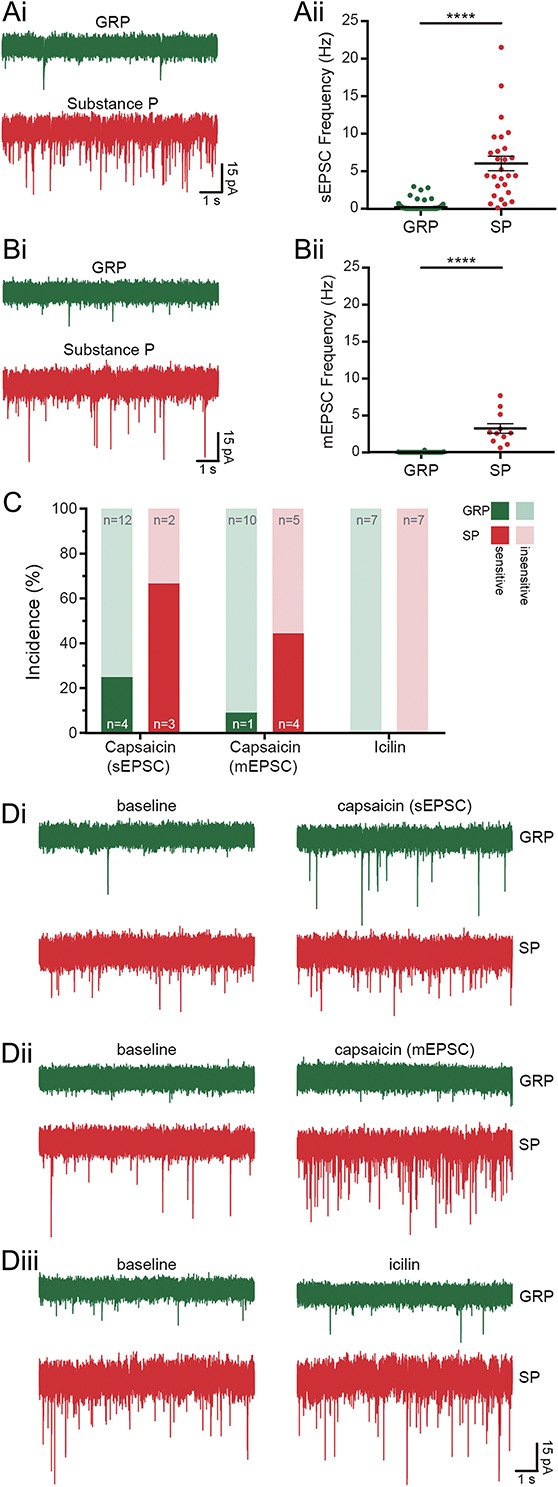
Excitatory inputs to GRP and SP cells, which were identified as described in the legend for Figure 4. Spontaneous EPSCs (Ai) and mEPSCs (Bi) were recorded in GRP and SP cells. Substance P cells were found to receive greater excitatory drive, having a higher frequency of both types of excitatory event (6.04 ± 0.96 Hz sEPSCs, Aii, 3.24 ± 0.67 Hz mEPSCs, Bii, n = 27 and 11, respectively) than GRP cells (0.2 ± 0.05 Hz sEPSCs, Aii, 0.02 ± 0.01 Hz mEPSCs, Bii, n = 120 and 32, respectively). These differences were both highly significant (****P < 0.0001, Mann–Whitney *U* test). (C and Di-iii) Primary afferent input to GRP and SP cells was assessed by recording sEPSCs and mEPSCs in response to the TRP channel agonists capsaicin (TRPV1, Di–ii) and icilin (TRPM8, mEPSCs only, Diii). (C) When mEPSCs were recorded, capsaicin caused a significant leftwards shift in the distribution of interevent intervals in 1 of 11 GRP cells and 4 of 9 SP cells; when sEPSCs were recorded, 4 of 16 GRP cells and 3 of 5 SP cells were found to be capsaicin-sensitive. Icilin did not increase mEPSC frequency in any of the GRP (n = 7) or SP cells (n = 7) tested. GRP, gastrin-releasing peptide; mEPSC, miniature excitatory postsynaptic current; sEPSC, spontaneous excitatory postsynaptic current; SP, substance P.

As stated above, analysis of SP cells was restricted to those with delayed, gap, or reluctant firing patterns. Substance P cells showed a higher frequency of both sEPSCs and mEPSCs (6.04 ± 0.96 Hz sEPSCs, 3.24 ± 0.67 Hz mEPSCs, n = 27 and 11, respectively) than GRP cells (0.2 ± 0.05 Hz sEPSCs, 0.02 ± 0.01 Hz mEPSCs, n = 120 and 32, respectively), and these differences were both highly significant (sEPSC, Mann–Whitney, *U* = 68, *P* < 0.0001; mEPSC, Mann–Whitney, *U* = 0, *P* < 0.0001; Fig. 6). For 11 of the SP cells, we were able to compare sEPSC frequency with mEPSC frequency in the same cell. The sEPSC frequency for these cells (5.39 ± 0.93 Hz) was higher than the mEPSC frequency (3.24 ± 0.67 Hz), and this difference was highly significant (Wilcoxon signed-rank test; W = 66, *P* = 0.0036). This finding suggests that the SP cells receive excitatory synaptic input from neurons that were spontaneously firing action potentials in the slice.

Capsaicin caused a leftwards shift in the distribution of mEPSC interevent intervals in 1 of 11 GRP and 4 of 9 SP cells (Figs. 6C and D). Capsaicin increased mEPSC frequency from 0.04 to 0.17 Hz in the single responsive GRP cell, and from 3.58 ± 0.91 to 8.78 ± 1.79 Hz in the responsive SP cells (example traces shown in Fig. 6Dii). When sEPSCs were recorded, 4 of 16 GRP and 3 of 5 SP cells were found to be sensitive to capsaicin (Fig. 6C). For those cells that were classed as sensitive, capsaicin increased sEPSC frequency from 0.36 ± 0.10 to 3.58 ± 2.09 Hz in GRP cells, and 1.64 ± 0.16 to 3.29 ± 0.30 Hz in SP cells (example traces shown in Fig. 6Di). Application of icilin did not increase mEPSC frequency in any of the GRP (n = 7) or SP cells (n = 7) that were tested (Figs. 6C and Diii). Because TRPM8 and TRPV1 expression in the dorsal horn are believed to be restricted to primary afferents, these data suggest that neither GRP nor SP cells receive monosynaptic input from TRPM8-expressing afferents. These findings also indicate that both cell types receive input from TRPV1-expressing afferents, and that this includes monosynaptic input, although this is considerably more prevalent in SP cells than GRP cells.

### 3.5. Responses of gastrin-releasing peptide and substance P cells to neuromodulators

**Figure 7.**
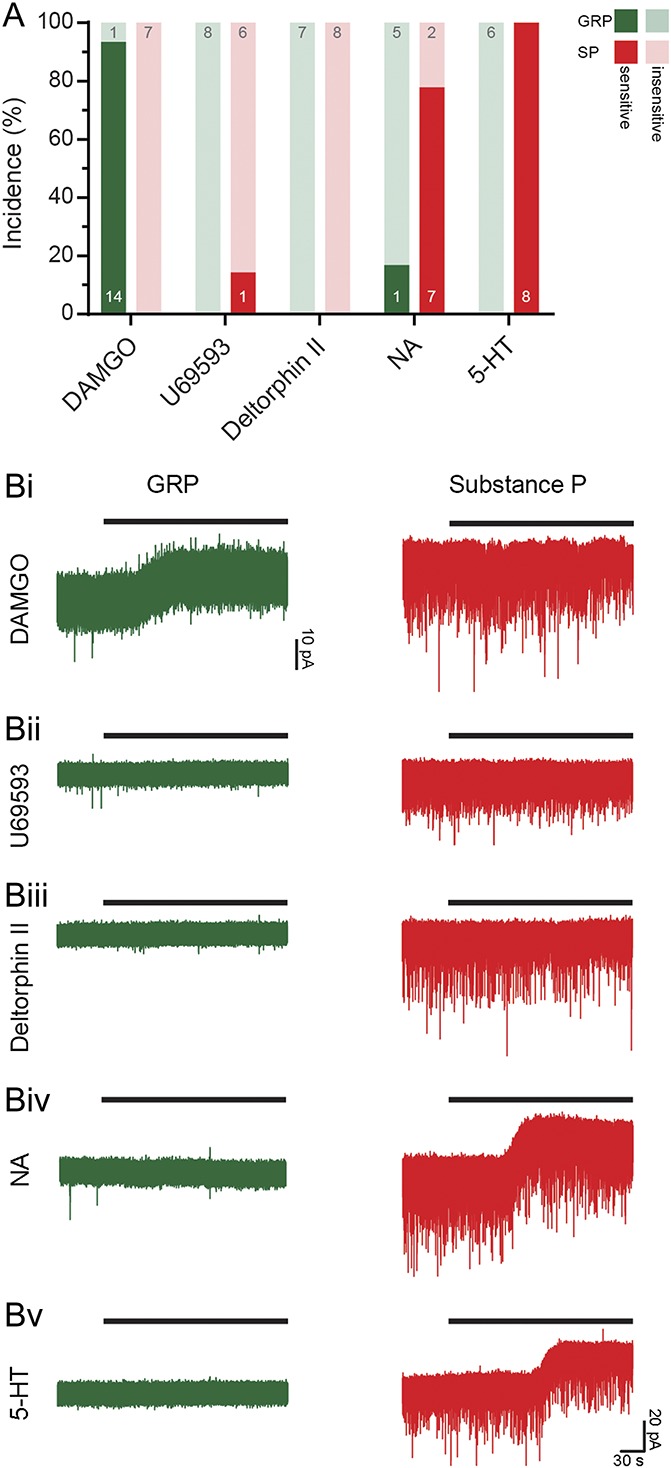
Responses of GRP and SP cells to neuromodulators. Cells were identified as described in the legend for Figure 4. (A) The proportions of tested GRP and SP cells that responded to various neuromodulators are shown. (Bi) The MOR agonist DAMGO caused an outward current in most GRP cells (14/15) but was without effect in SP cells. (Bii) No GRP cells and only 1 of 7 SP cells responded to the KOR agonist, U69593. (Biii) No cells of either type responded to the DOR agonist, [D-Ala^2^]-Deltorphin II. (Biv) Noradrenaline (NA) elicited a response in most SP cells (7/9), but only 1 of 6 GRP cells. (Bv) 5-HT evoked an outward current in all 8 SP cells, whereas the 6 GRP cells tested were unresponsive. GRP, gastrin-releasing peptide; SP, substance P.

To investigate the effect of neuromodulators (NA, 5-HT, and opioids) on GRP and SP cells, we bath-applied different agonists (Fig. 7). DAMGO (3 μM) caused an outward current (7.36 ± 0.99 pA) in all but one of the GRP cells tested (14/15); none of the 7 SP cells tested were responsive. None of the GRP cells responded to the KOR agonist, U69593 (1 μM), and this caused an outward current (7.23 pA) in only 1 of the 8 SP cells tested. The DOR agonist [D-Ala^2^]-Deltorphin II (1 μM) was tested on 7 GRP cells and 8 SP cells, but had no effect on any of these. Application of NA (20 μM) caused an outward current in the majority (7 of 9) of the SP cells (15.59 ± 2.54 pA), but in only 1 of 6 GRP cells (8.86 pA). None of the 6 GRP cells tested responded to 5-HT (10 or 20 μM), whereas all 8 SP cells tested displayed an outward current (19.22 ± 2.55 pA). These findings demonstrate that GRP and SP cells differ in their response profiles to the monoamines and MOR agonist, whereas few or none of these cells respond to KOR or DOR agonists. Specifically, most putative excitatory SP cells are hyperpolarised by both NA and 5-HT, but not by any of the opioid agonists. By contrast, most GRP cells do not respond to NA, 5-HT, DOR, or KOR agonists, but are hyperpolarised by MOR agonists.

### 3.6. Morphological properties of gastrin-releasing peptide and substance P cells

**Figure 8.**
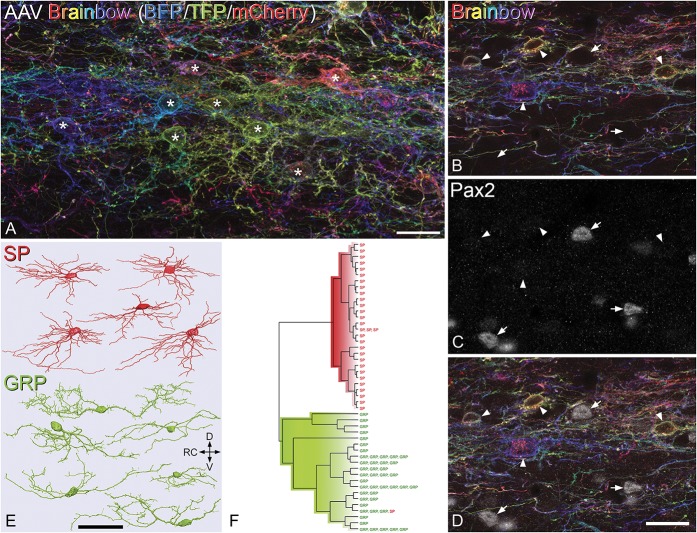
Morphology of SP and GRP cells. (A) The labelling that results from injection of AAV Brainbow into the dorsal horn of a Tac1^Cre^ mouse. This field shows part of lamina II in a projected image of 38 optical sections at 0.5-μm z-separation. Numerous neuronal cell bodies are visible (some marked with asterisks), and these show different hues, resulting from differential expression of blue fluorescent protein (BFP), teal fluorescent protein (TFP), and mCherry. Note that apart from some cytoplasmic staining for mCherry, the labeling is largely restricted to the plasma membrane and therefore outlines the cells. (B–D) A projection of 5 optical sections at 1-μm z-separation from a Tac1^Cre^ mouse injected with AAV Brainbow. This has been scanned to reveal the fluorescent proteins (shown in B and D) and Pax2 (C and D). Although there are Pax2-positive cells in this field (some marked with arrows), none of these correspond to the Brainbow-labelled cells (arrowheads). (E) Neurolucida reconstructions of the cell bodies and dendritic trees of representative SP and GRP cells. Note that the SP cells were obtained from Brainbow experiments, whereas the GRP cells were from electrophysiological recordings in the GRP::eGFP mice. (F) Hierarchical cluster analysis (Ward's method) based on morphometric dendritic parameters results in almost complete separation of the SP and GRP cells, with only 1 of 31 SP cells appearing in the lower cluster, which contains all 45 of the GRP cells. Scale bars: (A) = 20 μm, (B–D) = 20 μm, and (E) = 50 μm. AAV, adeno-associated virus; D, dorsal; GRP, gastrin-releasing peptide; RC, rostrocaudal; SP, substance P; V, ventral.

Morphological analysis was performed on 45 GRP cells that underwent whole-cell recording, and from a total of 43 SP cells (31 from perfusion-fixed Brainbow tissue and 12 from electrophysiological experiments). In the tissue from Tac1^Cre^ mice injected with Brainbow AAVs, initial scans revealed that the distribution and density of labelled cells was generally consistent with that seen after injection of AAV.flex.tdTom^23^ (Fig. 8A). However, although ∼10% of the labelled cells in our previous study were Pax2-immunoreactive, only 1 of 100 Brainbow-labelled neurons examined was Pax2-positive, although numerous Pax2^+^ nuclei were present within the dorsal horn (Figs. 8B–D). Clearly, this strategy selectively targets the excitatory SP-expressing neurons. All 31 reconstructed SP neurons had nuclei that were Pax2-negative.

Preliminary observation of the Neurolucida reconstructions suggested that the 2 populations differed in terms of somatodendritic morphology. Although the GRP cells were morphologically heterogeneous, they generally had dendritic trees that were considerably longer in the RC axis than in DV or mediolateral axes, but were shorter than those of islet cells. At least some of these cells could therefore be classed as central cells.^22,71,73^ By contrast, Figure 8E shows that many of the SP cells reconstructed from the Brainbow experiments resembled radial cells, with relatively numerous primary dendrites that did not extend far from the cell body, and compact dendritic trees. This initial observation was confirmed by principal component analysis and subsequent cluster analysis of dendritic morphometric parameters extracted from the Neurolucida drawings. These parameters are listed in detail in Table 5 of Ref. 18. A scree test revealed that 5 principal components accounted for 80% of the total variance in the data set, and these were therefore used for cluster analysis. This separated the reconstructed neurons into 2 distinct clusters, one of which (n = 30) consisted entirely of SP cells, and the other (n = 46) of which included all the GRP cells, together with one SP cell (Fig. 8F).

**Figure 9.**
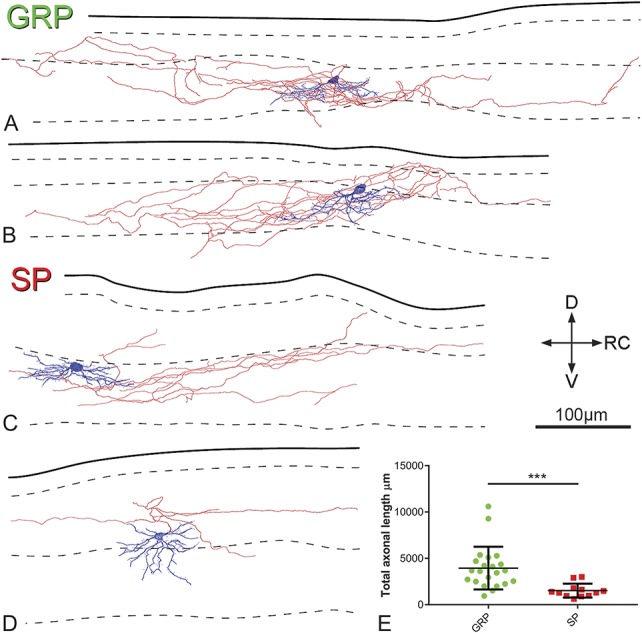
Morphology and laminar distribution of axons of GRP and SP cells. Cells were reconstructed after electrophysiological recordings, during which they were identified as described in the legend for Figure 4. (A–D) Typical examples of Neurolucida reconstructions of GRP cells (A and B) and SP cells (C and D). Cell bodies and dendrites are shown in blue and axons in red (axonal boutons are not shown). In each drawing, the solid line indicates the gray-white border, whereas dashed lines represent the boundaries between laminae I/IIo, IIo/IIi, and IIi/III. Note that the axons of the cells predominantly arborise in lamina II and rarely enter lamina I. Scale bar = 100 μm. The total measured axonal length in each population is shown (E) with the total length of axon being significantly greater in the GRP cells . *** Denotes significant difference (*P*<0.001, *t*-test). D, dorsal; GRP, gastrin-releasing peptide; RC, rostrocaudal; SP, substance P; V, ventral.

Because the 2 populations were obtained from different types of experiment, we were concerned that this might have influenced the clustering results. For example, due to the difficulty of following fine distal dendrites, we may have underestimated the sizes of dendritic trees in the Brainbow material. We therefore looked for factors that correlated well with the principal components distinguishing the clusters and found that the number of primary dendrites was a major factor. We therefore compared the number of primary dendrites between the 2 populations and found a highly significant difference (Table 3). We also found that both the DV extent of dendritic trees and the ratio of DV to RC extent^71^ differed significantly. The SP cells had more primary dendrites and a lower RC:DV ratio (Table 3), both of which are consistent with radial cell morphology. Although we did not analyse the dendritic trees of the recorded SP cells in detail, we noted that these resembled those of the SP neurons seen in the Brainbow tissue (Figs. 9C and D). These cells also gave rise to numerous primary dendrites (mean 6.9 ± 1.5 SD), which is similar to the number of primary dendrites on the reconstructed Brainbow neurons (7.4 ± 1.4, Table 3).

**Table 3:**
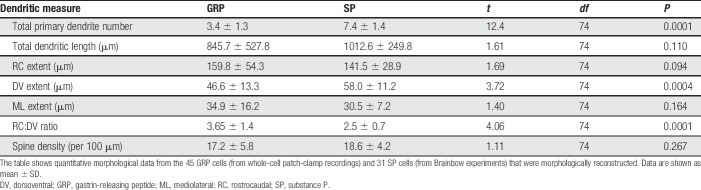
Morphological properties of dendrites of GRP and SP cells.

Because of the difficulty of following axons belonging to individual neurons in Brainbow material, axonal morphology for the SP cells was only analysed on those that had undergone whole-cell recording. For many of the GRP cells, we found that although a well-filled axon could be seen emerging from the soma or a primary dendrite, the axon rapidly turned either medially or laterally and left the slice either without branching (n = 14) or after giving rise to a small arbor (n = 9). For this reason, axonal morphology was only analysed on 22 of the 45 GRP cells. Examples of axonal arbors are illustrated in Figure 9. The total length of axon that was reconstructed was significantly greater for the GRP than for the SP cells (*t* test, *t*(32) = 3.68, *P* = 0.0008; Fig 9E). However, in both cases, 95% of the axonal length remained in lamina II, with only a small amount in laminae I or III (2% and 4%, respectively, for GRP cells, 4% and 1%, respectively, for SP cells).

### 3.7. Lack of long propriospinal projections of gastrin-releasing peptide cells

**Figure 10.**
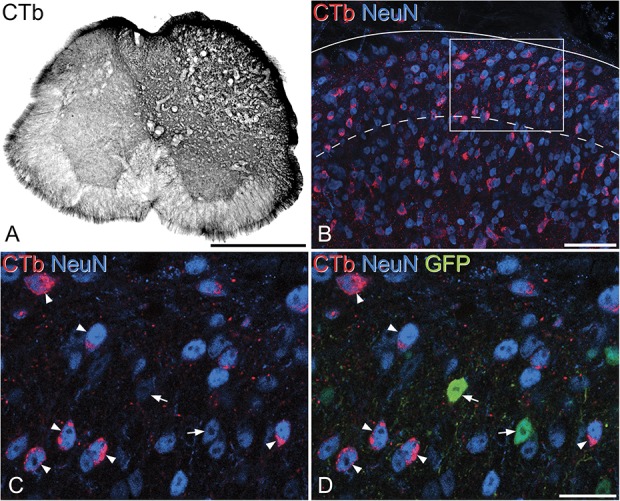
Retrograde labelling of L5 neurons from the T13 segment in a GRP::eGFP mouse. (A) The injection site in the T13 segment of one of the mice. The CTb reaction product fills the entire dorsal horn and part of the ventral horn on the right side, with limited spread onto the contralateral side. (B) A Projected confocal image (17 optical sections at 2-μm z-spacing) showing part of the ipsilateral dorsal horn from the L5 segment of the same animal. Numerous NeuN^+^ (neuronal) profiles are visible (blue), and many of these are labelled with CTb (red) that has been retrogradely transported from the T13 injection site. The solid line indicates the dorsal edge of the dorsal horn and the dashed line the lamina II-III border. (C and D) A single confocal optical section showing a higher magnification view from the region in the box in (B). This field contains 2 GFP^+^ (green) cells (arrows), which are not CTb-labelled, and these are surrounded by several GFP-negative CTb-labelled neurons, some of which are marked with arrowheads. Scale bars: (A) 500 μm; (B) 50 μm; and (C and D) 20 μm. CTb, cholera toxin B; GRP, gastrin-releasing peptide.

The finding that the axons of recorded GRP cells often turned medially or laterally raised the possibility that these had propriospinal projections,^7,25^ and we therefore addressed this in the retrograde labelling experiments. The CTb injection sites in the T13 segment in these experiments included the whole of laminae I to V of the right dorsal horn as well as the lateral spinal nucleus (LSN) (Fig. 10A). The pattern of retrograde labelling with CTb in the L5 segment was very similar to that described previously.^25^ There were numerous CTb-labelled neurons evenly distributed throughout the mediolateral extent of the superficial dorsal horn, as well as in deeper laminae (Fig. 10B). Few retrogradely labelled cells were seen on the contralateral (left) side. The mean number of laminae I and II neurons included in the disector sample in the 4 mice was 589 (range 547-620), and 26.6% were CTb-immunoreactive (range 22.1%-29.6%), indicating that at least a quarter of superficial dorsal horn neurons in L5 have axons that extend rostrally for 5 segments.

Cells that were positive for eGFP in the L5 segment accounted for 10.3% (7.6%-12.6%) of all laminae I and II neurons, which is similar to our previous estimate (11%).^24^ Very few of the GFP^+^ cells, 3.5% (2.6%-4.7%), were CTb-immunoreactive, while GFP-immunoreactive cells accounted for only 1.3% (1.1%-1.5%) of the CTb-labelled neurons in laminae I and II (Figs. 10C and D). This difference in proportion was highly significant (Mantel–Haenszel test, χ^2^(1) = 73.0, *P* < 0.0001), with an odds ratio for a GRP cell to be retrogradely labelled calculated as 0.08 (0.04-0.17; 95% confidence interval, Breslow–Day significance; *P* = 0.944). We conclude that GRP-expressing cells are significantly underrepresented among the superficial dorsal horn neurons that have long ascending propriospinal axons.

To test whether GRP cells give rise to short intersegmental connections, we also analysed sections from the L2 segment in 2 of the mice. We found that the proportion of all neurons in laminae I and II that were retrogradely labelled with CTb from the T13 injection sites was 58% (range 55.9%-60.1%), and that among GRP-eGFP cells, the proportion that were CTb-labelled was 15.1% (range 13%-17.1%). This indicates that a few of the GRP cells have axons that extend for at least 2 segments rostrally.

### 3.8. Responses of gastrin-releasing peptide cells to noxious and pruritic stimuli

Noxious and pruritic stimuli induced pERK in cells of the ipsilateral dorsal horn, mainly in the superficial laminae with a mediolateral extent that reflected the somatotopic location of a stimulus delivered to the lateral calf.^6,23^ Few, if any, pERK cells were located on the contralateral side or in the dorsal horn of mice that received vehicle injection.

**Figure 11.**
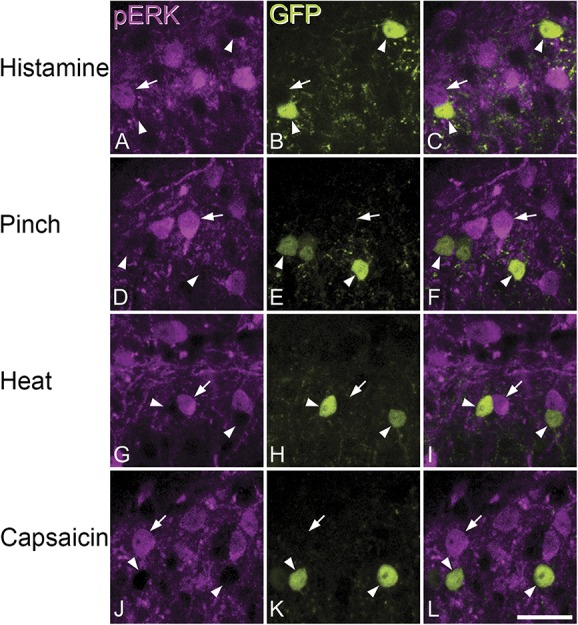
Phosphorylated extracellular signal–regulated kinase immunoreactivity in GRP::eGFP mice after noxious or pruritic stimulation. (A–C, D–F, G–I, and J–L) Representative fields from mice that were stimulated with histamine, pinch, noxious heat, or capsaicin, respectively. In each case, the same field from the superficial dorsal horn is shown immunostained for pERK (magenta), GFP (green), together with a merged image. Each stimulus resulted in numerous pERK^+^ neurons, although GFP-labelled cells were rarely pERK-immunoreactive. Each set of images shows GFP^+^ cells (some indicated with arrowheads), which are not immunostained for pERK, but are surrounded by pERK^+^ neurons. In each case, one of the pERK^+^ neurons is indicated with an arrow. Images are projections of 2 (A–C and J–L) or 3 (D–I) confocal optical sections at 2-μm z-spacing. Scale bar = 20 μm. GRP, gastrin-releasing peptide; pERK, phosphorylated extracellular signal–regulated kinase.

First, we determined the proportion of all neurons that were pERK-positive after stimulation with histamine, capsaicin, pinch, and noxious heat. The percentage of all neurons within the activated zone in laminae I and II that were pERK-immunoreactive varied between 23% and 37% depending on the stimulus (Table 4). We then compared the proportions of GFP^+^ and GFP-negative cells that showed pERK after the different types of noxious and pruritic stimulus. We found that in all cases, the GFP^+^ cells were significantly underrepresented compared with GRP-negative neurons (Table 4). Examples of pERK staining in the GRP-eGFP mice are shown in Figure 11.

**Table 4:**
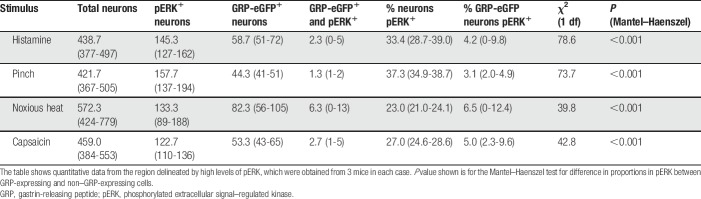
The numbers and percentages of GRP-eGFP neurons in laminae I and II that were pERK after noxious or pruritic stimulation.

## 4. Discussion

**Figure 12.**
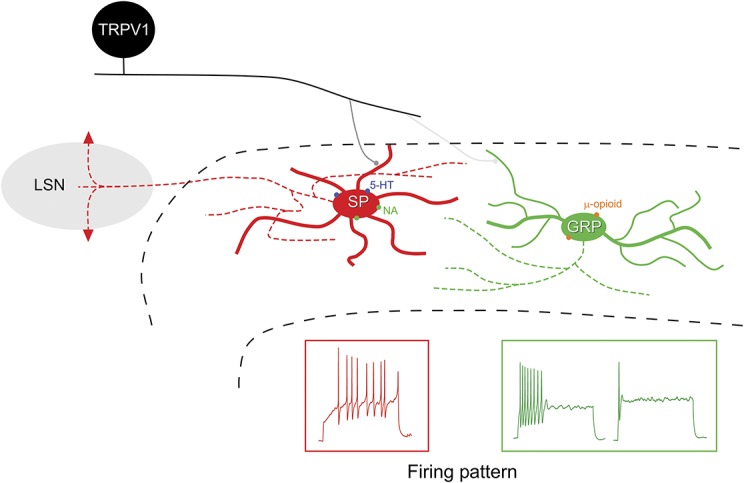
A diagram showing major differences that were detected between excitatory interneurons that express substance P (SP) and gastrin-releasing peptide (GRP). One cell of each type is represented on a transverse section of the dorsal horn, with dashed black lines indicating the borders of lamina II. The SP cells have more primary dendrites than the GRP cells, and typically show radial morphology, whereas the GRP cells were either central or unclassified cells. Note that these definitions are based on the somatodendritic morphology as seen in sagittal sections. Axons of both types (indicated with dashed lines of the corresponding colour) arborise locally in lamina II, whereas the SP cells also project to the lateral spinal nucleus (LSN), giving rise to propriospinal axons within this nucleus. Substance P cells generally responded (with outward current) to the monoamines noradrenaline (NA) and 5-hydroxytryptamine (5-HT), whereas the GRP cells responded with outward current to μ-opioid agonist. Around half of the SP cells showed an increase in mEPSC frequency in response to bath-applied capsaicin, indicating monosynaptic input from TRPV1-expressing primary afferents. However, this was only seen for 1 of 11 GRP cells tested, suggesting that these cells are seldom innervated directly by these afferents. The firing patterns in response to injected depolarising current pulses also differed, with most SP cells showing delayed firing, and GRP cells generally having transient or single-spike patterns. mEPSC, miniature excitatory postsynaptic current.

Our main findings are that SP- and GRP-expressing cells form largely nonoverlapping populations among the excitatory interneurons in laminae I and II, and that these differ significantly in morphology, firing patterns, EPSC frequency, and responses to neuromodulators. Comparison with previous data suggests that they also differ in responses to noxious and pruritic stimuli, and their contribution to propriospinal projections.^23,25^ These findings are summarised in Figure 12.

### 4.1. The function of gastrin-releasing peptide–expressing interneurons

It is well established that GRP-expressing neurons have a major role in itch.^46^ Sun et al.^60^ recently proposed that these cells respond weakly to pruritic stimuli and strongly to painful stimuli, but that when strongly activated, they suppress pain through feed-forward inhibition, forming a “leaky gate.” We previously reported that GRP cells rarely show pERK or Fos following intradermal chloroquine,^6^ and Sun et al. suggested that this was because of their weak activation by pruriceptors. However, we show here that these cells very seldom develop pERK after noxious stimuli. Interestingly, Sun et al.^60^ report that capsaicin applied to the dorsal root ganglion generated mean firing rates of <1 Hz in GRP cells, and if this is similar to their response to natural noxious stimuli, it may be inadequate to phosphorylate ERK. It has been reported that GRP cells are innervated by various types of primary afferent, including TRPV1-expressing nociceptors.^60^ However, our finding that capsaicin increased mEPSC and sEPSC frequency in only 1/11 and 4/16 GRP neurons, respectively, suggests that TRPV1-expressing afferents seldom directly innervate GRP cells, although they do provide polysynaptic input to some of them. The potential involvement of GRP cells in nociceptive processing therefore remains to be established.

At first sight, our finding that GRP cells were inhibited by DAMGO is surprising because MOR agonists can cause itching.^63^ However, it has been suggested that this is mediated by MOR1D-GRPR heterodimers on GRPR neurons.^39^ Because these cells are believed to be located downstream of GRP cells in the spinal itch pathway, activation of the GRPR cells by morphine acting on these heterodimers would presumably override its inhibitory action on the GRP cells. The expression of MORs by GRP neurons suggests that endogenous release of MOR agonists (eg, endomorphin-2) from nociceptors will inhibit activity in the itch pathway, contributing to suppression of itch by noxious stimuli. Although KOR agonists are antipruritic, they are believed to act at the level of the GRPR cells,^30^ consistent with the lack of response of GRP cells shown here. The finding that neither GRP nor SP cells responded to DOR agonist is consistent with the recent report that MOR and DOR are expressed by largely separate populations, with DOR-expressing cells being concentrated in lamina IIi.^68^ It should be noted that in addition to their action on cell bodies and dendrites of dorsal horn neurons, opioid peptides can also act on axon terminals to modify synaptic transmission. Little is known about the role of monoaminergic systems in itch, although tricyclic antidepressants, which potentiate monoamine transmission, are used to treat pruritus.^37^ The lack of effect of NA and 5-HT on GRP cells suggests that they act elsewhere in the itch pathway.

We found that there were consistently fewer GRP-eGFP cells in glabrous skin territory, and in situ hybridisation revealed that this was not due solely to lack of GRP-expressing neurons in this region. This variability in GFP expression presumably reflects transcriptional heterogeneity between GRP cells innervated from hairy and glabrous skin, and may be related to differences in itch sensation from these 2 skin types.^67^ As the GRP cells are relatively numerous, they presumably correspond to one or more of the populations identified by Grudt and Perl.^22^ Although many excitatory interneurons in lamina II show delayed/gap/reluctant firing,^29,49,73^ this was seldom seen for GRP cells, which generally showed transient or single-spike firing. Because the GRP cells had dendritic trees that were moderately elongated along the RC axis, many of them are likely to correspond to the “transient central” population of Grudt and Perl. Interestingly, transient central cells have been implicated in a circuit linking low-threshold mechanoreceptive afferents to lamina I projection neurons, which is believed to contribute to tactile allodynia.^40,41^ It will therefore be important to determine whether the cells in this putative circuit include GRP-expressing neurons, and how this relates to their proposed role in itch.

### 4.2. Substance P–expressing excitatory interneurons are radial cells

Unlike GRP cells, most SP neurons (79.2%) showed delayed firing, with a few having gap or reluctant patterns. As ∼10% of the cells labelled with this strategy are inhibitory interneurons,^23^ which seldom show these firing patterns, the proportion of excitatory SP interneurons with delayed/gap/reluctant firing is presumably even higher. Cluster analysis revealed a clear difference in dendritic morphology between SP and GRP cells. Substance P cells had significantly more primary dendrites, characteristic of radial cells. Alba-Delgado et al.^3^ reported that some PKCγ-immunoreactive cells in lamina II of the medullary dorsal horn have radial morphology. However, unlike the radial cells described here and in previous studies,^22,73^ PKCγ cells do not show delayed firing.^3,40^ Among other differences, SP cells showed higher mEPSC frequencies than GRP cells, suggesting that they receive more excitatory synapses. Consistent with radial cells receiving both Aδ and C inputs,^22,71^ 4 of 9 SP neurons showed a TRPV1-dependent, capsaicin-evoked increase in mEPSC frequency.

Little is known about the neuronal circuits engaged by radial cells. We previously reported that many excitatory SP neurons are activated by noxious or pruritic stimuli, and that many have long propriospinal axons targeting the LSN.^23,25^ Interestingly, Grudt and Perl^22^ reported that most radial cells had axons that ran rostrally and/or caudally in the dorsolateral fasciculus, consistent with our finding of propriospinal projections from these cells to the LSN. The SP cells may therefore contribute to the large cutaneous receptive fields, characteristic of LSN neurons in inflammatory pain states.^55^ Conceivably, this circuit serves a protective role because extension of pain beyond the site of damage would limit use of an injured limb during recovery. The scarcity of GRP cells among propriospinal interneurons probably reflects the different behavioural requirement for itch, where spatial acuity is needed to remove the underlying cause by scratching or biting.

Most SP cells were inhibited by NA and 5-HT, and similar results have been found for radial cells in rats.^42,73^ Inhibition of these cells from the brainstem may therefore contribute to the antinociceptive actions of descending monoaminergic pathways. Glycinergic neurons in deeper laminae of the dorsal horn provide another source of inhibition for radial cells,^71^ and it has been proposed that reduction in glycinergic input to radial cells contributes to neuropathic pain.^33^

Together, these findings suggest that the excitatory SP interneurons correspond to the radial cells identified by Grudt and Perl,^22^ and that they play an important role in pain mechanisms.

### 4.3. Excitatory interneuron populations

Two recent transcriptomic studies^27,53^ have defined neurochemically distinct populations of dorsal horn excitatory neurons. Although both studies identified the SP, NKB, and neurotensin populations that we previously described,^25,66^ only one recognised the GRP cells as a distinct population.^53^ In that study, the GRP (but not SP) population was reported to express the MOR gene (OPMR1), consistent with our finding that GRP (but not SP) cells possess functional MORs. Haring et al.^27^ did not detect a specific population of GRP-expressing cells, and reported considerable overlap between GRP and Tac1 mRNAs. However, our in situ hybridisation data clearly show that GRP-expressing cells in lamina II differ from those that express SP, suggesting that they are indeed a distinct neurochemical population.

Vertical cells constitute a well-defined class of excitatory interneurons, with cell bodies in lamina IIo and ventrally directed dendrites.^22,43,49,71–73^ They are presynaptic to lamina I projection neurons^12,41^ and have been implicated in transmitting low-threshold mechanoreceptive information to the projection cells, thus contributing to tactile allodynia.^40^ The present results show that neither SP nor GRP populations include vertical cells, and because the neurotensin and NKB neurons are found in laminae IIi and III, these are also unlikely to include vertical cells. Finding the neurochemical signature of vertical cells is therefore a high priority. In this regard, we recently identified a cluster of dynorphin-expressing excitatory interneurons with vertical cell morphology; however, these are largely restricted to glabrous skin territory.^30^ Two additional classes of excitatory interneuron identified in transcriptomic studies consist of cells that express neuropeptide FF or the neuromedin 2 receptor (NMUR2),^27,53^ and in both cases, the cells are concentrated in lamina IIo.^11,27^ It will therefore be important to test whether either of these populations corresponds to vertical cells, as this would allow for selective genetic targeting of these cells to investigate their role in spinal pain circuits. Because both SP and GRP populations have axons that arborise mainly in lamina II, vertical cells may also provide the route through which these cells can activate lamina I projection neurons. Defining the circuits that link the different populations of excitatory interneurons will therefore be of considerable importance.

## Conflict of interest statement

The authors have no conflicts of interest to declare.

## Supplementary Material

SUPPLEMENTARY MATERIAL

## Supplemental video content

Video content associated with this article can be found online at http://links.lww.com/PAIN/A665.
