# Supplementary material for: Morphological and functional properties distinguish the substance P and gastrin-releasing peptide subsets of excitatory interneuron in the spinal cord dorsal horn
Source: Pain. 2018 Sep 20;160(2):442–62. doi: 10.1097/j.pain.0000000000001406 (PMC6330098; doi:10.1097/j.pain.0000000000001406)
Supplement: SUPPLEMENTARY MATERIAL [file jop-160-442-s001.docx]

Morphological and functional properties distinguish the substance P and gastrin-releasing peptide subsets of excitatory interneuron in the spinal cord dorsal horn

Allen C. Dickie^1^*, Andrew M. Bell^1^*, Noboru Iwagaki^1^*, Erika Polgár^1^, Maria Gutierrez-Mecinas^1^, Rosalind Kelly^1^, Heather Lyon^1^, Kirsten Turnbull^1^, Steven J. West^2^, Alexander Etlin^3^, Joao Braz^3^ Masahiko Watanabe^4^, David L.H. Bennett^2^, Allan I. Basbaum^3^, John S. Riddell^1^ and Andrew J. Todd^1^

^1^Spinal Cord Group, Institute of Neuroscience and Psychology, University of Glasgow, Glasgow G12 8QQ, UK; ^2^The Nuffield Department of Clinical Neurosciences, University of Oxford, John Radcliffe Hospital, Oxford, OX3 9DU, UK; ^3^Department of Anatomy, University of California, San Francisco, San Francisco, CA 94158; ^4^Department of Anatomy, Hokkaido University School of Medicine, Sapporo 060-8638, Japan

*These authors contributed equally

51 text pages, 12 figures, 4 tables

Correspondence to: A.J. Todd, Spinal Cord Group, West Medical Building, University of Glasgow, University Avenue, Glasgow G12 8QQ, U.K.

Phone: (+44) 141 330 5868. Fax: (+44) 141 330 2868. email: andrew.todd@ glasgow.ac.uk

Keywords: glutamatergic interneuron, radial cell, central cell, GRP, pain

**ABSTRACT**

Excitatory interneurons account for the majority of neurons in the superficial dorsal horn, but despite their presumed contribution to pain and itch, there is still limited information about their organisation and function. We recently identified two populations of excitatory interneuron defined by expression of gastrin-releasing peptide (GRP) or substance P (SP). Here we demonstrate that these cells show major differences in their morphological, electrophysiological and pharmacological properties. Based on their somatodendritic morphology and firing patterns, we propose that the SP cells correspond to radial cells, which generally show delayed firing. In contrast, most GRP cells show transient or single-spike firing, and many are likely to correspond to the so-called transient central cells. Unlike the SP cells, few of the GRP cells had long propriospinal projections, suggesting that they are involved primarily in local processing. The two populations also differed in responses to neuromodulators, with most SP cells, but few GRP cells, responding to noradrenaline and 5-HT; the converse was true for responses to the μ-opioid agonist DAMGO. Although a recent study suggested that GRP cells are innervated by nociceptors and are strongly activated by noxious stimuli [60], we found that very few GRP cells receive direct synaptic input from TRPV1-expressing afferents, and that they seldom phosphorylate extracellular signal-regulated kinases in response to noxious stimuli. These findings indicate that the SP and GRP cells differentially process somatosensory information.

**Introduction**

The superficial laminae (I-II) of the spinal dorsal horn are the main target for nociceptive and pruriceptive primary afferents. Consequently, neurons within this region are involved in transmitting and modulating signals perceived as pain and itch. While some of these cells project to the brain, most (~99%) are interneurons [1,8,65]. Although early models emphasised the importance of inhibitory interneurons in gating nociceptive inputs [45], the majority of interneurons in these laminae are glutamatergic excitatory cells [66], and these are both functionally and morphologically heterogeneous [22,49,56,71,73]. Recent studies demonstrated that the excitatory interneurons are essential for normal perception of pain and itch, and suggested that particular subgroups process distinct sensory modalities [16,69]. It is therefore important to define functional populations among these cells.

Among the morphologically distinct classes of excitatory interneurons are vertical cells, which have ventrally-directed dendrites; radial cells, which have compact, highly branched dendritic trees; and central cells, which have rostrocaudally orientated dendrites. These differ in physiological properties [22,71], and may therefore correspond to functional populations. However, many excitatory interneurons cannot be assigned to a particular morphological class [73], and we have developed an alternative classification scheme based on the largely non-overlapping expression of four neuropeptides: neurotensin, neurokinin B (NKB), gastrin-releasing peptide (GRP) and substance P (SP) [23,24]. Together, these account for over half of the excitatory interneurons in laminae I-II. Neurotensin and NKB neurons are concentrated in inner lamina II (IIi), and overlap extensively with cells expressing protein kinase Cγ (PKCγ). GRP-expressing neurons are found throughout lamina II, and can been identified in a GRP::eGFP BAC transgenic mouse [57]. GRP cells have attracted particular interest because of their contribution to itch [46,60]. GRP released from these cells targets the GRP receptor (GRPR), which is expressed by a different population of excitatory interneurons in lamina I and outer lamina II (IIo) [61,62]. GRP cells are innervated by pruriceptive afferents that express MrgA3, a receptor for the pruritogen chloroquine [60], and are thought to act as "secondary pruriceptors" in a pathway linking pruriceptive afferents to GRPR-expressing interneurons [46], which innervate lamina I projection neurons. SP is cleaved from a precursor, pre-protachykinin A (PPTA) coded by *Tac1*, and SP-expressing neurons can be identified by intraspinal injection of adeno-associated viruses (AAVs) containing Cre-dependent expression cassettes into mice in which Cre-recombinase is knocked into the *Tac1* locus (Tac1^Cre^). Using this approach, we found that SP-expressing cells are located mainly in lamina IIo, somewhat dorsal to GRP cells, although the populations show some spatial overlap [23]. Although SP-expressing neurons include some projection neurons and inhibitory cells, the great majority are excitatory interneurons [23,25].

Despite the non-overlapping expression pattern of these neuropeptides, we cannot be certain that GRP and SP cells represent distinct functional populations. Here we used a variety of techniques to characterise and compare GRP- and SP-expressing neurons in mouse superficial dorsal horn. We find that these two populations differ widely in anatomical, electrophysiological and pharmacological properties, suggesting that they represent distinct populations that are likely to contribute differentially to somatosensory processing.

## Methods

Experiments were approved by the Ethical Review Process Applications Panel of the University of Glasgow and were performed in accordance with the UK Animals (Scientific Procedures) Act 1986 and the University of California, San Francisco’s Institutional Animal Care and Use Committee guidelines.

*Animals*

We used two genetically modified mouse strains: a BAC transgenic Tg(GRP-EGFP) from GENSAT in which GFP is expressed under control of the GRP promoter [19,26,46,57], and a line in which Cre recombinase is inserted into the *Tac1* locus (Tac1-IRES2-Cre-D; Jackson Laboratory, Bar Harbor, ME; Stock number 021877) [28]. These lines are referred to as GRP::eGFP and Tac1^Cre^, respectively. GRP::eGFP mice were maintained as heterozygotes, while most of the Tac1^Cre^ mice were homozygous. Both strains were maintained on a C57BL/6 background. For some experiments the two lines were crossed to produce double transgenic mice (Tac1^Cre^;GRP::eGFP). Unless otherwise stated, mice of either sex weighing between 14-28 g were used in all parts of the study. Most of the mice that were used for anatomical studies underwent perfusion fixation. They were deeply anaesthetised with pentobarbitone (20 mg, intraperitoneally) and perfused through the heart with a fixative that contained 4% freshly depolymerised formaldehyde in phosphate buffer. Spinal cord tissue was rapidly dissected out and post-fixed at 4°C for 2 hours (unless stated otherwise).

*Intraspinal injection*

Intraspinal injections were performed to deliver viral vectors coding for Cre-dependent constructs into Tac1^Cre^ or Tac1^Cre^;GRP::eGFP mice, and to deliver the retrograde tracer cholera toxin B subunit (CTb) into GRP::eGFP mice. Table 1 lists the viral vectors used. To identify SP cells, we used AAVs coding for Cre-dependent eGFP or tdTomato, and to investigate the somatodendritic morphology of these cells we used AAV-Brainbow vectors [9]. The injections used a modification of the method of Foster et al [17] as described previously [23]. The mice were anaesthetised with 1-2% isoflurane and placed in a stereotaxic frame. For the experiments involving AAVs, 2 injections were made in each animal, either into the L3 and L5 segments on one side, or else bilaterally into either the L3 or L5 segments. The vertebral column was exposed, and vertebral clamps were attached to the T12 and L1 vertebrae. The space between the laminae of T12-T13 was used for L3 injections and that between laminae of T13-L1 for L5 injections. In each case, a small incision was made in the dura to the side of the midline, and injections were made through glass micropipettes (inner diameter of tip 40 μm) into the spinal dorsal horn. Injections were made 300-500 μm lateral to the midline at a depth of 300 μm below the pial surface, and were administered at a rate of 30 nl/min. The wound was then closed, and animals were allowed to recover with appropriate analgesia (buprenorphine 0.3 mg/kg and carprofen 5 mg/kg). Injections of CTb into the GRP::eGFP mice were targeted on the T13 spinal segment. These injections were carried out as described above, except that they were made through the space between the laminae of T11 and T12 vertebrae [25]. In each case, a single injection (300 nl of 1% CTb) was made into the dorsal horn on the right side, and the animals survived for 4 days before perfusion fixation [25].

*General features of immunocytochemistry and characterisation of antibodies*

Multiple-labelling immunofluorescence microscopy was carried out as described previously [23]. Table 2 lists the sources and concentrations of the antibodies used. Briefly, spinal cord segments were cut into 60 μm thick transverse or parasagittal sections with a vibrating blade microtome (Leica VT1200) and these were treated with 50% ethanol for 30 mins to enhance antibody penetration. Sections were incubated for 3 days at 4°C in mixtures of primary antibodies and then overnight in mixtures of species-specific secondary antibodies that were raised in donkey. The secondary antibodies were conjugated to Alexa 488, Alexa 647, Rhodamine Red, Pacific Blue or biotin (Jackson ImmunoResearch,West Grove, PA) and were used at 1:500 (Alexa 488, Alexa 647, biotin), 1:200 (Pacific Blue) or 1:100 (Rhodamine Red). Biotinylated secondary antibodies were detected with a tyramide signal amplification (TSA) method (TSA kit tetramethyl­rhodamine NL702, PerkinElmer Life Sciences, Boston, MA, USA) as described previously [24]. After immunoreaction, the sections were mounted in antifade medium and stored at -20°C. Unless otherwise stated, sections were scanned with a Zeiss LSM710 confocal microscope equipped with Argon multi-line, 405 nm diode, 561 nm solid state and 633 nm HeNe lasers. In all cases, the confocal aperture was set to 1 Airy unit or less.

The CTb antibody was raised against the purified protein, and specificity is demonstrated by the lack of staining in regions that did not contain injected or transported tracer. The chicken and rabbit antibodies against GFP were raised against recombinant full-length eGFP and the staining matches that of native GFP fluorescence. The PKCγ antibodies, which were raised against amino acids 648-697 of the mouse protein, detect a single band at 75 kDa in tissue from wild-type (but not PKCγ^-/-^) mice, and the guinea pig antibody stains identical structures to those detected by a well-characterised rabbit antibody [52,74]. The VGLUT3 antibody was raised against amino acids 522-588 of the mouse protein, and detects a single band at 60-62 kDa. The mouse monoclonal NeuN antibody was raised against cell nuclei extracted from mouse brain and found to react with a protein specific for neurons [47], which has subsequently been identified as the splicing factor Fox-3 [36]. The guinea pig antibody was raised against a recombinant protein consisting of amino acids 1-97 of Fox-3 and immunostains the same cells as the mouse antibody. The mCherry antibody was raised against a full-length recombinant protein, while the TFP and tagRFP antibodies were raised against the corresponding purified proteins. Their specificity is demonstrated by the lack of staining in tissue that lacks these fluorescent proteins. The Pax2 antibody is directed against amino acids 188-385 of the mouse protein, and recognizes bands of the appropriate size on Western blots of mouse embryonic kidney [15]. The pERK antibody detects p44 and p42 MAP kinase (Erk1 and Erk2) when these are phosphorylated either individually or dually at Thr202 and Tyr204 of Erk1 or Thr185 and Tyr187 of Erk2. This antibody does not cross-react with the corresponding phosphorylated residues of JNK/SAPK or of p38 MAP kinase, or with non-phosphorylated Erk1/2 (Manufacturer's specification). Specificity is demonstrated by the lack of staining in non-stimulated areas (e.g. in the contralateral dorsal horn).

*Distribution of GRP::eGFP cells and their relation to GRP mRNA*

To assess differences in the distribution of GRP-eGFP cells in different regions of the lumbar spinal cord, we examined the lateral and medial regions of the superficial dorsal horn in the L1 and L4 segments using immunostained sections. These segments were chosen because they differ in the extent to which they receive input from glabrous skin [30], and preliminary observations indicated that GFP^+^ cells were less numerous in glabrous skin territory. Scans were analysed with Neurolucida for Confocal (MBF Bioscience). We used a modification [48] of the disector method [59] to quantify the proportion of all neurons that were GFP positive in sections from 3 perfusion-fixed GRP::eGFP mice. These sections had been immunoreacted to reveal GFP (chicken antibody), VGLUT3 and NeuN (mouse antibody), and counterstained with 4',6-diamidino-2-phenylindole (DAPI). Three sections from the L1 and L4 segments of each mouse were scanned with the confocal microscope through a 40× oil-immersion lens (numerical aperture 1.3) to include the entire mediolateral extent of the dorsal horn through the whole depth of the section (2 μm z-spacing). The reference and lookup sections were placed 20 μm apart, and all optical sections between these were carefully examined. All neuronal nuclei (defined by the presence of NeuN and DAPI) with their bottom surface between the reference and look-up sections were identified and plotted onto an outline of the dorsal horn. The channel corresponding to GFP was then viewed, and the presence or absence of immunoreactivity was noted for each selected neuron. Scans from L4 were divided into medial and lateral compartments based on the pattern of VGLUT3 immunoreactivity, which is expressed by C-low threshold mechanoreceptors (C-LTMRs) that are restricted to the lateral "hairy skin" region [38,54]. Since the whole of the L1 segment is innervated from hairy skin, we divided the dorsal horn into medial and lateral halves in this segment.

*In situ* hybridisation was used to examine the relationship between GRP mRNA and GFP in the L4-5 segments from 3 perfusion-fixed GRP::eGFP mice. Tissue from these mice was post-fixed for 4-12 hours, cryoprotected in 30% sucrose, embedded in OCT mounting medium and stored at ‑80°C. Transverse sections (12 μm thick) were cut with a cryostat, mounted onto SuperFrost Plus slides (48311-703, VWR) and air dried. The RNAscope procedure was performed according to the supplied protocol. Briefly, tissue was incubated in peroxide solution, dehydrated in 100% ethanol, and reacted with protease in aqueous buffer. The RNAscope probe against GRP (Mm-GRP, 317861, RNAscope) and a negative control probe were incubated with tissue sections at 40°C for 2 hours, followed by washing in RNAscope wash buffer and a series of incubations in amplification buffers (1-6, 15-30 minutes at 40°C – room temperature), and finally detected using the Red Assay kit (322360, RNAscope). Sections were then incubated for 24 hours at room temperature in GFP antibody (rabbit), and then in secondary antibody conjugated to Alexa 488. They were then counterstained with DAPI and mounted in anti-fade medium. Sections were scanned with a Zeiss LSM 700 laser-scanning confocal microscope. The images were acquired by scanning a single optical plane through the centre of the section depth with a 40× oil-immersion lens (numerical aperture 1.3), to include both dorsal horns for each section.

Cells that were positive for GRP mRNA and GFP were quantified manually in ImageJ by using the Cell Counter Plugin. Cells in lamina I-II of the dorsal horn were counted, and a minimum of 3 sections were assessed per animal. The dorsal horn was divided into medial glabrous and lateral hairy skin innervation territories, defined by the intense plexus of GFP labelling seen in hairy skin territory in the GRP:eGFP mice (see below). Initially the mRNA channel was examined, and cell bodies (defined by DAPI labelling) that contained 3 or more intense mRNA puncta were counted. Next, the GFP labelling was viewed to reveal the proportion of GFP cells that were positive for GRP mRNA. Finally, the number of GFP positive cells that lacked GRP mRNA was determined. Images were assessed against the negative control probe, which did not show any specific or intense *in situ* hybridisation product.

*Relationship between GRP- and SP-expressing cells*

To determine the extent of overlap between GRP::eGFP and SP populations, we injected AAV coding for a Cre-dependent form of tdTomato (AAV.flex.tdTom) into two Tac1^Cre^;GRP::eGFP male mice. The virus encodes an inverted sequence for tdTomato between pairs of heterotypic LoxP sites with anti-parallel orientation [4], resulting in expression of tdTomato only in transfected cells that contain Cre. After a two-week survival period the mice were reanaesthetised and perfused with fixative. Sections through the injection site of both mice were immunostained with antibodies against GFP (rabbit) and PKCγ (goat), and two sections containing numerous tdTom^+^ cells were selected and scanned with the confocal microscope. The scans were analysed with Neurolucida. Initially, the channel corresponding to tdTom was viewed, and all labelled cells were plotted onto an outline of the superficial dorsal horn (lamina I and II). The other 2 channels were then viewed, and the presence or absence of GFP and PKCγ was noted for each tdTom cell. Any additional cells that were GFP^+^ and/or PKCγ^+^ were then added to the drawing. Although a stereological method was not used in this part of the study, the low level of double-labelling for tdTom and GFP (see below) is unlikely to have been affected by sampling bias. We have previously shown that following intraspinal injection of AAV coding for a Cre-dependent form of eGFP into Tac1^Cre^ mice, virtually all of the cells near the injection site that are immunoreactive for the SP precursor PPTA are GFP-positive, indicating that this procedure captures a high proportion of SP-expressing cells [23]. We have also reported that it results in GFP-labelling of ~20% of neurons in the superficial dorsal horn within 4 days of the injection, and that this percentage does not change when animals are allowed to survive for 8 days after the spinal injection [25], suggesting that a stable expression pattern is reached very soon after the injection.

To compare expression of SP with the distribution of GRP mRNA, we injected AAV.flex.eGFP into 3 male Tac1^Cre^ mice. One or 2 weeks later, the mice were reanaesthetised and fixed by perfusion. Tissue from these mice was processed with *in situ* hybridisation histochemistry to reveal GRP mRNA and GFP. Both the tissue processing and analysis were carried out as described above.

To provide further evidence about the extent of co-expression of SP and GRP, we performed double-label fluorescence *in situ* hybridisation on tissue from 4 adult wild-type (C57Bl/6) mice, using RNAscope probes against mRNAs for GRP and substance P. The reaction was performed on 12 μm thick cryostat sections from C4-C6 segments (from 3 mice) and the L3-5 segment (from one mouse) according to the supplied protocol. Sections were counterstained with DAPI to allow identification of cell nuclei.Single RNA transcripts for each target gene appeared as punctate dots (usually around the DAPI-stained nucleus) and we considered a cell to be positive if it contained more than 5 dots. Quantification was performed on a series of spinal cord sections (1 in every 4) from 3 separate animals. We analysed between 5-8 sections per animal. Only positive cells containing a DAPI-stained nucleus in the dorsal horn (laminae I-V), were included in the analysis. The percentage of double-labeled cells was calculated by dividing the number of double-labeled neurons by the number of single-labeled neurons for each probe.

*Slice preparation and electrophysiology*

Electrophysiological recordings from both SP and GRP neurons were performed on spinal cord slices. For those involving SP neurons, we used Tac1^Cre^ mice that had received intraspinal injection of either AAV.flex.eGFP or AAV.flex.tdTom between 1 and 3 weeks previously. Spinal cord slices were obtained from 40 injected Tac1^Cre^ mice and from 105 GRP::eGFP mice aged 4 to 10 weeks old, as described previously [14,34]. The spinal cord was removed either following laminectomy performed under isoflurane anaesthesia, or in ice-cold dissection solution after decapitation of the mice under brief isoflurane or tribromoethanol anaesthesia. Mice from which the cord was removed under anaesthesia were decapitated immediately afterwards. Parasagittal (300 – 500 μm), transverse (400 – 600 μm) or horizontal (400 μm) slices from lumbar spinal cord were cut with a vibrating blade microtome and allowed to recover in recording or modified dissection solution for at least 30 minutes at room temperature [14,34]. In some cases the slices were placed in a NaCl-based recovery solution or an *N*-methyl-D-glucamine (NMDG) -based recovery solution [64] at 32°C for 15 minutes before being placed in recording solution at room temperature. The solutions used contained the following (in mM): Dissection, 3.0 KCl, 1.2 NaH_2_PO_4_, 0.5-2.4 CaCl_2_, 1.3-7.0 MgCl_2_, 26.0 NaHCO_3_, 15.0-25.0 glucose, 240.0-251.6 sucrose; Modified dissection, 3.0 KCl, 1.2 NaH_2_PO_4_, 0.5 CaCl_2_, 1.3 MgCl_2_, 8.7 MgSO_4,_ 26.0 NaHCO_3_, 20.0 HEPES, 25.0 glucose, 215.0 sucrose, with or without 1.0 kynurenic acid; NaCl recovery solution, 125.0 NaCl, 2.5 KCl, 1.25 NaH_2_PO_4_, 1.5 CaCl_2_, 6.0 MgCl_2_, 26.0 NaHCO_3_, 25.0 glucose; NMDG recovery solution, 93.0 NMDG, 2.5 KCl, 1.2 NaH_2_PO_4_, 0.5 CaCl_2_, 10.0 MgSO_4_, 30.0 NaHCO_3_, 25.0 glucose, 5.0 Na-ascorbate, 2.0 thiourea, 3.0 Na-pyruvate, 20.0 HEPES; Recording 125.0-127.0 NaCl, 2.5-3.0 KCl, 1.2-1.25 NaH_2_PO_4_, 2.0-2.4 CaCl_2_, 1.0-1.3 MgCl_2_, 26.0 NaHCO_3_,15.0-25.0 glucose. In some cases, dissection was performed in NaCl recovery solution with 1 mM kynurenic acid. All solutions were bubbled with 95% O_2_ / 5% CO_2_.

Targeted whole-cell patch-clamp recordings were made from GFP-positive cells (slices from GRP::eGFP mice or Tac1^Cre^ mice with AAV.Flex.eGFP injection) or tdTomato-positive cells (Tac1^Cre^ mice with AAV.Flex.tdTom injection) in the superficial dorsal horn, using patch pipettes that had a typical resistance of 3-7 MΩ when filled with an intracellular solution containing (in mM): 130.0 K-gluconate, 10.0 KCl, 2.0 MgCl_2_, 10.0 HEPES, 0.5 EGTA, 2.0 ATP-Na_2_, 0.5 GTP-Na, and 0.2% Neurobiotin, pH adjusted to 7.3 with 1.0M KOH. In some cases the pipette solution contained (in mM): 120.0 K-methanesulphonate, 10.0 NaCl, 1.0 CaCl_2_, 10.0 HEPES,10 EGTA; 5.0 ATP-Mg, 0.5 GTP-Na. Data were recorded and acquired with a Multiclamp 700B amplifier and pClamp 10 software (both Molecular Devices), and were filtered at 4 kHz and digitised at 10 kHz.

After achieving stable whole-cell configuration the cells were voltage clamped at -60 mV and a series of 100 ms voltage steps from -70 to -50 mV (2.5 mV increments) delivered to determine the current-voltage relationship, which was used to calculate resting membrane potential and input resistance. Cells that had a resting membrane potential that was less negative than -30 mV were excluded from all analysis.

The action potential firing pattern was assessed in current clamp mode, in response to 1s depolarising current steps of increasing amplitude, from a membrane potential of around -60 mV. Firing patterns were classified on the basis of previously published criteria [18,20,22,51,56,73]. Cells were classed as tonic firing if they exhibited continuous action potential discharge throughout the depolarising step; transient if the action potential discharge ocurred only during the early part of the step; delayed if there was a clear delay between the start of the depolarising step and the first action potential; single spike if only one or two action potentials occurred at the onset of the step; gap if there was a long first interspike interval; and reluctant if current injection did not result in action potential firing. It has recently been reported that reluctant firing may result from high levels of expression of both a low-threshold non-inactivating potassium conductance and an inactivating (A-type) potassium conductance [5].

Subthreshold voltage-activated currents were investigated by voltage-clamping the cell at ‑60 mV before stepping to -90 mV for 1s then to -40 mV for 200ms, and automated leak subtraction was used to remove capacitive and leak currents [21,56]. This step protocol enables the identification of two types of transient outward current and two types of inward current [21]. The outward currents that occur during the depolarising step (-90 to -40 mV) are consistent with A-type potassium currents (I_A_), and on the basis of their kinetics can be distinguished as rapid (I_Ar_) or slow (I_As_). A transient inward current can be observed during the depolarising step, which is considered to reflect the low threshold ‘T-type’ calcium current (I_ca,T_). A slow inward current can occur during the hyperpolarisation step (-60 to ‑90 mV) that is consistent with the hyperpolarisation-activated (I_h_) current. The amplitude of I_Ar_ was measured as the peak of the transient outward current. The amplitude of the I_h_ current was measured during the final 200 ms of the hyperpolarising step, and inward currents were classified as I_h_ if the amplitude was greater than -5 pA.

Excitatory synaptic input to GRP-eGFP and SP neurons was assessed by recording spontaneous EPSCs (sEPSCs) at a holding potential of -60 or -70 mV. The functional expression of TRP channels on the afferents providing synaptic input was investigated by voltage clamping cells at -60 or -70 mV and recording sEPSCs or miniature EPSCs (mEPSCs), the latter in the presence of tetrodotoxin (TTX) (0.5 μM), bicuculline (10 μM) and strychnine (5 μM), prior to and during the bath application of the TRPV1 agonist capsaicin (2 μM) or the TRPM8 agonist icilin (20 μM; mEPSCs only). In the case of icilin application the temperature of the bath was raised to 32°C [18]. These data were analysed using Mini Analysis (Synaptosoft), sEPSC/mEPSC events were automatically detected by the software and were then rejected or accepted following visual examination. Neurons were considered to receive input from capsaicin- or icilin-sensitive afferents if capsaicin/icilin application resulted in a significant leftwards shift in the distribution of inter-event intervals, indicating an increase in frequency. They were considered non-responsive if this threshold was not reached.

The response of GRP-eGFP and SP cells to a number of pharmacological agents was investigated by voltage clamping cells at -60 or -70 mV, and bath applying one of the following; 5‑HT (10-20 μM), noradrenaline (20 μM), the μ-opioid receptor (MOR) agonist DAMGO (3 μM), the κ-opioid (KOR) agonist U69593 (1 μM) or the δ-opioid (DOR) agonist [D-Ala^2^]-Deltorphin II (1 μM). Cells were considered responsive if drug application resulted in a clear slow outward current, and non-responsive if no current was seen.

All chemicals were obtained from Sigma except: TTX (Alomone), Bicuculline (Tocris), Neurobiotin (Vector Labs).

*Morphological analysis of GRP and SP cells*

Reconstruction of the GRP neurons (n = 45) was carried out on Neurobiotin-labelled cells that had been recorded in electrophysiological experiments. For the SP neurons (n = 31), analysis of cell bodies and dendritic trees was performed on tissue from mice that had been injected with Brainbow viruses [9]. In addition, a few Neurobiotin-labelled SP neurons (n = 12) from electrophysiological experiments were examined to allow reconstruction of their local axonal arbors.

Processing to reveal Neurobiotin in patched cells was performed as described previously [18,34]. Briefly, fixed slices containing recorded cells were incubated in avidin-Rhodamine (1:1000; Jackson ImmunoResearch) in PBS containing 0.3% Triton X-100 and mounted on slides. In a few cases, recordings had been made from Tac1^Cre^ mice that were injected with AAV.flex.tdTom, and in these cases avidin-Alexa488 was used instead of avidin-Rhodamine. The sections were scanned with the confocal microscope through a 63× oil-immersion lens (numerical aperture 1.4) at 0.5 μm z spacing. These scans included all of the dendritic and axonal arbors that were visible at this stage. For each cell, the presence of GFP or tdTom was confirmed by scanning for the native protein. The dendritic and axonal arbors were reconstructed in Neurolucida. Axons could easily be distinguished from dendrites based on their thin non-tapering profiles and the presence of irregularly-spaced boutons. In contrast, dendrites showed progressive tapering and invariably gave rise to dendritic spines [34]. Slices were then re-sectioned at 60 μm thickness with the vibrating blade microtome, and sections were kept in serial order. Sections were examined with the confocal microscope, and if additional parts of the dendritic or axonal tree located deep within the slice were found, these were added to the reconstruction. To determine laminar boundaries, we immunostained one section from each slice to reveal PKCγ, using a guinea pig antibody. PKCγ is present in a plexus of dendrites that occupies the inner half of lamina II (IIi) [31]. The boundaries between laminae II/III were then added to the reconstructions, by aligning sections containing the recorded cells with nearby sections stained for PKCγ. The lamina I/II border was taken to be 20 μm below the dorsal white matter [18], and this was also added to the reconstruction. To investigate the axonal morphology of SP neurons, we also reconstructed axonal arbors of 12 of these cells that had undergone whole-cell patch-clamp recording. These cells were processed, scanned and analysed as described above, except that in this case the PKCγ immunoreaction was carried out on the intact slice.

Two Tac1^Cre^ mice (one male, one female) received injections of two Brainbow AAV vectors [9]. One of these codes for enhanced yellow fluorescent protein (eYFP) and Tag blue fluorescent protein (TagBFP), and the other for teal fluorescent protein (TFP) and mCherry. In both cases, the sequences are for farnesylated fluorescent proteins in reverse orientation between anti-parallel LoxP sites. Cre-mediated recombination leads to random expression of one of the fluorescent proteins (or neither fluorescent protein) for each virus, and the presence of multiple copies of both viruses within cells results in a wide range of hues, resulting from varying amounts of each membrane-targeted fluorescent protein. Two weeks after the spinal injection, the mice were perfused with fixative, and tissue from these animals was used to investigate the morphology of SP-expressing neurons. Sagittal sections (60 μm thick) from the lumbar enlargement were reacted with antibodies against mCherry, mTFP, tagRFP (which recognises tagBFP) and Pax2, and these were revealed with secondary antibodies. Regions close to the injection site that contained numerous labelled cells were selected and scanned with the confocal microscope through the 63× lens at 0.5 μm z-spacing. Tile scans were obtained through the full thickness of the section, to include approximately 400 μm along the rostrocaudal axis and the entire dorsoventral extent of laminae I-II. Thirty-one cells (15, 16 from the 2 mice) were selected, based on the following criteria: (1) relatively strong staining for at least one of the fluorescent proteins; (2) location of the soma in the mid region of the z-axis, such that the entire dendritic tree was likely to be contained within the section; (3) the presence of a distinctive colour hue that contrasted with that of nearby labelled cells. Cell bodies and dendritic trees of SP neurons were drawn in Neurolucida by following colour-coded processes originating from the soma of the selected cells. Although this allowed reconstruction of dendritic trees, it was not possible to follow axons beyond their initial segments, due to their very small diameter.

Morphometric data for dendritic trees of all of the reconstructed cells were obtained from Neurolucida Explorer. The dendritic parameters extracted were the same as those used in our previous study [18]. To make an objective comparison between the GRP and SP cells, we carried out cluster analysis using Ward's method [70], as described previously [18]. To reduce the dimensionality of the original data while preserving variance, we calculated principal components from the dataset [13]. The number of principal components to be retained for cluster analysis was then determined from a scree test.

*Potential propriospinal projections of GRP-eGFP cells*

Bice and Beal [7] reported that a proportion of neurons in superficial dorsal horn have long propriospinal axons. We recently reported that around 40% of excitatory interneurons in laminae I-II of the mouse L5 segment have axons that project at least 5 segments rostrally, and that SP cells are over-represented among the neurons with these propriospinal projections [25]. To determine whether GRP-expressing neurons also give rise to long propriospinal axons, we examined the L5 segments of 4 GRP::eGFP mice that had received injections of CTb into the T13 segment.

Injection sites were assessed by reacting transverse sections through T13 with an immuno­peroxidase method [10]. The sections were incubated in anti-CTb at 1:200,000, followed by biotinylated secondary antibody and avidin-peroxidase, which was revealed with diaminobenzidine in the presence of H_2_O_2_. Transverse sections from the L5 segments were reacted with antibodies against CTb, NeuN (mouse or guinea pig) and GFP (chicken). These antibodies were detected with fluorescent secondary antibodies and the sections were stained with DAPI. Four sections from each mouse were selected for analysis, before the relationship between CTb and GFP was observed. The sections were scanned through the 40× oil-immersion lens to generate z-series (at least 20 optical sections at 1 μm z-separation) such that the entire cross-sectional area of the ipsilateral dorsal horn was included. The confocal scans were analysed with Neurolucida by using the modified disector method (see above). The reference and look-up sections were set 10 μm apart. Because the quantitative analysis was performed on laminae I-II, we first plotted the outline of the dorsal horn grey matter, and then located the lamina II-III border, based on the relatively low density of neurons in lamina IIi. The channels corresponding to NeuN and DAPI were initially viewed, and all neurons for which the bottom surface of the nucleus lay between the reference and look-up sections were marked on the drawing. The GFP and CTb channels were then examined, and for each of the selected neurons, the presence or absence of both GFP and CTb was determined. In this way, we determined the proportions of all lamina I-II neurons, and of GRP-eGFP neurons, in the L5 segment that were retrogradely labelled with CTb.

Because few GRP cells in L5 were retrogradely labelled from the T13/L1 injection site (see Results), a more limited analysis was also performed on sections from the L2 segment of two of these mice, in order to look for evidence of short intersegmental projections.

*Phosporylation of ERK following noxious and pruritic stimuli*

We previously demonstrated that SP cells in laminae I-II often express the transcription factor Fos [32] or phosphorylate extracellular signal-regulated kinases (ERK) [35] in response to a variety of noxious and pruritic stimuli [23]. In addition, we reported that GRP cells seldom show phosphorylated ERK (pERK) following injection of the pruritogen chloroquine [6]. Here, we used phosphorylation of ERK to test whether the GRP cells respond to other pruritic or to noxious stimuli.

Fifteen GRP::eGFP mice were used in these experiments (n = 3 mice per stimulus type). In all cases, stimuli involved the left calf (which had been shaved on the day before stimulation), because of the relatively low density of GRP-eGFP cells in regions of dorsal horn that are innervated from glabrous skin (see below). Twelve of the mice received noxious stimuli or vehicle injection. These animals had been anaesthetised with urethane (40-80 mg i.p.) and the stimulus was applied 5 minutes prior to perfusion fixation. The stimuli were: (i) immersion of the leg up to the level of the knee in 52°C water for 15 seconds (noxious heat); (ii) pinching of 5 skin folds on the calf with forceps applied for 5 seconds at each site (pinch); (iii) subcutaneous injection of capsaicin (10 μl) into the lateral surface of the calf (capsaicin); (iv) subcutaneous injection (10 μl) of the vehicle used to deliver capsaicin (vehicle). Capsaicin was initially prepared at 1% by dissolving in 7% Tween 80, 20% ethanol in saline, and then diluted to 0.25%. Three of the mice were anaesthetised with urethane and used to investigate phosphorylation of ERK after intradermal injection of histamine (100μg/10μl) into the lateral calf. The success of the intradermal injection was assessed by the formation of a small bleb in the calf skin [50]. To avoid detecting phosphorylated ERK (pERK) that had resulted from the intradermal injection itself, in these cases, perfusion fixation was carried out 30 minutes after the stimulus. We have previously shown that intradermal injection of vehicle (PBS) causes ERK phosphorylation when animals are perfused with fixative 5 minutes after the injection. This presumably results from the noxious stimulus caused by insertion of the needle and distension of the skin [6]. In contrast, we found that injection of pruritogens, but not vehicle, resulted in pERK-immunoreactivity in neurons in laminae I-II when the mice were perfusion-fixed 30 mins after the injection. This is likely to reflect the relatively prolonged activation of these cells by pruritogens.

The L3 spinal segment, which contains the great majority of cells activated by these stimuli was cut into 60 μm thick transverse sections, which were reacted with antibodies against NeuN (mouse), GFP (chicken) and pERK. These markers were revealed with fluorescent secondary antibodies and sections were stained with DAPI. Sections that contained numerous pERK-positive neurons were initially selected, and these were scanned with the confocal microscope through the 40× oil-immersion lens, to generate z-stacks (2 μm z-separation) through the full thickness of the section so as to include the region that contained pERK cells. The z-stacks were analysed with Neurolucida. Initially, the outline of the grey matter was plotted, together with the ventral border of the GRP plexus (which corresponds approximately to the boundary between the inner and outer parts of lamina II). The mediolateral extent of the region that contained a high density of pERK cells was delineated by drawing two parallel lines that were orthogonal to the laminar boundaries. The channels corresponding to NeuN and DAPI were viewed, and the locations of all neurons that lay within this region were plotted onto the drawing. To avoid overcounting neurons, we only included them if at least part of the nucleus (stained with DAPI) was present in the first optical section of the z-series and excluded them if part of the nucleus was present in the last optical section [2,58]. The channel corresponding to pERK was then viewed, and the presence or absence of staining in each of the neurons in the sample was recorded. Finally, the GFP channel was viewed and all neurons that were GFP^+^ were identified on the drawing. As pERK^+^ cells were present at highest density in laminae I and IIo, we determined the proportion of all neurons that were located within this region and between the two parallel lines that were pERK-immunoreactive. We then determined the proportion of GFP^+^ neurons within this volume that were pERK-immunoreactive.

*Terminology*

For convenience, we refer to cells that expressed fluorescent proteins following intraspinal injections of AAVs coding for Cre-dependent forms of these proteins into the Tac1^Cre^ mouse line as "substance P (SP) cells". Similarly, we refer to cells that were GFP-positive in the GRP::eGFP mouse as "GRP cells", even though not all cells with GRP mRNA were GFP-positive in this line (see below).

*Statistics*

Two-way repeated measures ANOVA with *post hoc* Sidak test was used to determine whether there were significant differences in the proportions of neurons that were GFP^+^ in medial and lateral parts of the dorsal horn at L1 and L4 in the GRP::eGFP mice and a t test was used to compare medial and lateral counts from *in situ* hybridisation data. Differences in electrophysiological properties between the GRP and SP cells were compared using Mann-Whitney U or Wilcoxon signed-rank tests. Recorded neurons were classified as responsive to capsaicin or icilin by comparing the cumulative probability distribution of sEPSC/mEPSC interevent intervals with a 2-sample Kolmorgorov-Smirnov test. T tests were used to compare morphometric parameters from reconstructed axons and dendritic trees of GRP and SP populations. To determine whether there was a significant difference in the proportions of GRP^+^ and GRP- neurons in L5 that were retrogradely labelled from T13/L1, a contingency table was analysed with the Mantel–Haenszel test [44]. Breslow–Day testing for homogeneity of the odds ratio was conducted prior to computation of the Mantel–Haenszel odds ratio and 95% confidence intervals. The Mantel–Haenszel test was also used to determine whether the proportions of GRP^+^ and GRP-negative cells that showed pERK in response to noxious or pruritic stimuli differed significantly. Data are expressed as mean ± SEM, unless stated otherwise. P values of less than 0.05 were considered to be significant. Statistical tests were performed in Prism 7 (GraphPad) or SPSS (Version 22, IBM, for the Mantel-Haenszel test).

## Results

*Mediolateral distribution of GRP-eGFP cells*

During the course of experiments with the GRP::eGFP mouse, we noted that there was some variability in the number of GFP^+^ cells in the superficial dorsal horn in different mice. In addition, we observed a consistently lower number of GFP-positive cells in the medial part of the dorsal horn in caudal lumbar segments (especially L4 and L5) (Fig 1a,b). Because this region is innervated by glabrous skin of the hindpaw, we compared the proportion of neurons that were GFP^+^ in regions innervated by hairy and glabrous skin. This analysis was performed in the L4 segment (which receives input from both hairy and glabrous skin) and L1, which receives its cutaneous input only from hairy skin. The mean numbers of neurons counted per segment were 934 (882-1122) for L4 and 738 (702-808) for L1 (n=3 mice). Although the percentage of lamina I-II neurons that were GFP^+^ in L1 and in the lateral (hairy skin) part of L4 was very similar (12-14%), only 3% of those in the medial (glabrous skin) part of L4 expressed GFP (Fig 1c). A 2-way repeated measures ANOVA demonstrated a significant effect of lumbar location (*F*(1, 2) = 37.15, p = 0.026) and mediolateral location (*F*(1, 2) = 27.3, p = 0.035). The interaction between these factors was significant (*F*(1, 2) = 86.61, p = 0.011), and *post hoc* Sidak test shows a significant mediolateral difference at L4 (t(2) = 2.08, p = 0.0085) but not at L1 (t(2) = 15.24, p = 0.32).

The lack of GRP::eGFP cells in medial L4-5 could reflect the absence of cells that express GRP or a lack of GFP in some GRP-expressing neurons in the BAC transgenic line. To distinguish between these possibilities, we carried out *in situ* hybridisation histochemistry on tissue from GRP::eGFP mice. We identified a mean of 43.7 (35-54) GFP^+^ cells and 96.0 (90-107) GRP mRNA cells in sections from 3 mice and found that virtually all (98%) GFP^+^ cells had detectable GRP mRNA, whereas 44.5% (39-51%) of cells with the mRNA were GFP-positive. GRP mRNA^+^ cells were found throughout the mediolateral extent of the dorsal horn in the midlumbar region (Fig 1d) and the medial part of the L4 and L5 segments contained numerous GRP mRNA^+^/GFP-negative cells. We could not determine the proportion of neurons with GRP mRNA, due to the lack of a neuronal marker in these sections, but we noted that even though the lateral and medial parts (defined by the presence and absence of the intense GFP plexus) were approximately equal in size, GRP mRNA cells were more numerous in the lateral part (57-84 cells, mean 65.3 for the lateral part; 23-36 cells, mean 30.6 for the medial part; n = 3 mice). However, the extent of overlap between GRP mRNA and GFP was significantly different, depending on mediolateral location. In the medial part, only 30.5% (28-33%) of GRP mRNA-positive cells were GFP-positive, compared to 50.8% (46-56%) in the lateral part, and this difference was significant (t test, t(4) = 5.93 , p = 0.004). We may have underestimated the proportion of GRP mRNA cells that express GFP due to some loss of GFP signal resulting from the *in situ* hybridisation protocol [57]. However, these results suggest that while there may be fewer GRP mRNA-positive cells in the glabrous skin territory, lack of expression of GFP in GRP-expressing neurons is partially responsible for the low numbers of GFP cells seen in this region in the GRP::eGFP mouse.

Since we do not have a convenient way of identifying cells with GRP mRNA that did not express GFP, our subsequent analysis was restricted to the GFP-positive population. It should be borne in mind that these cells may differ functionally from the GFP-negative cells that contain GRP mRNA.

*Limited co-expression of GRP and SP*

We previously identified SP-expressing neurons by using immunocytochemistry to reveal PPTA, and reported that there was minimal overlap between PPTA-immunoreactive neurons and those that were GFP-positive in the GRP::eGFP mouse [23]. However, we also found that the PPTA antibody labelled fewer neurons than were seen following intraspinal injection of AAV.flex.tdTom into Tac1^Cre^ mice. It is therefore possible that we underestimated the degree of overlap between these populations. To better separate these populations, we carried out experiments on Tac1^Cre^;GRP::eGFP and Tac1^Cre^ mice that had received intraspinal injections of AAVs coding for either tdTom or eGFP (Fig 2). We first examined tissue from two Tac1^Cre^;GRP::eGFP mice that had been injected with AAV.flex.tdTom, and measured the extent of overlap between tdTom^+^ (SP cells) and GFP^+^ (GRP cells) in laminae I-II. We also stained for PKCγ, which is found in a different population of excitatory interneurons [26,57]. This analysis provided further evidence that the SP, GRP and PKCγ cells are largely separate populations (Fig 2a,b). The mean number of cells that were tdTom^+^, GFP^+^ and/or PKCγ^+^ in sections from the two mice was 356 (364, 347 in the 2 mice). Of these, 189 (199, 178) were tdTom-positive, 75 (80, 70) were GFP-positive and 110 (114, 105) were PKCγ-immunoreactive. As reported previously [24,26], we found some overlap between GRP-eGFP cells and PKCγ-immunoreactive cells (corresponding to 17% of the GFP cells and 11% of those with PKCγ), but minimal overlap between tdTom and PKCγ cells (corresponding to 2.1% of the tdTom cells and 3.6% of those with PKCγ). In addition, we saw very little overlap of the tdTom (SP) population with the GRP-GFP cells (1.3% of the GRP-GFP cells and 0.6% of the tdTom cells).

Because some GRP cells may not be detected in the GRP::eGFP mouse, we also compared the distribution of GRP mRNA with that of GFP in three Tac1^Cre^ mice that had been injected with AAV.flex.eGFP (Fig 2c). We identified a mean of 75.3 (68-85) GFP^+^ cells and 46.3 (44-49) GRP mRNA cells and found only 2.3 (2-3) double labelled cells (corresponding to 3.2% of the GFP population and 5.2% of the GRP mRNA cells).

In sections of cervical cord that had undergone double-labelling *in situ* hybridisation (Fig 3), we identified a mean of 678 cells with SP mRNA (range 561-783, n=3 mice) and 618 (483-715) cells with GRP mRNA. The mean number of cells in these sections that contained both SP and GRP mRNAs was 72 (range 54-85), and this corresponded to 10.7% (9.6-11.3%) of the cells with SP mRNA and 11.7% (10.9-12.9%) of those with GRP mRNA. A similar pattern was seen in the sections of lumbar cord (1 mouse): we identified 572 cells with SP mRNA, 497 cells with GRP mRNA and 60 of these cells had both mRNAs. In this case, cells with both mRNAs corresponded to 10.5% of the GRP+ cells and 12.2% of the SP+ cells. We conclude that although there is a limited overlap between SP and GRP cells, they are largely separate populations.

*Electrophysiological properties of GRP and SP cells*

As we did not observe any differences between male and female mice, all data presented are from a combination of both sexes. For the electrophysiological parts of the study, GRP cells were identified by GFP expression in slices from the GRP::eGFP mice, while SP cells were identified by expression of either GFP or tdTom in slices obtained from Tac1^Cre^ mice that had received intraspinal injections of AAVs coding for Cre-dependent forms of the corresponding fluorescent protein. Because GRP-eGFP cells were relatively infrequent in the medial part of the L4-5 segments, parasagittal slices through these segments were cut in such a way as to allow recordings from cells located in the more lateral parts of the segment.

The incidence of action potential firing patterns differed substantially between GRP and SP cells (Fig 4). GRP cells generally displayed transient (107/216, 49.5%) or single-spike (71/216, 32.9%) firing patterns, which were rarely seen in SP cells (3/101, 3% and 5/101, 5%, respectively). In contrast, the great majority of SP cells (80/101, 79.2%) exhibited the delayed firing pattern, which was very seldom seen in the GRP cells (6/216, 2.8%). Both cell populations contained a small proportion of tonic-firing cells (GRP: 18/216, 8.3%, SP: 8/101, 7.9%), together with a few cells that were classified as reluctant (GRP: 14/216, 6.5%, SP: 2/101, 2%), while 3 of the SP cells (3.0%) showed a long first inter-spike interval, and were classified as gap-firing [29].

We have shown that when Tac1^Cre^ mice received intraspinal injections of AAV.flex.eGFP, around 10% of the GFP^+^ cells are Pax2^+^ (inhibitory) neurons, and these are therefore likely to have been included among the neurons recorded in these experiments. Previous studies have shown that delayed, gap and reluctant firing patterns, which are thought to result from the presence of A-type potassium currents, are particularly associated with excitatory interneurons in lamina II [29,49,73]. For all subsequent electrophysiological and pharmacological parts of the study, we therefore restricted the analysis to the 84% of SP cells (85/101) that showed delayed, gap or reluctant firing. This approach reduced the risk that recordings were from inhibitory SP neurons.

Consistent with the much higher proportion of SP cells that showed gap or delayed firing, the latency between the onset of the current injection and the first action potential at rheobase was significantly greater for the SP cells (595.0 ± 28.26 ms) compared to the GRP cells (137.1 ± 6.2 ms) (Mann-Whitney, *U* = 504, p < 0.001, n = 155 GRP cells, 80 SP cells). The rheobase of SP cells was 46.45 ± 2.15 pA, and this was significantly larger than that of GRP cells, 18.30 ± 1.07 pA (Mann-Whitney, *U* = 1275, p < 0.001, n = 155 GRP cells, 80 SP cells).

The subthreshold I-V relationship was determined by voltage clamping the cells at ‑60 mV and applying 100 ms voltage steps from ‑70 to ‑50 mV in 2.5 mV increments. The resting membrane potential, as calculated from the I-V relationship for individual cells, was ‑52.89 ± 0.78 mV for GRP cells and -55.77 ± 0.90 mV for SP cells, with the GRP cells having a significantly more depolarised resting membrane potential (Mann-Whitney, *U* = 7758, p = 0.0074, n = 230 GRP cells, 84 SP cells). The capacitance of GRP cells was significantly smaller than that of SP cells (5.12 ± 0.11 vs. 7.07 ± 0.33 pF; Mann-Whitney, *U* = 5904, p < 0.001, n = 232 GRP cells, 85 SP cells), with GRP cells also displaying a greater input resistance (1588 ± 85 vs. 836 ± 52 MΩ; Mann-Whitney, *U* = 5315, p < 0.001, n = 232 GRP cells, 84 SP cells).

Almost all SP neurons tested (60/64, 93.8%, Fig 5a,b) displayed a rapid I_A_ current (I_Ar_), and most showed a hyperpolarisation-activated current (I_h_) (42/64, 65.6%), with many exhibiting both I_Ar_ and I_h_ (38/64, 59.4%). While I_Ar_ was the most commonly seen subthreshold current in GRP cells (65/159, 40.9%, Fig 5a,b), the incidence was lower than in SP cells, and the amplitude was significantly smaller (54.9 ± 4.6 vs. 263.3 ± 16.5 pA, Mann-Whitney, *U* = 64, p < 0.0001, Fig 5c). Many GRP cells displayed I_h_ (59/159, 37.3%), with some cells exhibiting both I_h_ and I_Ar_ (13/159, 8.2%). The amplitude of I_h_ in GRP cells was significantly larger than that recorded in SP cells (‑17.5 ± 1.1 vs. -13.9 ± 1.0 pA, Mann-Whitney, *U* = 911.5, p = 0.024, Fig 5d). Although not detected in the SP cells, slow I_A_ current (I_As_) (41/159, 25.8%) and low threshold Ca currents (I_Ca,T_) (53/159, 33.3%) were recorded in some GRP cells. Some GRP cells that showed I_As_ also exhibited I_h_ (36/159, 22.6%) or I_h_ and I_Ca,T_ (1/159, 0.6%), and I_Ca,T_ was found to overlap with I_Ar_ (5/159, 3.1%), and with I_h_ (3/159, 1.9%).

*Excitatory inputs to GRP and SP cells*

As stated above, analysis of SP cells was restricted to those with delayed, gap or reluctant firing patterns. SP cells showed a higher frequency of both sEPSCs and mEPSCs (6.04 ± 0.96 Hz sEPSCs, 3.24 ± 0.67 Hz mEPSCs, n = 27 and 11, respectively) than GRP cells (0.2 ± 0.05 Hz sEPSCs, 0.02 ± 0.01 Hz mEPSCs, n = 120 and 32, respectively) and these differences were both highly significant (sEPSC, Mann-Whitney, *U* = 68, p < 0.0001; mEPSC, Mann-Whitney, *U* = 0, p < 0.0001; Fig 6). For 11 of the SP cells, we were able to compare sEPSC frequency with mEPSC frequency in the same cell. The sEPSC frequency for these cells (5.39 ± 0.93 Hz) was higher than the mEPSC frequency (3.24 ± 0.67 Hz), and this difference was highly significant (Wilcoxon signed-rank test; *W* = 66, p = 0.0036). This finding suggests that the SP cells receive excitatory synaptic input from neurons that were spontaneously firing action potentials in the slice.

Capsaicin caused a leftwards shift in the distribution of mEPSC inter-event intervals in 1 out of 11 GRP and 4 out of 9 SP cells (Fig 6c,d). Capsaicin increased mEPSC frequency from 0.04 to 0.17 Hz in the single responsive GRP cell, and from 3.58 ± 0.91 to 8.78 ± 1.79 Hz in the responsive SP cells (example traces shown in Fig 6dii). When sEPSCs were recorded, 4 out of 16 GRP and 3 out of 5 SP cells were found to be sensitive to capsaicin (Fig 6c). For those cells that were classed as sensitive, capsaicin increased sEPSC frequency from 0.36 ± 0.10 to 3.58 ± 2.09 Hz in GRP cells, and 1.64 ± 0.16 to 3.29 ± 0.30 Hz in SP cells (example traces shown in Fig 6di). Application of icilin did not increase mEPSC frequency in any of the GRP (n = 7) or SP cells (n = 7) that were tested (Fig 6c,diii). Since TRPM8 and TRPV1 expression in the dorsal horn are thought to be restricted to primary afferents, these data suggest that neither GRP nor SP cells receive monosynaptic input from TRPM8-espressing afferents. These findings also indicate that both cell types receive input from TRPV1-expressing afferents, and that this includes monosynaptic input, although this is considerably more prevalent in SP cells than GRP cells.

*Responses of GRP and SP cells to neuromodulators*

To investigate the effect of neuromodulators (noradrenaline (NA), 5-HT and opioids) on GRP and SP cells, we bath applied different agonists (Fig 7). DAMGO (3 μM) caused an outward current (7.36 ± 0.99 pA) in all but one of the GRP cells tested (14/15); none of the 7 SP cells tested were responsive. None of the GRP cells responded to the KOR agonist, U69593 (1 μM), and this caused an outward current (7.23 pA) in only 1 out of the 8 SP cells tested. The DOR agonist [D-Ala^2^]-Deltorphin II (1 μM) was tested on 7 GRP cells and 8 SP cells, but had no effect on any of these. Application of NA (20 μM) caused an outward current in the majority (7 out of 9) of the SP cells (15.59 ± 2.54 pA), but in only 1 out of 6 GRP cells (8.86 pA). None of the 6 GRP cells tested responded to 5-HT (10 or 20 μM), whereas all 8 SP cells tested displayed an outward current (19.22 ± 2.55 pA). These findings demonstrate that GRP and SP cells differ in their response profiles to the monoamines and MOR agonist, while few or none of these cells respond to KOR or DOR agonists. Specifically, most putative excitatory SP cells are hyperpolarised by both NA and 5-HT, but not by any of the opioid agonists. In contrast, most GRP cells do not respond to NA, 5-HT, DOR or KOR agonists, but are hyperpolarised by MOR agonists.

## *Morphological properties of GRP and SP cells*

Morphological analysis was carried out on 45 GRP cells that underwent whole cell recording, and from a total of 43 SP cells (31 from perfusion-fixed Brainbow tissue and 12 from electro­physiological experiments). In the tissue from Tac1^Cre^ mice injected with Brainbow AAVs, initial scans revealed that the distribution and density of labelled cells was generally consistent with that seen following injection of AAV.flex.tdTom [23] (Fig 8a). However, although ~10% of the labelled cells in our previous study were Pax2-immunoreactive, only 1 of 100 Brainbow-labelled neurons examined was Pax2-positive, even though numerous Pax2^+^ nuclei were present within the dorsal horn (Fig 8b-d). Clearly this strategy selectively targets the excitatory SP-expressing neurons. All of the 31 reconstructed SP neurons had nuclei that were Pax2-negative.

Preliminary observation of the Neurolucida reconstructions suggested that the two populations differed in terms of somatodendritic morphology. Although the GRP cells were morphologically heterogeneous, they generally had dendritic trees that were considerably longer in the rostrocaudal axis than in dorsoventral or mediolateral axes, but were shorter than those of islet cells. At least some of these cells could therefore be classed as central cells [22,71,73]. In contrast, Fig 8e shows that many of the SP cells reconstructed from the Brainbow experiments resembled radial cells, with relatively numerous primary dendrites that did not extend far from the cell body, and compact dendritic trees. This initial observation was confirmed by principal component analysis and subsequent cluster analysis of dendritic morphometric parameters extracted from the Neurolucida drawings. These parameters are listed in detail in Table 5 of reference 18. A scree test revealed that 5 principal components accounted for 80% of the total variance in the dataset, and these were therefore used for cluster analysis. This separated the reconstructed neurons into two distinct clusters, one of which (n = 30) consisted entirely of SP cells, and the other (n = 46) of which included all of the GRP cells, together with one SP cell (Fig 8f).

Because the two populations were obtained from different types of experiment, we were concerned that this might have influenced the clustering results. For example, due to the difficulty of following fine distal dendrites, we may have underestimated the sizes of dendritic trees in the Brainbow material. We therefore looked for factors that correlated well with the principal components distinguishing the clusters, and found that the number of primary dendrites was a major factor. We therefore compared the number of primary dendrites between the two populations, and found a highly significant difference (Table 3). We also found that both the dorsoventral (DV) extent of dendritic trees, and the ratio of DV to rostrocaudal (RC) extent [71] differed significantly. The SP cells had more primary dendrites and a lower RC:DV ratio (Table 3), both of which are consistent with radial cell morphology. Although we did not analyse the dendritic trees of the recorded SP cells in detail, we noted that these resembled those of the SP neurons seen in the Brainbow tissue (Fig 9c,d). These cells also gave rise to numerous primary dendrites (mean 6.9 ± 1.5 SD), which is similar to the number of primary dendrites on the reconstructed Brainbow neurons (7.4 ± 1.4, Table 3).

Because of the difficulty of following axons belonging to individual neurons in Brainbow material, axonal morphology for the SP cells was only analysed on those that had undergone whole-cell recording. For many of the GRP cells, we found that although a well-filled axon could be seen emerging from the soma or a primary dendrite, the axon rapidly turned either medially or laterally and left the slice either without branching (n = 14) or after giving rise to a small arbor (n = 9). For this reason axonal morphology was only analysed on 22 of the 45 GRP cells. Examples of axonal arbors are illustrated in Fig 9. The total length of axon that was reconstructed was significantly greater for the GRP than for the SP cells (t test, t(32) = 3.68, p = 0.0008; Fig 9e). However, in both cases 95% of the axonal length remained in lamina II, with only a small amount in laminae I or III (2% and 4%, respectively for GRP cells, 4% and 1%, respectively for SP cells).

*Lack of long propriospinal projections of GRP cells*

The finding that the axons of recorded GRP cells often turned medially or laterally raised the possibility that these had propriospinal projections [7,25], and we therefore addressed this in the retrograde labelling experiments. The CTb injection sites in the T13 segment in these experiments included the whole of laminae I-V of the right dorsal horn as well as the lateral spinal nucleus (Fig 10a). The pattern of retrograde labelling with CTb in the L5 segment was very similar to that described previously [25]. There were numerous CTb-labelled neurons evenly distributed throughout the mediolateral extent of the superficial dorsal horn, as well as in deeper laminae (Fig 10b). Few retrogradely labelled cells were seen on the contralateral (left) side. The mean number of lamina I-II neurons included in the disector sample in the four mice was 589 (range 547-620), and 26.6% were CTb-immunoreactive (range 22.1-29.6%), indicating that at least a quarter of superficial dorsal horn neurons in L5 have axons that extend rostrally for 5 segments.

Cells that were positive for eGFP in the L5 segment accounted for 10.3% (7.6-12.6%) of all lamina I-II neurons, which is similar to our previous estimate (11%) [24]. Very few of the GFP^+^ cells, 3.5% (2.6-4.7%) were CTb-immunoreactive, while GFP-immunoreactive cells accounted for only for 1.3% (1.1-1.5%) of the CTb-labelled neurons in laminae I-II (Fig 10c,d). This difference in proportion was highly significant (Mantel-Haenszel test, χ^2^(1) = 73.0, p < 0.0001), with an odds ratio for a GRP cell to be retrogradely labelled calculated as 0.08 (0.04-0.17 95% confidence interval, Breslow-Day Significance; p = 0.944). We conclude that GRP-expressing cells are significantly under-represented among the superficial dorsal horn neurons that have long ascending propriospinal axons.

To test whether GRP cells give rise to short intersegmental connections, we also analysed sections from the L2 segment in 2 of the mice. We found that the proportion of all neurons in laminae I-II that were retrogradely-labelled with CTb from the T13 injection sites was 58% (range 55.9-60.1%), and that among GRP-eGFP cells the proportion that were CTb-labelled was 15.1% (range 13-17.1%). This indicates that a few of the GRP cells have axons that extend for at least two segments rostrally.

*Responses of GRP cells to noxious and pruritic stimuli*

Noxious and pruritic stimuli induced pERK in cells of the ipsilateral dorsal horn, mainly in the superficial laminae with a mediolateral extent that reflected the somatotopic location of a stimulus delivered to the lateral calf [6,23]. Few, if any, pERK cells were located on the contralateral side, or in the dorsal horn of mice that received vehicle injection.

First we determined the proportion of all neurons that were pERK positive following stimulation with histamine, capsaicin, pinch and noxious heat. The percentage of all neurons within the activated zone in laminae I-II that were pERK-immunoreactive varied between 23 and 37% depending on the stimulus (Table 4). We then compared the proportions of GFP^+^ and GFP-negative cells that showed pERK following the different types of noxious and pruritic stimulus. We found that in all cases, the GFP^+^ cells were significantly under-represented compared to GRP-negative neurons (Table 4). Examples of pERK staining in the GRP-eGFP mice are shown in Fig 11.

## Discussion

Our main findings are that SP- and GRP-expressing cells form largely non-overlapping populations among the excitatory interneurons in laminae I-II, and that these differ significantly in morphology, firing patterns, EPSC frequency and responses to neuromodulators. Comparison with previous data suggests that they also differ in responses to noxious and pruritic stimuli, and their contribution to propriospinal projections [23,25]. These findings are summarised in Fig 12.

*The function of GRP-expressing interneurons*

It is well established that GRP-expressing neurons have a major role in itch [46]. Sun et al [60] recently proposed that these cells respond weakly to pruritic stimuli and strongly to painful stimuli, but that when strongly activated they suppress pain through feed-forward inhibition, forming a "leaky gate". We previously reported that GRP cells rarely show pERK or Fos following intradermal chloroquine [6], and Sun et al suggested that this was because of their weak activation by pruriceptors. However, we show here that these cells very seldom develop pERK following noxious stimuli. Interestingly, Sun et al [60] report that capsaicin applied to DRG generated mean firing rates of <1 Hz in GRP cells, and if this is similar to their response to natural noxious stimuli, it may be inadequate to phosphorylate ERK. It has been reported that GRP cells are innervated by various types of primary afferent, including TRPV1-expressing nociceptors [60]. However, our finding that capsaicin increased mEPSC and sEPSC frequency in only 1/11 and 4/16 GRP neurons, respectively, suggests that TRPV1-expressing afferents seldom directly innervate GRP cells, although they do provide polysynaptic input to some of them. The potential involvement of GRP cells in nociceptive processing therefore remains to be established.

At first sight, our finding that GRP cells were inhibited by DAMGO is surprising, because MOR agonists can cause itching [63]. However, it has been suggested that this is mediated by MOR1D-GRPR heterodimers on GRPR neurons [39]. Since these cells are thought to be located downstream of GRP cells in the spinal itch pathway, activation of the GRPR cells by morphine acting on these heterodimers would presumably over-ride its inhibitory action on the GRP cells. The expression of MORs by GRP neurons suggests that endogenous release of MOR agonists (e.g. endomorphin-2) from nociceptors will inhibit activity in the itch pathway, contributing to suppression of itch by noxious stimuli. Although KOR agonists are anti-pruritic, they are thought to act at the level of the GRPR cells [30], consistent with the lack of response of GRP cells shown here. The finding that neither GRP nor SP cells responded to DOR agonist is consistent with the recent report that MOR and DOR are expressed by largely separate populations, with DOR-expressing cells being concentrated in lamina IIi [68]. It should be noted that in addition to their action on cell bodies and dendrites of dorsal horn neurons, opioid peptides can also act on axon terminals to modify synaptic transmission. Little is known about the role of monoaminergic systems in itch, although tricyclic antidepressants, which potentiate monoamine transmission, are used to treat pruritus [37]. The lack of effect of noradrenaline and 5-HT on GRP cells suggests that they act elsewhere in the itch pathway.

We found that there were consistently fewer GRP-eGFP cells in glabrous skin territory and *in situ* hybridisation revealed that this was not due solely to lack of GRP-expressing neurons in this region. This variability in GFP expression presumably reflects transcriptional heterogeneity between GRP cells innervated from hairy and glabrous skin, and may be related to differences in itch sensation from these two skin types [67]. As the GRP cells are relatively numerous, they presumably correspond to one or more of the populations identified by Grudt and Perl [22]. Although many excitatory interneurons in lamina II show delayed/gap/reluctant firing [29,49,73], this was seldom seen for GRP cells, which generally showed transient or single-spike firing. Since the GRP cells had dendritic trees that were moderately elongated along the rostrocaudal axis, many of them are likely to correspond to the "transient central" population of Grudt and Perl. Interestingly, transient central cells have been implicated in a circuit linking low-threshold mechanoreceptive afferents to lamina I projection neurons, which is thought to contribute to tactile allodynia [40,41]. It will therefore be important to determine whether the cells in this putative circuit include GRP-expressing neurons, and how this relates to their proposed role in itch.

*SP-expressing excitatory interneurons are radial cells*

Unlike GRP cells, most SP neurons (79.2%) showed delayed firing, with a few having gap or reluctant patterns. As ~10% of the cells labelled with this strategy are inhibitory interneurons [23], which seldom show these firing patterns, the proportion of excitatory SP interneurons with delayed/gap/reluctant-firing is presumably even higher. Cluster analysis revealed a clear difference in dendritic morphology between SP and GRP cells. SP cells had significantly more primary dendrites, characteristic of radial cells. Alba-Delgado et al [3] reported that some PKCγ-immunoreactive cells in lamina II of the medullary dorsal horn have radial morphology. However, unlike the radial cells described here and in previous studies [22,73], PKCγ cells do not show delayed firing [3,40]. Among other differences, SP cells showed higher mEPSC frequencies than GRP cells, suggesting that they receive more excitatory synapses. Consistent with radial cells receiving both Aδ and C inputs [22,71], 4 of 9 SP neurons showed a TRPV1-dependent, capsaicin-evoked increase in mEPSC frequency.

Little is known about the neuronal circuits engaged by radial cells. We previously reported that many excitatory SP neurons are activated by noxious or pruritic stimuli, and that many have long propriospinal axons targeting the lateral spinal nucleus (LSN) [23,25]. Interestingly, Grudt and Perl [22] reported that most radial cells had axons that ran rostrally and/or caudally in the dorsolateral fasciculus, consistent with our finding of propriospinal projections from these cells to the LSN. The SP cells may therefore contribute to the large cutaneous receptive fields characteristic of LSN neurons in inflammatory pain states [55]. Conceivably, this circuit serves a protective role, since extension of pain beyond the site of damage would limit use of an injured limb during recovery. The scarcity of GRP cells among propriospinal interneurons probably reflects the different behavioural requirement for itch, where spatial acuity is needed to remove the underlying cause by scratching or biting.

Most SP cells were inhibited by noradrenaline and 5-HT, and similar results have been found for radial cells in rat [42,73]. Inhibition of these cells from the brainstem may therefore contribute to the anti-nociceptive actions of descending monoaminergic pathways. Glycinergic neurons in deeper laminae of the dorsal horn provide another source of inhibition for radial cells [71], and it has been proposed that reduction in glycinergic input to radial cells contributes to neuropathic pain [33].

Together, these findings suggest that the excitatory SP interneurons correspond to the radial cells identified by Grudt and Perl [22], and that they play an important role in pain mechanisms.

*Excitatory interneuron populations*

Two recent transcriptomic studies [27,53] have defined neurochemically distinct populations of dorsal horn excitatory neurons. Although both studies identified the SP, NKB and neurotensin populations that we previously described [25,66], only one recognised the GRP cells as a distinct population [53]. In that study, the GRP (but not SP) population was reported to express the MOR gene (OPMR1), consistent with our finding that GRP (but not SP) cells possess functional MORs. Haring et al [27] did not detect a specific population of GRP-expressing cells, and reported considerable overlap between GRP and Tac1 mRNAs. However, our *in situ* hybridisation data clearly shows that GRP-expressing cells in lamina II differ from those that express SP, suggesting that they are indeed a distinct neurochemical population.

Vertical cells constitute a well-defined class of excitatory interneurons, with cell bodies in lamina IIo and ventrally-directed dendrites [22,43,49,71-73]. They are presynaptic to lamina I projection neurons [12,41], and have been implicated in transmitting low-threshold mechanoreceptive information to the projection cells, thus contributing to tactile allodynia [40]. The present results show that neither SP nor GRP populations include vertical cells, and since the neurotensin and NKB neurons are found in laminae IIi-III, these are also unlikely to include vertical cells. Finding the neurochemical signature of vertical cells is therefore a high priority. In this regard, we recently identified a cluster of dynorphin-expressing excitatory interneurons with vertical cell morphology, however, these are largely restricted to glabrous skin territory [30]. Two additional classes of excitatory interneuron identified in transcriptomic studies consist of cells that express neuropeptide FF (NPFF) or the neuromedin 2 receptor (NMUR2) [27,53], and in both cases, the cells are concentrated in lamina IIo [11,27]. It will therefore be important to test whether either of these populations correspond to vertical cells, as this would allow selective genetic targeting of these cells to investigate their role in spinal pain circuits. Since both SP and GRP populations have axons that arborise mainly in lamina II, vertical cells may also provide the route through which these cells can activate lamina I projection neurons. Defining the circuits that link the different populations of excitatory interneurons will therefore be of considerable importance.

**Conflicts of interest**

The authors report no conflicts of interest.

**Acknowledgements**

We are grateful to Robert Kerr and Christine Watt for expert technical help and to Drs Kieran Boyle, Mark Hoon and Toshiharu Yasaka for helpful discussion. Financial support from the Wellcome Trust (grant 102645) and the Biotechnology and Biological Sciences Research Council (grant N006119/1) is gratefully acknowledged.

**Supplemental video content**

A video abstract associated with this article can be found at <http://links.lww.com/PAIN/A665>.

FIGURE LEGENDS

Fig. 1 Differences in the mediolateral distribution of GFP and GRP mRNA in GRP::eGFP mice. **a**,**b**: Immunostaining for GFP (green) and VGLUT3 (magenta) in the dorsal horn of GRP::eGFP mice in the L1 and L4 segments. A dense plexus of VGLUT3 staining is present in the inner part of lamina II, and this corresponds to the central terminals of C-LTMR afferents, which are associated with hairy skin. The plexus is evenly distributed across the L1 dorsal horn, but is restricted to the lateral part in L4. The medial region, where the plexus is absent, is innervated from glabrous skin of the hindpaw. Note that GFP^+^ cells are largely restricted to those regions with hairy skin input. These images are projections from z-stacks through the full thickness of 60 μm sections. **c**: Quantification of GFP^+^ cells as a proportion of all neurons in the medial and lateral halves of the dorsal horn in L1 and the glabrous (medial) and hairy (lateral) territories in L4 (mean ± SD). **d**: *in situ* hybridisation histochemistry on the L4 segment reveals that GRP mRNA (magenta) is present in many cells in the medial half of the dorsal horn, even though there are very few GFP^+^ (green) cells. The dashed line represents the grey-white matter border. Scale bars: **a**,**b** = 200 μm, **d** = 50 μm.

Fig. 2 Distribution of cells expressing substance P (SP), GRP and PKCγ. **a**: A confocal image showing part of a transverse section through the dorsal horn of the L3 segment from a Tac1^Cre^;GRP::eGFP mouse that had received an intraspinal injection of AAV.flex.tdTom 2 weeks previously. The section has been immunostained for GFP (green) and PKCγ (blue), while the native fluorescence of tdTom is shown in red. Cells containing each of these markers form largely separate populations. Although these overlap, the tdTom^+^ (SP-expressing) cells are generally dorsal, and the PKCγ cells ventral, to the GRP-eGFP cells. **b**: Pie chart showing the relative sizes and extent of overlap of these populations. **c**: lack of coexpression of GRP and SP is further supported by the finding that there is virtually no overlap between GRP mRNA (magenta) and GFP (green) in the L3 segment of a Tac1^Cre^ mouse that had received an intraspinal injection of AAV.flex.eGFP. Scale bars: **a**,**b** = 100 μm

Fig. 3 Limited co-existence of mRNAs for SP and GRP in a section of cervical dorsal horn that had been reacted with a double-label *in situ* hybridisation method. **a**: SP mRNA is shown in green. **b**: the same field scanned to reveal GRP mRNA (magenta). **c**: a merged image shows that most cells that are labelled contain only one of the mRNA types. However, some cells are double-labelled, and two of these are indicated with arrows. Scale bar = 50 μm.

Fig. 4 Action potential firing patterns in GRP and SP cells. GRP cells were identified by the presence of GFP in slices taken from GRP::eGFP mice. SP cells were identified by expression of either GFP or tdTom in Tac1^Cre^ mice that had received injections of AAV coding for Cre-dependent forms of the corresponding fluorescent protein. **a**: In response to a suprathreshold current injection (1s), most GRP cells displayed transient (107/216, 49.5%) or single spike (71/216, 32.9%) firing, with a smaller proportion of cells showing tonic (18/216 , 8.3%), reluctant (14/216, 6.5%) or delayed (6/216, 2.8%) firing (**ai**). The majority of SP cells exhibited delayed firing (80/101, 79.2%), with smaller proportions displaying tonic (8/101, 7.9%), single spike (5/101, 5%), transient (3/101, 3%), gap (3/101, 3%) or reluctant (2/101, 2%) firing patterns (**aii**). **b**: examples of transient, single spike and reluctant firing GRP cells, and delayed and tonic firing SP cells.

Fig. 5 Subthreshold voltage activated currents in GRP and SP cells, which were identified as described in the legend for Fig 4. **a**: Subthreshold currents were examined in GRP and SP cells using a voltage step protocol that hyperpolarised cells from ‑60 to -90 mV for 1 s and then to -40 mV for 200 ms (lower left trace). The responses to this protocol were classified as rapid (I_Ar_) or slow (I_As_) A-type potassium currents, hyperpolarisation activated currents (I_h_) or low threshold calcium currents (I_Ca,T_). Examples from both GRP (green) and SP (red) cells show an average of 5 traces. The example of I_h_ for the GRP cell (dashed outline) is shown at two different y-axis scales. **b**: Almost all SP cells displayed I_Ar_ (60/64, 93.8%) and most had I_h_ (42/64, 65.6%), with many cells exhibiting both (38/64, 59.4%). I_Ar_ was the most commonly seen current in GRP cells (65/159, 40.9%), with fewer cells showing I_h_ (59/159, 37.3%), I_As_ (41/159, 25.8%) or I_Ca,T_ (53/159, 33.3%). **c**: The amplitude of I_Ar_ was significantly greater in SP cells (***c***, 263.3 ± 16.5 vs. 58.6 ± 4.6 pA), **** p < 0.0001, Mann-Whitney U test. **d**: I_h_ amplitude was significantly larger in GRP cells (***d***, -17.5 ± 1.1 vs. -13.9 ± 1.0 pA). * p = 0.024, Mann-Whitney U test.

Fig. 6 Excitatory inputs to GRP and SP cells, which were identified as described in the legend for Fig 4. sEPSCs (**ai**) and mEPSCs (**bi**) were recorded in GRP and SP cells. SP cells were found to receive greater excitatory drive, having a higher frequency of both types of excitatory event (6.04 ± 0.96 Hz sEPSCs, **aii**, 3.24 ± 0.67 Hz mEPSCs, **bii**, n = 27 and 11, respectively) than GRP cells (0.2 ± 0.05 Hz sEPSCs, **aii**, 0.02 ± 0.01 Hz mEPSCs, **bii**, n = 120 and 32, respectively). These differences were both highly significant (**** p < 0.0001, Mann-Whitney U test). **c**,**d**: Primary afferent input to GRP and SP cells was assessed by recording sEPSCs and mEPSCs in response to the TRP channel agonists capsaicin (TRPV1, **di-ii**) and icilin (TRPM8, mEPSCs only, **diii**). **c**: When mEPSCs were recorded, capsaicin caused a significant leftwards shift in the distribution of inter-event intervals in 1 out of 11 GRP cells and 4 out of 9 SP cells; when sEPSCs were recorded 4 out of 16 GRP cells and 3 out of 5 SP cells were found to be capsaicin-sensitive. Icilin did not increase mEPSC frequency in any of the GRP (n = 7) or SP cells (n = 7) tested.

Fig 7 Responses of GRP and SP cells to neuromodulators. Cells were identified as described in the legend for Fig 4. **a**: The proportions of tested GRP and SP cells that responded to various neuromodulators are shown. **bi**: the MOR agonist DAMGO caused an outward current in most GRP cells (14/15) but was without effect in SP cells. **bii**: No GRP cells and only 1 out of 7 SP cells responded to the KOR agonist, U69593. **biii**: No cells of either type responded to the DOR agonist, [D-Ala^2^]-Deltorphin II. **biv**: noradrenaline (NA) elicited a response in most SP cells (7/9), but only one out of 6 GRP cells. **b**: 5-HT evoked an outward current in all 8 SP cells, while the 6 GRP cells tested were unresponsive.

Fig. 8 Morphology of SP and GRP cells. **a:** The labelling that results from injection of AAV Brainbow into the dorsal horn of a Tac1^Cre^ mouse. This field shows part of lamina II in a projected image of 38 optical sections at 0.5 μm z-separation. Numerous neuronal cell bodies are visible (some marked with asterisks), and these show different hues, resulting from differential expression of blue fluorescent protein (BFP), teal fluorescent protein (TFP) and mCherry. Note that apart from some cytoplasmic staining for mCherry, the labeling is largely restricted to the plasma membrane and therefore outlines the cells. **b**-**d** show a projection of 5 optical sections at 1 μm z-separation from a Tac1^Cre^ mouse injected with AAV Brainbow. This has been scanned to reveal the fluorescent proteins (shown in **b** and **d**) and Pax2 (**c**,**d**). Although there are Pax2-positive cells in this field (some marked with arrows), none of these correspond to the Brainbow-labelled cells (arrowheads). **e**: Neurolucida reconstructions of the cell bodies and dendritic trees of representative SP and GRP cells. Note that the SP cells were obtained from Brainbow experiments, while the GRP cells were from electrophysiological recordings in the GRP::eGFP mice. **f**: Hierarchical cluster analysis (Ward's method) based on morphometric dendritic parameters results in almost complete separation of the SP and GRP cells, with only 1 out of 31 SP cells appearing in the lower cluster, which contains all 45 of the GRP cells. Scale bars: **a** = 20 μm, **b**-**d** = 20 μm, **e** = 50 μm.

Fig. 9 Morphology and laminar distribution of axons of GRP and SP cells. Cells were reconstructed following electrophysiological recordings, during which they were identified as described in the legend for Fig 4. **a**-**d**: Typical examples of Neurolucida reconstructions of GRP cells (**a**-**b**) and SP cells (**c**-**d**). Cell bodies and dendrites are shown in blue and axons in red (axonal boutons are not shown). In each drawing, the solid line indicates the grey-white border, while dashed lines represent the boundaries between laminae I/IIo, IIo/IIi and IIi/III. Note that the axons of the cells predominantly arborise in lamina II and rarely enter lamina I. D, dorsal; RC, rostrocaudal; V, ventral. Scale bar = 100 μm. The total measured axonal length in each population is shown (**e**) with the total length of axon being significantly greater in the GRP cells (t test P < 0.001).

Fig. 10 Retrograde labelling of L5 neurons from the T13 segment in a GRP::eGFP mouse. **a**: The injection site in the T13 segment of one of the mice. The CTb reaction product fills the entire dorsal horn and part of the ventral horn on the right side, with limited spread onto the contralateral side. **b**: A projected confocal image (17 optical sections at 2 μm z-spacing) showing part of the ipsilateral dorsal horn from the L5 segment of the same animal. Numerous NeuN^+^ (neuronal) profiles are visible (blue), and many of these are labelled with CTb (red) that has been retrogradely transported from the T13 injection site. The solid line indicates the dorsal edge of the dorsal horn and the dashed line the lamina II-III border. **c**,**d**: A single confocal image showing a higher magnification view from the region in the box in **d**. This field contains 2 GFP^+^ (green) cells (arrows), which are not CTb-labelled, and these are surrounded by several GFP-negative CTb-labelled neurons, some of which are marked with arrowheads. Scale bars: **a**, 500 μm; **b**, 50 μm; **c** and **d**, 20 μm.

Fig. 11 pERK immunoreactivity in GRP::eGFP mice following noxious or pruritic stimulation. **a**-**c**, **d**-**f**, **g**-**i** and **j**-**l** are representative fields from mice that were stimulated with histamine, pinch, noxious heat or capsaicin, respectively. In each case, the same field from the superficial dorsal horn is shown immunostained for pERK (magenta), GFP (green), together with a merged image. Each stimulus resulted in numerous pERK^+^ neurons, although GFP labelled cells were rarely pERK-immunoreactive. Each set of images shows GFP^+^ cells (some indicated with arrowheads), which are not immunostained for pERK, but are surrounded by pERK^+^ neurons. In each case, one of the pERK^+^ neurons is indicated with an arrow. Images are projections of 2 (a-c, j-l) or 3 (d-i) confocal optical sections at 2 μm z-spacing. Scale bar = 20 μm.

Fig. 12 A diagram showing major differences that were detected between excitatory interneurons that express substance P (SP) and gastrin-releasing peptide (GRP). One cell of each type is represented on a transverse section of the dorsal horn, with dashed black lines indicating the borders of lamina II. The SP cells have more primary dendrites than the GRP cells, and typically show radial morphology, whereas the GRP cells were either central or unclassified cells. Note that these definitions are based on the somatodendritic morphology as seen in sagittal sections. Axons of both types (indicated with dashed lines of the corresponding colour) arborise locally in lamina II, while the SP cells also project to the lateral spinal nucleus (LSN), giving rise to propriospinal axons within this nucleus. SP cells generally responded (with outward current) to the monoamines noradrenaline (NA) and 5-hydroxytryptamine (5-HT), whereas the GRP cells responded with outward current to μ-opioid agonist. Around half of the SP cells showed an increase in mEPSC frequency in response to bath-applied capsaicin, indicating monosynaptic input from TRPV1-expressing primary afferents. However, this was only seen for 1 of 11 GRP cells tested, suggesting that these cells are seldom innervated directly by these afferents. The firing patterns in response to injected depolarising current pulses also differed, with most SP cells showing delayed firing, and GRP cells generally having transient or single-spike patterns.

## REFERENCES

[1] Abraira VE, Ginty DD. The sensory neurons of touch. Neuron 2013;79(4):618-639.

[2] Al-Khater KM, Kerr R, Todd AJ. A quantitative study of spinothalamic neurons in laminae I, III, and IV in lumbar and cervical segments of the rat spinal cord. J Comp Neurol 2008;511(1):1-18.

[3] Alba-Delgado C, El Khoueiry C, Peirs C, Dallel R, Artola A, Antri M. Subpopulations of PKCgamma interneurons within the medullary dorsal horn revealed by electrophysiologic and morphologic approach. Pain 2015;156(9):1714-1728.

[4] Atasoy D, Aponte Y, Su HH, Sternson SM. A FLEX switch targets Channelrhodopsin-2 to multiple cell types for imaging and long-range circuit mapping. J Neurosci 2008;28(28):7025-7030.

[5] Balachandar A, Prescott SA. Origin of heterogeneous spiking patterns from continuously distributed ion channel densities: a computational study in spinal dorsal horn neurons. J Physiol 2018;596(9):1681-1697.

[6] Bell AM, Gutierrez-Mecinas M, Polgar E, Todd AJ. Spinal neurons that contain gastrin-releasing peptide seldom express Fos or phosphorylate extracellular signal-regulated kinases in response to intradermal chloroquine. Mol Pain 2016;12:1744806916649602.

[7] Bice TN, Beal JA. Quantitative and neurogenic analysis of the total population and subpopulations of neurons defined by axon projection in the superficial dorsal horn of the rat lumbar spinal cord. J Comp Neurol 1997;388(4):550-564.

[8] Braz J, Solorzano C, Wang X, Basbaum AI. Transmitting pain and itch messages: a contemporary view of the spinal cord circuits that generate gate control. Neuron 2014;82(3):522-536.

[9] Cai D, Cohen KB, Luo T, Lichtman JW, Sanes JR. Improved tools for the Brainbow toolbox. Nat Methods 2013;10(6):540-547.

[10] Cameron D, Polgar E, Gutierrez-Mecinas M, Gomez-Lima M, Watanabe M, Todd AJ. The organisation of spinoparabrachial neurons in the mouse. Pain 2015;156(10):2061-2071.

[11] Chamessian A, Young M, Qadri Y, Berta T, Ji RR, Van de Ven T. Transcriptional Profiling of Somatostatin Interneurons in the Spinal Dorsal Horn. Sci Rep 2018;8(1):6809.

[12] Cordero-Erausquin M, Allard S, Dolique T, Bachand K, Ribeiro-da-Silva A, De Koninck Y. Dorsal horn neurons presynaptic to lamina I spinoparabrachial neurons revealed by transynaptic labeling. J Comp Neurol 2009;517(5):601-615.

[13] Demsar J, Curk T, Erjavec A, Gorup C, Hocevar T, Milutinovic M, Mozina M, Polajnar M, Toplak M, Staric A, Stajdohar M, Umek L, Zagar L, Zbontar J, Zitnik M, Zupan B. Orange: data mining toolbox in python. Journal of Machine Learning Research 2013;14:2349-2353.

[14] Dickie AC, McCormick B, Lukito V, Wilson KL, Torsney C. Inflammatory pain reduces C fiber activity-dependent slowing in a sex-dependent manner, amplifying nociceptive input to the spinal cord. J Neurosci 2017;37(27):6488-6502.

[15] Dressler GR, Douglass EC. Pax-2 is a DNA-binding protein expressed in embryonic kidney and Wilms tumor. Proc Natl Acad Sci U S A 1992;89(4):1179-1183.

[16] Duan B, Cheng L, Bourane S, Britz O, Padilla C, Garcia-Campmany L, Krashes M, Knowlton W, Velasquez T, Ren X, Ross SE, Lowell BB, Wang Y, Goulding M, Ma Q. Identification of spinal circuits transmitting and gating mechanical pain. Cell 2014;159(6):1417-1432.

[17] Foster E, Wildner H, Tudeau L, Haueter S, Ralvenius WT, Jegen M, Johannssen H, Hosli L, Haenraets K, Ghanem A, Conzelmann KK, Bosl M, Zeilhofer HU. Targeted ablation, silencing, and activation establish glycinergic dorsal horn neurons as key components of a spinal gate for pain and itch. Neuron 2015;85(6):1289-1304.

[18] Ganley RP, Iwagaki N, Del Rio P, Baseer N, Dickie AC, Boyle KA, Polgar E, Watanabe M, Abraira VE, Zimmerman A, Riddell JS, Todd AJ. Inhibitory interneurons that express GFP in the PrP-GFP mouse spinal cord are morphologically heterogeneous, innervated by several classes of primary afferent and include lamina I projection neurons among their postsynaptic targets. J Neurosci 2015;35(19):7626-7642.

[19] Gong S, Zheng C, Doughty ML, Losos K, Didkovsky N, Schambra UB, Nowak NJ, Joyner A, Leblanc G, Hatten ME, Heintz N. A gene expression atlas of the central nervous system based on bacterial artificial chromosomes. Nature 2003;425(6961):917-925.

[20] Graham BA, Brichta AM, Callister RJ. In vivo responses of mouse superficial dorsal horn neurones to both current injection and peripheral cutaneous stimulation. J Physiol 2004;561(Pt 3):749-763.

[21] Graham BA, Brichta AM, Schofield PR, Callister RJ. Altered potassium channel function in the superficial dorsal horn of the spastic mouse. J Physiol 2007;584(Pt 1):121-136.

[22] Grudt TJ, Perl ER. Correlations between neuronal morphology and electrophysiological features in the rodent superficial dorsal horn. J Physiol 2002;540(Pt 1):189-207.

[23] Gutierrez-Mecinas M, Bell AM, Marin A, Taylor R, Boyle KA, Furuta T, Watanabe M, Polgár E, Todd AJ. Preprotachykinin A is expressed by a distinct population of excitatory neurons in the mouse superficial spinal dorsal horn including cells that respond to noxious and pruritic stimuli. Pain 2017;158(3):440-456.

[24] Gutierrez-Mecinas M, Furuta T, Watanabe M, Todd AJ. A quantitative study of neurochemically defined excitatory interneuron populations in laminae I-III of the mouse spinal cord. Mol Pain 2016;12:1744806916629065.

[25] Gutierrez-Mecinas M, Polgár E, Bell AM, Herau M, Todd AJ. Substance P-expressing excitatory interneurons in the mouse superficial dorsal horn provide a propriospinal input to the lateral spinal nucleus. Brain Struct Func 2018;*in press*.

[26] Gutierrez-Mecinas M, Watanabe M, Todd AJ. Expression of gastrin-releasing peptide by excitatory interneurons in the mouse superficial dorsal horn. Mol Pain 2014;10:79.

[27] Haring M, Zeisel A, Hochgerner H, Rinwa P, Jakobsson JET, Lonnerberg P, La Manno G, Sharma N, Borgius L, Kiehn O, Lagerstrom MC, Linnarsson S, Ernfors P. Neuronal atlas of the dorsal horn defines its architecture and links sensory input to transcriptional cell types. Nat Neurosci 2018.

[28] Harris JA, Hirokawa KE, Sorensen SA, Gu H, Mills M, Ng LL, Bohn P, Mortrud M, Ouellette B, Kidney J, Smith KA, Dang C, Sunkin S, Bernard A, Oh SW, Madisen L, Zeng H. Anatomical characterization of Cre driver mice for neural circuit mapping and manipulation. Front Neural Circuits 2014;8:76.

[29] Heinke B, Ruscheweyh R, Forsthuber L, Wunderbaldinger G, Sandkuhler J. Physiological, neurochemical and morphological properties of a subgroup of GABAergic spinal lamina II neurones identified by expression of green fluorescent protein in mice. J Physiol 2004;560(Pt 1):249-266.

[30] Huang J, Polgar E, Solinski HJ, Mishra SK, Tseng PY, Iwagaki N, Boyle KA, Dickie AC, Kriegbaum MC, Wildner H, Zeilhofer HU, Watanabe M, Riddell JS, Todd AJ, Hoon MA. Circuit dissection of the role of somatostatin in itch and pain. Nat Neurosci 2018;21(5):707-716.

[31] Hughes DI, Scott DT, Todd AJ, Riddell JS. Lack of evidence for sprouting of Abeta afferents into the superficial laminas of the spinal cord dorsal horn after nerve section. J Neurosci 2003;23(29):9491-9499.

[32] Hunt SP, Pini A, Evan G. Induction of c-fos-like protein in spinal cord neurons following sensory stimulation. Nature 1987;328(6131):632-634.

[33] Imlach WL, Bhola RF, Mohammadi SA, Christie MJ. Glycinergic dysfunction in a subpopulation of dorsal horn interneurons in a rat model of neuropathic pain. Sci Rep 2016;6:37104.

[34] Iwagaki N, Ganley RP, Dickie AC, Polgár E, Hughes DI, Del Rio P, Revina Y, Watanabe M, Todd AJ, Riddell JS. A combined electrophysiological and morphological study of neuropeptide Y-expressing inhibitory interneurons in the spinal dorsal horn of the mouse. Pain 2016;157(3):598-612.

[35] Ji RR, Baba H, Brenner GJ, Woolf CJ. Nociceptive-specific activation of ERK in spinal neurons contributes to pain hypersensitivity. Nat Neurosci 1999;2(12):1114-1119.

[36] Kim KK, Adelstein RS, Kawamoto S. Identification of neuronal nuclei (NeuN) as Fox-3, a new member of the Fox-1 gene family of splicing factors. J Biol Chem 2009;284(45):31052-31061.

[37] Kouwenhoven TA, van de Kerkhof PCM, Kamsteeg M. Use of oral antidepressants in patients with chronic pruritus: A systematic review. J Am Acad Dermatol 2017;77(6):1068-1073 e1067.

[38] Li L, Rutlin M, Abraira VE, Cassidy C, Kus L, Gong S, Jankowski MP, Luo W, Heintz N, Koerber HR, Woodbury CJ, Ginty DD. The functional organization of cutaneous low-threshold mechanosensory neurons. Cell 2011;147(7):1615-1627.

[39] Liu XY, Liu ZC, Sun YG, Ross M, Kim S, Tsai FF, Li QF, Jeffry J, Kim JY, Loh HH, Chen ZF. Unidirectional cross-activation of GRPR by MOR1D uncouples itch and analgesia induced by opioids. Cell 2011;147(2):447-458.

[40] Lu Y, Dong H, Gao Y, Gong Y, Ren Y, Gu N, Zhou S, Xia N, Sun YY, Ji RR, Xiong L. A feed-forward spinal cord glycinergic neural circuit gates mechanical allodynia. J Clin Invest 2013;123(9):4050-4062.

[41] Lu Y, Perl ER. Modular organization of excitatory circuits between neurons of the spinal superficial dorsal horn (laminae I and II). J Neurosci 2005;25(15):3900-3907.

[42] Lu Y, Perl ER. Selective action of noradrenaline and serotonin on neurones of the spinal superficial dorsal horn in the rat. J Physiol 2007;582(Pt 1):127-136.

[43] Maxwell DJ, Belle MD, Cheunsuang O, Stewart A, Morris R. Morphology of inhibitory and excitatory interneurons in superficial laminae of the rat dorsal horn. J Physiol 2007;584(Pt 2):521-533.

[44] McDonald JH. Handbook of Biological Statistics. Baltimore, Maryland: Sparky House Publishing, 2014.

[45] Melzack R, Wall PD. Pain mechanisms: a new theory. Science 1965;150(699):971-979.

[46] Mishra SK, Hoon MA. The cells and circuitry for itch responses in mice. Science 2013;340(6135):968-971.

[47] Mullen RJ, Buck CR, Smith AM. NeuN, a neuronal specific nuclear protein in vertebrates. Development 1992;116(1):201-211.

[48] Polgár E, Gray S, Riddell JS, Todd AJ. Lack of evidence for significant neuronal loss in laminae I-III of the spinal dorsal horn of the rat in the chronic constriction injury model. Pain 2004;111(1-2):144-150.

[49] Punnakkal P, von Schoultz C, Haenraets K, Wildner H, Zeilhofer HU. Morphological, biophysical and synaptic properties of glutamatergic neurons of the mouse spinal dorsal horn. J Physiol 2014;592(Pt 4):759-776.

[50] Roberson DP, Gudes S, Sprague JM, Patoski HA, Robson VK, Blasl F, Duan B, Oh SB, Bean BP, Ma Q, Binshtok AM, Woolf CJ. Activity-dependent silencing reveals functionally distinct itch-generating sensory neurons. Nat Neurosci 2013;16(7):910-918.

[51] Ruscheweyh R, Sandkuhler J. Lamina-specific membrane and discharge properties of rat spinal dorsal horn neurones in vitro. J Physiol 2002;541(Pt 1):231-244.

[52] Sardella TC, Polgár E, Watanabe M, Todd AJ. A quantitative study of neuronal nitric oxide synthase expression in laminae I-III of the rat spinal dorsal horn. Neuroscience 2011;192:708-720.

[53] Sathyamurthy A, Johnson KR, Matson KJE, Dobrott CI, Li L, Ryba AR, Bergman TB, Kelly MC, Kelley MW, Levine AJ. Massively Parallel Single Nucleus Transcriptional Profiling Defines Spinal Cord Neurons and Their Activity during Behavior. Cell Rep 2018;22(8):2216-2225.

[54] Seal RP, Wang X, Guan Y, Raja SN, Woodbury CJ, Basbaum AI, Edwards RH. Injury-induced mechanical hypersensitivity requires C-low threshold mechanoreceptors. Nature 2009;462(7273):651-655.

[55] Sikandar S, West SJ, McMahon SB, Bennett DL, Dickenson AH. Sensory processing of deep tissue nociception in the rat spinal cord and thalamic ventrobasal complex. Physiol Rep 2017;5(14).

[56] Smith KM, Boyle KA, Madden JF, Dickinson SA, Jobling P, Callister RJ, Hughes DI, Graham BA. Functional heterogeneity of calretinin-expressing neurons in the mouse superficial dorsal horn: implications for spinal pain processing. J Physiol 2015;593(19):4319-4339.

[57] Solorzano C, Villafuerte D, Meda K, Cevikbas F, Braz J, Sharif-Naeini R, Juarez-Salinas D, Llewellyn-Smith IJ, Guan Z, Basbaum AI. Primary afferent and spinal cord expression of gastrin-releasing peptide: message, protein, and antibody concerns. J Neurosci 2015;35(2):648-657.

[58] Spike RC, Puskar Z, Andrew D, Todd AJ. A quantitative and morphological study of projection neurons in lamina I of the rat lumbar spinal cord. Eur J Neurosci 2003;18(9):2433-2448.

[59] Sterio DC. The unbiased estimation of number and sizes of arbitrary particles using the disector. J Microsc 1984;134(Pt 2):127-136.

[60] Sun S, Xu Q, Guo C, Guan Y, Liu Q, Dong X. Leaky Gate Model: Intensity-Dependent Coding of Pain and Itch in the Spinal Cord. Neuron 2017;93(4):840-853.

[61] Sun YG, Chen ZF. A gastrin-releasing peptide receptor mediates the itch sensation in the spinal cord. Nature 2007;448(7154):700-703.

[62] Sun YG, Zhao ZQ, Meng XL, Yin J, Liu XY, Chen ZF. Cellular basis of itch sensation. Science 2009;325(5947):1531-1534.

[63] Szarvas S, Harmon D, Murphy D. Neuraxial opioid-induced pruritus: a review. J Clin Anesth 2003;15(3):234-239.

[64] Ting JT, Daigle TL, Chen Q, Feng G. Acute Brain Slice Methods for Adult and Aging Animals: Application of Targeted Patch Clamp Analysis and Optogenetics. In: M Martina, S Taverna, editors. Patch-Clamp Methods and Protocols. New York, NY: Springer New York, 2014. pp. 221-242.

[65] Todd AJ. Neuronal circuitry for pain processing in the dorsal horn. Nat Rev Neurosci 2010;11(12):823-836.

[66] Todd AJ. Identifying functional populations among the interneurons in laminae I-III of the spinal dorsal horn. Mol Pain 2017;13:1744806917693003.

[67] Tuckett RP. Itch evoked by electrical stimulation of the skin. J Invest Dermatol 1982;79(6):368-373.

[68] Wang D, Tawfik VL, Corder G, Low SA, Francois A, Basbaum AI, Scherrer G. Functional Divergence of Delta and Mu Opioid Receptor Organization in CNS Pain Circuits. Neuron 2018;98(1):90-108 e105.

[69] Wang X, Zhang J, Eberhart D, Urban R, Meda K, Solorzano C, Yamanaka H, Rice D, Basbaum AI. Excitatory superficial dorsal horn interneurons are functionally heterogeneous and required for the full behavioral expression of pain and itch. Neuron 2013;78(2):312-324.

[70] Ward JL. Hierarchical grouping to optimize an objective function. J Am Stat Assoc 1963;58:236-244.

[71] Yasaka T, Kato G, Furue H, Rashid MH, Sonohata M, Tamae A, Murata Y, Masuko S, Yoshimura M. Cell-type-specific excitatory and inhibitory circuits involving primary afferents in the substantia gelatinosa of the rat spinal dorsal horn in vitro. J Physiol 2007;581(Pt 2):603-618.

[72] Yasaka T, Tiong SY, Polgar E, Watanabe M, Kumamoto E, Riddell JS, Todd AJ. A putative relay circuit providing low-threshold mechanoreceptive input to lamina I projection neurons via vertical cells in lamina II of the rat dorsal horn. Mol Pain 2014;10:3.

[73] Yasaka T, Tiong SYX, Hughes DI, Riddell JS, Todd AJ. Populations of inhibitory and excitatory interneurons in lamina II of the adult rat spinal dorsal horn revealed by a combined electrophysiological and anatomical approach. Pain 2010;151:475-488.

[74] Yoshida T, Fukaya M, Uchigashima M, Miura E, Kamiya H, Kano M, Watanabe M. Localization of diacylglycerol lipase-alpha around postsynaptic spine suggests close proximity between production site of an endocannabinoid, 2-arachidonoyl-glycerol, and presynaptic cannabinoid CB1 receptor. J Neurosci 2006;26(18):4740-4751.
